# Impact of Modular Architecture on Activity of Glycoside Hydrolase Family 5 Subfamily 8 Mannanases

**DOI:** 10.3390/molecules27061915

**Published:** 2022-03-16

**Authors:** Marie Sofie Møller

**Affiliations:** Applied Molecular Enzyme Chemistry, Department of Biotechnology and Biomedicine, Technical University of Denmark, DK-2800 Kgs. Lyngby, Denmark; msmo@dtu.dk

**Keywords:** carbohydrate-active enzymes, carbohydrate-binding modules, enzyme kinetics, protein-carbohydrate interaction, plant cell wall polysaccharides

## Abstract

Glycoside hydrolase family 5 subfamily 8 (GH5_8) mannanases belong to Firmicutes, Actinomycetia, and Proteobacteria. The presence or absence of carbohydrate-binding modules (CBMs) present a striking difference. While various GH5_8 mannanases need a CBM for binding galactomannans, removal of the CBM did not affect activity of some, whereas it in other cases reduced the catalytic efficiency due to increased *K*_M_. Here, monomodular GH5_8 mannanases from *Eubacterium siraeum* (*Es*GH5_8) and *Xanthomonas citri* pv. *aurantifolii* (*Xc*GH5_8) were produced and characterized to clarify if GH5_8 mannanases from Firmicutes and Proteobacteria without CBM(s) possess distinct properties. *Es*GH5_8 showed a remarkably high temperature optimum of 55 °C, while *Xc*GH5_8 had an optimum at 30 °C. Both enzymes were highly active on carob galactomannan and konjac glucomannan. Notably, *Es*GH5_8 was equally active on both substrates, whereas *Xc*GH5_8 preferred galactomannan. The *K*_M_ values were comparable with those of catalytic domains of truncated GH5_8s, while the turn-over numbers (*k*_cat_) were in the higher end. Notably, *Xc*GH5_8 bound to but did not degrade insoluble ivory nut mannan. The findings support the hypothesis that GH5_8 mannanases with CBMs target insoluble mannans found in plant cell walls and seeds, while monomodular GH5_8 members have soluble mannans and mannooligosaccharides as primary substrates.

## 1. Introduction

Many carbohydrate-active enzymes acting on polymeric substrates, especially insoluble substrates, contain one or more non-catalytic carbohydrate-binding modules (CBMs) together with the catalytic domain. CBMs can improve catalytic function of glycoside hydrolases (GHs) through targeting the enzymes to the substrate and increasing substrate-enzyme proximity as well as disrupting the crystallinity of insoluble substrates or stabilizing the enzyme [1,2,3,4]. Generally, both mono- and multi-modular members are found within carbohydrate-active enzyme families [5]. It is unclear if enzymes composed just of the catalytic domain (CD) have different ways to interact with the substrates. In some enzyme families, so-called substrate binding sites (SBSs) situated on the CD can play the same roles as CBMs [6,7]. Monomodular enzymes represent an advantage since (i) the enzyme is less energy consuming for the organism to produce, and (ii) it is less prone to proteolytic degradation. In particular, the latter is a gain for industrial processes.

A number of endo-β-1,4-mannanases (E.C. 3.2.1.78) have been characterized due to their potential applications in different sectors of industry including detergent, textile, food, animal feed, and bioethanol [8]. They hydrolyze internal backbone β-1,4-linkages in mannans including in substituted mannans such as galactomannans from carob/locust bean (low and high viscosity; CGM-lv and CGM-hv) and guar bean (guar gum; GG), as well as in the linear konjac glucomannan (KGM) containing β-1,4-linked mannose and glucose units.

Endo-β-1,4-mannanases occur in GH families 5, 26, 45, 113, and 134 according to the Carbohydrate Active Enzymes database (CAZy; http://www.cazy.org, accessed on 8 March 2022) [5] with GH5 and GH26 mannanases being the best characterized. GH family 5 contains a wide range of enzymes acting on β-linked oligo-, polysaccharides, and glycoconjugates from a large spectrum of organisms and endo-β-1,4-mannanase activity is recognized in GH5 subfamilies 7, 8, 10, 17, 25, and 36 [9]. According to the CAZy database, subfamilies GH5_7 and GH5_8 are the largest. Only a subset of GH5_7 members have one or more CBMs (of families 2, 3, 23, 27, 35, and 65), while GH5_8 members largely contain at least one CBM (of families 2, 3, 10, 13, 32, 35, 37, 59, 64, 65) with the cellulose-binding CBM2 being the most common. The *Xanthomonas* enzymes, constituting 30% of the GH5_8 members, are very similar and clear exceptions, as they never have a CBM. GH26 includes several different specificities and among the 61 characterised GH26 mannanases, 26 have CBMs (mainly CBM35). Interestingly, CBM occurs very rarely with the two β-mannanase families GH113 and GH134 [5].

The influence of CBMs on the activity of GH5_8 mannanases has been addressed by truncation analysis. Only one monomodular GH5_8 enzyme from *Xanthomonas campestris* was studied previously [10]; however, no specific activity or kinetics data are available. Six GH5_8 members, all occurring with a CBM, have been structure determined [11,12,13,14,15,16], but only one structure, of the *Bacillus* sp. JAMB-602 mannanase (PDB entry 1WKY), included the CBM (a CBM59) [11]. Complex structures with mannooligosaccharides have been reported for three of the enzymes and none have an indication of carbohydrate binding at SBSs outside the active sites. A GH113 mannanase found without CBMs has a recognized SBSs [17].

The CD of a GH5_8 mannanase from *Saccharophagus degradans* (*Sd*GH5_8-CBM10x3) containing three CBM10s did not bind galactomannan, which was bound strongly by the full-length enzyme [18]. The same was observed for a *Bifidobacterium animalis* subsp. *lactis* Bl-0 mannanase (*Ba*GH5_8-CBM10) with one CBM10 [19]. Notably, removal of the CBM10 from *Ba*GH5_8 did not significantly affect kinetics parameters towards CGM-lv. By contrast, the kinetic parameters of *Sd*GH5_8-CBM10x3 changed upon CBM removal. However, the scenario with its three CBM10s was more complicated, as they showed distinct binding specificity and affinity [18]. A GH134 β-1,4-mannanase with a CBM10 from *Streptomyces* sp. NRRL B-24484 has been shown to bind microcrystalline cellulose, β-mannan, and chitin regardless of the presence or absence of the CBM10, which was, however, shown to be important for protein stability [20].

Here, two monomodular GH5_8 mannanases from the gut bacterium *Eubacterium siraeum* (*Es*GH5_8) and from the plant pathogen *Xanthomonas citri* pv. *aurantifolii* (*Xc*GH5_8) are recombinantly produced and biochemically characterized to investigate if monomodular GH5_8 mannanases display other properties than multimodular GH5_8 mannanases with regard to activity and polysaccharide binding. The two enzymes represent two phyla.

## 2. Results

### 2.1. Bioinformatics Analysis

**Figure 1:**
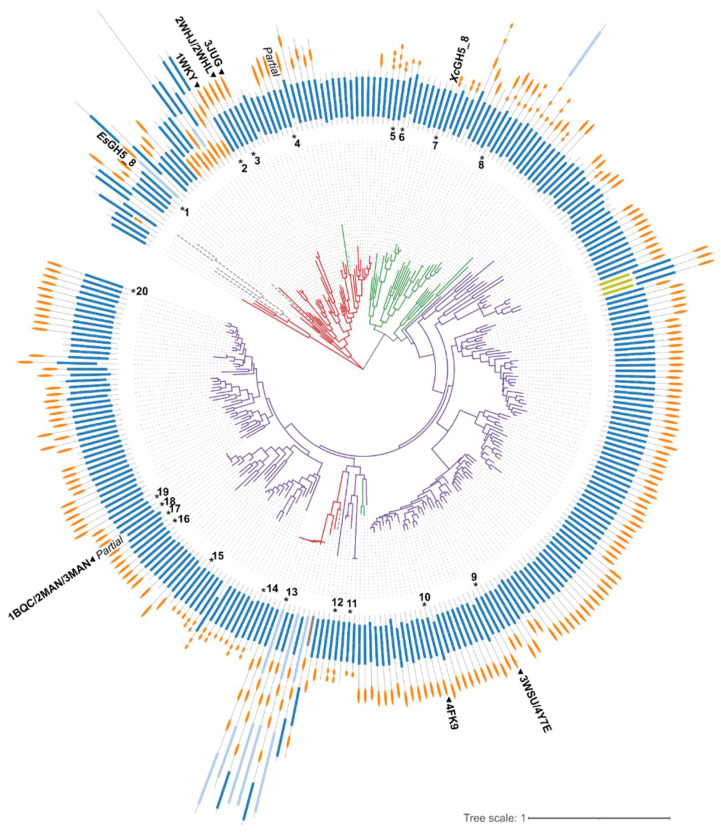
Phylogenetic tree generated based on a structure-guided multiple alignment (see Figure S1, Supplementary Materials) of the GH5_8 CDs alone. The colouring of the branches follows the origin of the protein sequence (red, Firmicutes; green, Proteobacteria; purple, Actinomycetia; grey dashed lines, other organisms including uncultured organisms). The domain architectures of the full-length proteins are show in the outer ring (dark blue, GH5_8 CD; light blue, CDs from other GH families; light green, carbohydrate esterase family 3 CD; grey, auxiliary activities family 9 CD; orange, CBMs). The numbers refer to GH5_8 enzymes with specific activity and/or kinetic analysis included here (see Table 1 and Table 2 for details). Structure determined GH5_8 members are indicated by a triangle and their PDB entries. “*Partial*” refers to only partial sequence being available. See Figure S2 for the phylogenetic tree with accession numbers and information about domain families.

The phylogenetic tree generated based on the GH5_8 CDs alone shows a clear grouping mainly based on the origin of the proteins, hence it seems like the evolution of the GH5_8 CDs reflected the taxonomy (Figure 1). Furthermore, the presence and type of CBM(s) are not necessarily following a specific pattern, though the majority of Actinomycetia GH5_8 members have one CBM, in most cases a CBM2, but with a subfraction having CBM10(s). In general, very few of the enzymes have a module N-terminally to the GH5_8 CD. Interestingly, a Firmicutes group appears among the Actinomycetia members. Out of the 298 protein sequences included in the tree, only 29 are predicted by dbCAN2 to not have a signal peptide. These intracellular proteins are distributed all over the tree and seven of them lack CBMs (data not shown).

*Es*GH5_8 is found in a small subgroup of other gut Firmicutes (*Ruminococcus* species) GH5_8 enzymes, which have remarkably different modular architecture (Figure 1). Otherwise, in *Es*GH5_8, only one more *Eubacterium* GH5_8 sequence is found in CAZy and it also lacks a CBM [5]. *Xc*GH5_8 is found in a subgroup together with monomodular GH5_8 proteins from other *Xanthomonas* species. The *Xanthomonas* protein sequences account for 230 out of the 785 members of GH5_8 in the CAZy database [4] and none of them contain a CBM.

**Figure 2:**
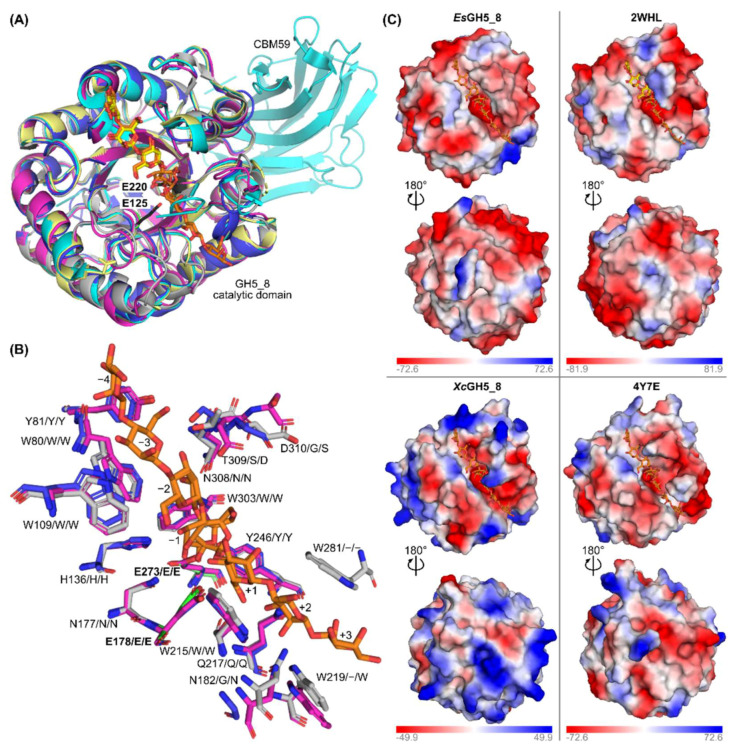
Comparison of the AlphaFold models of *Es*GH5_8 and *Xc*GH5_8 and the structures of GH5_8 from *Bacillus agaradhaerens* (PDB entry 2WHL), *Bacillus* sp. JAMB-602 (PDB entry 1WKY), and *Streptomyces thermolilacinus* (PDB entry 4Y7E). (**A**) Superimposition of the two models (*Es*GH5_8, dark blue; *Xc*GH5_8, pink) and three structures (1WKY, cyan; 2WHL, pale yellow; 4Y7E, grey). The ligands from 2WHL (yellow) and 4Y7E (orange) is shown as sticks, while the catalytic residues of 2WHL are shown as black sticks. (**B**) Comparison of subsite residues of *S. thermolilacinus* (green sticks, catalytic residues; grey sticks, other subsite residues; orange, mannooligosaccharide ligands) (Kumagai 2015) with *Es*GH5_8 (blue sticks) and *Xc*GH5_8 (pink sticks). Residue numbers refer to PDB entry 4Y7E, while following residue letters refer to *Es*GH5_8 and *Xc*GH5_8, respectively. (**C**) Comparison of the electrostatic surface of the two models and the two related structures. The mannooligosaccharide ligands of 4Y7E is superimposed on all models and structures (orange sticks), while the ligand (mannotriose) of 2WHL is shown as yellow sticks.

The models of *Xc*GH5_8 and *Es*GH5_8 were predicted using AlphaFold 2 and, as expected, were overall very similar to known GH5_8 structures (Figure 2A). The root mean square deviation (RMSD) between the models of *Es*GH5_8 and *Xc*GH5_8 was 0.652 Å^2^, while it was 0.432–0.596 Å^2^ between the models and the structure determined GH5_8 mannanases included in Figure 2. A comparison of subsite residues of the *Streptomyces thermolilacinus* GH5_8 structure (PDB entry 4Y7E) [15], which has ligands covering both plus (aglycone) and minus (glycone) subsites, with the two models showing that most residues are conserved; although, residues around subsites +2 and +3 differ/are absent (Figure 2B). Furthermore, the residues proposed by Kumagai et al. to be involved in interaction with galactose branches in the substrate differ (residues 308–310) [15]. *Es*GH5_8 is closely related to the GH5_8 structures of other Firmicutes, while for *Xc*GH5_8 it is important to note, that there is no available structure of a GH5_8 from the Actinomycetia phylum. When the electrostatic surface of the model of *Xc*GH5_8 was compared with one of the closest related structures from *S. thermolilacinus* (PDB entry 4Y7E), it was clearly different. *Xc*GH5_8 has large positively charged patches (Figure 2C), also in agreement with the high pI predicted to 9.0. Moreover, it had more of a closed active site cleft as compared to the structure of *S. thermolilacinus* (PDB entry 4Y7E). *Es*GH5_8 resembled the other Firmicutes GH5_8 structure (PDB entry 2WHL) with a relatively long and open active site cleft (Figure 2C).

### 2.2. Enzymatic Activity of EsGH5_8 and XcGH5_8

**Figure 3:**
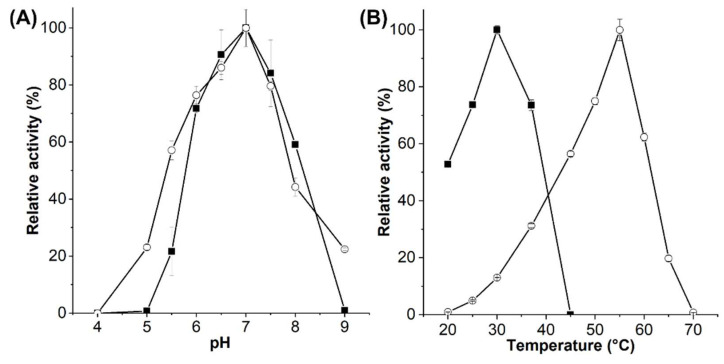
pH (**A**) and temperature (**B**) optimum of *Es*GH5_8 (■) and *Xc*GH5_8 (○).

*Es*GH5_8 and *Xc*GH5_8 were produced and purified to homogeneity in yields of 14.3 and 2.6 mg/g cells, respectively. The pH optimum of both *Es*GH5_8 and *Xc*GH5_8 was 7, and the optimal activity was around 30 °C of *Xc*GH5_8 and 55 °C of *Es*GH5_8 (Figure 3). Furthermore, melting temperatures (*T*_m_) of *Xc*GH5_8 and *Es*GH5_8 were determined by differential scanning fluorimentry to 37.9 °C and 57.8 °C, respectively (Figure S3). While the specific activity and kinetic parameters of *Xc*GH5_8 were determined at its optimal temperature, *Es*GH5_8 was analysed at 37 °C, since most mannanases despite their activity temperature optimum are assayed at either 37 °C or 40 °C, which was also a biologically relevant temperature as *Es*GH5_8 is a gut bacterium. *Es*GH5_8 showed equally good activity towards CGMs and KGM, whereas *Xc*GH5_8 had a clear preference for CGMs (Table 1). Both enzymes showed low activity on the more densely branched guar gum, as compared with other GH5_8 mannanases (Table 1). *Xc*GH5_8 did not degrade ivory nut mannan (INM), and *Es*GH5_8 had low activity on INM, as compared to the few other GH5_8 mannanases characterized on this substrate (Table 1). None of the enzymes had activity on xanthan from *X. campestris*.

**Table 1:** Specific activity of GH5_8 mannanases. Enzymes are listed according to the groups on the phylogenetic tree (Figure 1).

Origin (GenBank Accession; No. in Phylogenetic Tree)	Modular Structure of Charac. Protein	CGM-lv U/mg; 1/s (Relative ^1^, %)	CGM-hv U/mg; 1/s (Relative ^1^, %)	KGM U/mg 1/s (Relative ^1^, %)	GG U/mg; 1/s (Relative ^1^, %)	INM U/mg; 1/s (Relative ^1^, %)	Ref.
*Eubacterium siraeum* (CBK96294; #1)	GH5_8	625 ± 28.8; 397 ± 18.3 (97)	578 ± 13.0; 367 ± 8.3 (90)	644 ± 11.4; 409 ± 7.2 (100)	3.6 ± 0.2; 2.3 ± 0.1 (0.6)	7.5 ± 0.1; 4.8 ± 0.1 (1.2)	Present study
*Saccharophagus* *degradans* (ABD79918; #5)	GH5_8-CBM10x3	1972 ± 80; 1729 ± 70 (56)	2212 ± 78; 1939 ± 68 (62)	3544 ± 110; 3107 ± 96 (100)	40 ± 8; 35 ± 7 (1.1)	9 ± 1; 8 ± 1 (0.3)	[18]
	GH5_8-ΔCBM10x3	2906 ± 53; 1695 ± 31 (64)	3151 ± 304; 1838 ± 177 (69)	4556 ± 108; 2658 ± 63 (100)	108 ± 25; 63 ± 15 (2.4)	81 ± 7; 47 ± 4 (1.8)	[18]
*Xanthomonas citri* (AMU98328; #7)	GH5_8	713 ± 12.0; 414 ± 7.0 (100)	709 ± 23.0; 411 ± 13,3 (99)	247 ± 19.3; 143 ± 11.2 (35)	1.1 ± 0.1; 0.6 ± 0.1 (0.2)	N.D. ^2^	Present study
*Bifidobacterium animalis* (ACS46797; #11)	GH5_8-CBM10	1380; 872 (55)	1920; 1213 (76)	2520; 1592 (100)	N.D. ^2^	120; 76 (5)	[19]
*Cellulosimicrobium* sp. strain HY-13 (AEE43708; #12)	GH5_8-CBM10x2	8498 ± 105; 6232 ± 77 (58)	-	-	967 ± 18; 709 ± 13 (6.6)	14,711 ± 183; 10,788 ± 134 (100)	[21]
*Streptomyces* sp. S27 (ADK91085; #14)	GH5_8-CBM10	2107 ± 182; 1510 ± 130 (100)	-	1312 ± 110; 940 ± 79 (62)	74 ± 12; 53 ± 8.6 (3.5)	-	[22]
*Streptomyces lividans* (AAA26710; #15)	GH5_8-CBM10	141 ± 1.7 ^3,4^ (100)	-	55 ± 0.7 ^4^ (39)	21 ± 1.7 ^4^ (15)	18.8 ± 1.2 ^4^ (13)	[23]
	GH5_8-ΔCBM10	97 ± 1.4 ^3,4^ (100)	-	61 ± 0.45 ^4^ (63)	23 ± 3.1 ^4^ (23)	19 ± 0.7 ^4^ (20)	[23]
*Streptomyces* *thermoluteus* (BAM62868; #20)	GH5_8-CBM2	51 ± 1.6 ^3,4^ (78)	-	66 ± 1.5 ^4^ (100)	27 ± 1.4 ^4^ (41)	20.5 ± 0.4 ^4^ (31)	[23]
	*St*GH5_8-ΔCBM2	39 ± 0.6 ^3,4^ (86)	-	45 ± 0.5 ^4^ (100)	20 ± 3.7 ^4^ (44)	14 ± 1.3 ^4^ (32)	[23]

^1^ Relative to the best substrate of a given enzyme. ^2^ No activity detected. ^3^ It is not clear if assay was performed with low- or high-viscosity CGM. ^4^ 1/s.

**Figure 4:**
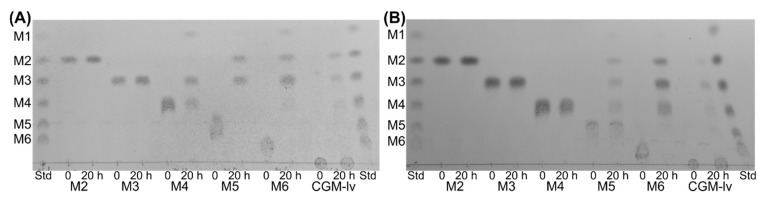
Thin layer chromatography (TLC) of the hydrolytic products generated by *Es*GH5_8 (**A**) and *Xc*GH5_8 (**B**) on mannooligosaccharides (mannobiose, M2, to mannohexaose, M6) and CGM-lv. The standard (Std) contained a mix of linear mannooligosaccharides (mannose to mannohexaose; M1–M6).

*Es*GH5_8 and *Xc*GH5_8 showed different hydrolysis patterns of linear mannooligosaccharides (Figure 4): None of the enzymes hydrolysed mannobiose or mannotriose, but *Es*GH5_8 hydrolysed mannotetraose into mannose and mannotriose, while *Xc*GH5_8 showed no activity on this oligosaccharide. Furthermore, both enzymes hydrolysed mannopentaose and mannohexaose, but the product profiles differed: *Es*GH5_8 released solely mannobiose and mannotriose from mannopentaose, while *Xc*GH5_8 released mannose to mannotetraose. *Es*GH5_8 hydrolysed mannohexaose hydrolysed to mannose, mannobiose, and mannotriose, while *Xc*GH5_8 produced mannobiose, mannotriose, and mannotetraose. This profile of *Xc*GH5_8 on mannohexaose is in line with its inability to hydrolyse mannotetraose. The hydrolysis profile of CGM-lv is similar for the two enzymes (Figure 4).

Kinetic analysis of the two enzymes on CGM-lv gave a Michaelis–Menten constant (*K*_M_) for *Es*GH5_8 of 4.6 mg/mL and 2.6 mg/mL for *Xc*GH5_8 (Table 2). *Es*GH5_8 had a slightly higher highest turn-over number (*k*_cat_), but its relatively higher *K*_M_ resulted in the lower catalytic efficiency (*k*_cat_/*K*_M_) of these two monomodular GH5_8 enzymes.

**Table 2:** Kinetic parameters for GH5_8 enzymes on CGM-lv. The enzymes listed according to the groups of the phylogenetic tree (Figure 1).

Origin (Genbank Accession; No. in Phylogenetic Tree)	Modular Structure of Charac. Protein	*K*_M_ (mg/mL)	*k*_cat_ (1/s)	*k*_cat_/*K*_M_ (mg/(mL s)	Binding CGM?	Ref.
*Eubacterium siraeum* (CBK96294; #1)	GH5_8	4.6 ± 0.5	850 ± 47	185 ± 23	Yes, weakly	Present study
*Bacillus* sp. JAMB-602 (BAD99527; #2)	GH5_8-CBM59	3.1	135 ^1^		ND ^2^	[24]
*Bacillus agaradhaerens* (AAN27517; #3)	GH5_8-CBM59	1.8	633	250	ND ^2^	[13]
*Bacillus nealsonii* PN-11 (AGU71466; #4)	GH5_8-CBM59	7.2 ± 0.3	750 ± 55 ^3^	104 ± 2 ^3^	ND ^2^	[25]
*Saccharophagus degradans* (ABD79918; #5)	GH5_8-CBM10x3	2.1 ± 0.1	2333 ± 55	1096 ± 71	Yes, *K*_d_ < 0.125 mg/mL	[18]
	*Sd*GH5_8-ΔCBM10x3	2.4 ± 0.1	3440 ± 75	1413 ± 82	No	[18]
*Cellvibrio japonicus* (ACE84673/AAO31759; #6)	GH5_8-ΔCBM10x2	8.5 ± 1.5	2381 ± 66	246.0	No	[26]
*Xanthomonas citri* (AMU98328; #7)	GH5_8	2.6 ± 0.3	732 ± 36	282 ± 35	ND ^2^	Present study
*Cellvibrio japonicus* (AAO31760; #8)	GH5_8-ΔCBM10-CBM2	2.2 ± 0.3	1075 ± 27	446	No	[26]
*Streptomyces thermolilacinus* (BAK26781; #9)	GH5_8-ΔCBM2	4.9 ± 1.0	21 ± 2	4 ± 1	ND ^2^	[15,27]
*Streptomyces* sp. SirexAA-E (AEN10237; #10)	GH5_8-Fn3-CBM2	2 ± 0.2	41 ± 2	21 ± 10	Yes, weakly	[14]
	GH5_8-ΔFn3-CBM2	2 ± 0.4	41 ± 3	21 ± 8	No	[14]
*Bifidobacterium animalis* (ACS46797; #11)	GH5_8-CBM10	1.6 ± 0.2	1828 ± 87	1157 ± 177	Yes, *K*_d_ = 0.31 mg/ml	[19]
	GH5_8-ΔCBM10	1.8 ± 0.5	2005 ± 179	1146 ± 324	No	[19]
*Caldicellulosiruptor bescii* (ACM60953; #13)	GH9-CBM3x3-GH5_8	0.6 ± 0.3	1420 ± 158	2290	ND ^2^	[28]
	CBM3x3-GH5_8	1.8 ± 0.5	3446 ± 367	1893	ND ^2^	[28]
*Streptomyces* sp. S27 (ADK91085; #14)	GH5_8-CBM10	0.16	3739 ^3^	23369 ^3^	ND ^2^	[22]
*Streptomyces lividans* (AAA26710; #15)	GH5_8-CBM10	3.5 ± 0.5	197 ± 11	60 ± 7	ND ^2^	[23]
	GH5_8-ΔCBM10	4.3 ± 0.7	139 ± 9	33 ± 4	ND ^2^	[23]
*Thermobifida halotolerans* (AHB89704; #16)	GH5_8-CBM2	1.3 ± 0.3	78 ± 9	60	ND ^2^	[29]
*Thermobifida cellulosilytica* (AHB89703; #17)	GH5_8-CBM2	0.8 ± 0.2	89 ± 5	106	ND ^2^	[29]
*Thermobifida fusca* (AAZ54938; #18)	GH5_8-ΔCBM2	10.4 ± 2.6	96 ± 14	9 ± 3	ND ^2^	[15]
*Thermobifida fusca* TM51 (AHB89702; #19)	GH5_8-CBM2	1.7 ± 0.4	122 ± 11	74	ND ^2^	[29]
*Streptomyces thermoluteus* (BAM62868; #20)	GH5_8-CBM2	5.5 ± 1.6	101 ± 17	18 ± 3	ND ^2^	[23]
	GH5_8-ΔCBM2	5.5 ± 1.5	75 ± 12	14 ± 2	ND ^2^	[23]

^1^*V*_max_ (mg mannose/min/mg protein). ^2^ Not determined. ^3^ *V*_max_ (µmol/mL/min) and *V*_max_/*K*_m_ (µmol/min/mg).

### 2.3. Polysaccharide Interaction

**Figure 5:**
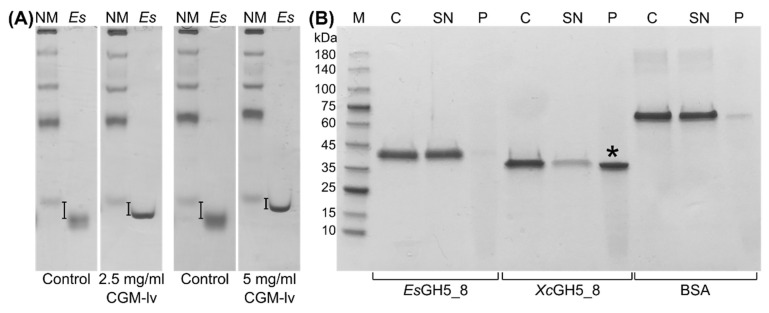
Analysis of binding of *Es*GH5_8 and *Xc*GH5_8 to polysaccharides. (**A**) Affinity gel-electrophoresis of *Es*GH5_8 (*Es*) at 2.5 and 5.0 mg/mL CGM-lv in the gel (NM, native marker functioning as reference). Control gels without CGM-lv are at the left. (**B**) Qualitative pull-down assay with INM (M, marker; C, the protein stock used for assay acting as a control; SN, supernatant; P, pellet). Asterisk indicates the significant binding of *Xc*GH5_8 to INM. The original gels can be found in Figure S4.

Binding between *Es*GH5_8 to soluble CGM-lv was analysed using affinity gel electrophoresis (AGE). The migration of *Es*GH5_8 was slightly retarded in a CGM-lv concentration dependent manner (Figure 5A), but a binding constant could not be determined due to a combination of too low affinity and too high viscosity of CGM-lv at higher concentrations (highest in-gel concentration tested was 5 mg/mL). The high pI (9.0) of *Xc*GH5_8 prevented use of the conventional AGE setup [30] and various alternative attempts were not successful. However, using a pull-down assay it was shown that *Xc*GH5_8 bound to INM, whereas both *Es*GH5_8 and the control bovine serum albumin (BSA) showed very weak binding (Figure 5B). None of the proteins bound to microcrystalline cellulose (Avicel) or starch granules (data not shown).

## 3. Discussion

The *Es*GH5_8 and *Xc*GH5_8 represent the first well-characterized monomodular GH5_8 mannanases. Though, temperature and pH optima were reported of a close *Xc*GH5_8 homologue from *X. campestris* studied due to its function as virulence factor [10]. It had a temperature optimum around 37 °C, thus slightly higher than *Xc*GH5_8 (Figure 3B), while both enzymes had a pH optimum of 7. *Es*GH5_8 has a remarkably high temperature optimum and *T*_m_. The *S. lividans* GH5_8 with one CBM10 had a comparable *T*_m_ (60 °C) in the presence of calcium ions, but this decreased by approximately 15 °C after treatment with EDTA. Furthermore, it was concluded that the CBM10 was not affecting the thermal stability [23]. GH5_8 mannanases from the thermotolerant *Thermobifida* bacteria have temperature optima at 70–75 °C [29]. However, their turn-over numbers were in the range of 80–120 1/s at 50 °C, hence 7–10-fold lower than that of *Es*GH5_8 at 37 °C (Table 2), thus far below its optimal temperature.

Notably, *Es*GH5_8 degrades CGM and KGM equally good (Table 1). This is in contrast to the other characterized GH5_8 mannanases, which do prefer either CGM or KGM (Table 1). This seems to be reflected in the kinetics of CGM hydrolysis, as *Es*GH5_8 has a relatively high *K*_M_, but still displays better catalytic efficiency than the *Thermobifida* mannanases and the majority of the *Streptomyces* mannanases (Table 2). Some of the *Streptomyces* mannanases even show clear preferences for CGM (Table 1). Hence, *Es*GH5_8 is an interesting candidate for industrial applications due to its remarkable temperature stability and its high activity on both CGM and KGM.

The GH5_8 endo-β-1,4-mannanase from *X. campestris* was shown to be required for full virulence of the bacterium to plants. It was suggested that the role of the enzyme during disease is to promote transitions from an aggregated or biofilm lifestyle to a planktonic lifestyle [10,31]. However, the enzyme cannot degrade the mannose-containing exopolysaccharide xanthan produced by *X. campestris* [31]. In the present study, neither *Xc*GH5_8 nor *Es*GH5_8 could degrade xanthan (data not shown).

Interestingly, the product profile of mannooligosaccharide hydrolysis by *Es*GH5_8 and *Xc*GH5_8 was different indicating differences in subsite availability. The complete lack of activity of *Xc*GH5_8 on mannotetraose is uncommon among the characterized GH5_8 mannanases. Notably, the observation is in agreement with mannotetraose being a hydrolysis product from mannohexaose. In the case of *Es*GH5_8, no mannotetraose is observed, most likely because it is efficiently hydrolysed. Usually, some activity of GH5_8 mannanases is detected on mannotetraose, though the activity generally increases significantly when increasing the length to 5 and 6 mannose units [13,15,19,26].

The kinetics of the *Thermobifida* enzymes clearly show that their CBM2 is important for activity. Thus, removal of CBM2 by truncation induced a six-fold increase of *K*_M_, and hence a crucial loss of efficiency (Table 2). The CBM is also important for activity of other characterized Actinomycetia GH5_8, but without similar strong impact on *K*_M_ and the effect being seen on the turn-over number (Table 2). Interestingly, the removal of the CBM10 influenced the substrate preference of mannanase from *S. lividans*; the activity being reduced for the favored CGM and increased on KGM (Table 1).

*Es*GH5_8 is found in a subgroup of the phylogenetic tree with proteins having a very diverse modular architecture (Figure 1), but all originating from gut bacteria. This suggests that the catalytic domain evolved first and that the additional modules were incorporated later in evolution.

Interestingly, *Xc*GH5_8 did not degrade INM, but could bind to INM, unlike *Es*GH5_8. The active site of both enzymes are predicted to have the same cleft-like shape (Figure 2B), hence the differences in activity on INM cannot be easily explained. Importantly, the interaction between *Xc*GH5_8 and INM seemed to be specific, as no interaction was detected with microcrystalline cellulose (β-1,4-linked glucose units) or granular starch (α-1,4-linked glucose units). Notably, the activity of *Es*GH5_8 on INM was in the same range as of the full-length enzyme from *S. degradans*, the CD of which showed about a ten-fold better activity (Table 1). There seems to be no real pattern with regard to the effect of the presence of CBM on the activity on insoluble INM. Full-length GH5_8-CBM10x2 from *C. japonicus* bound to crystalline mannan, while the CD alone did not. The same was seen for the other *C. japonicus* mannanase (GH5_8-CBM10-CBM2), here CBM10 was important for mannan binding. In addition, none of the CBMs from the two *C. japonicus* mannanases was able to bind soluble CGM or KGM [26]. Unfortunately, the catalytic constants were only determined for the CDs alone (Table 2), which did not act on INM [26]. Interestingly, a mannanase (GH5_8-CBM10x2) from *Cellulosimicrobium* sp. HY-13 was also shown to bind INM as well as having the best specific activity towards INM (1.7-fold better than on CGM) (Table 1) [21].

The CBM2 and CBM10 of GH5_8 mannanases are known to facilitate binding to cellulose, despite the enzymes not being active on cellulose [14,18,21,26]. It has been suggested that the function of these CBMs is to target the enzymes to the mannan-containing plant cell-wall through interaction with the simple crystalline cellulose surface [18]. Along these lines, it is suggested that the GH5_8 mannanases with CBMs target insoluble mannans in plant cell-walls and seeds, while the monomodular GH5_8 members have soluble mannans and mannooligosaccharides as their primary substrates [13]. The latter also goes for GH5_8 members having CBMs not binding to insoluble polysaccharides, i.e., the mannanase from *B. animalis*, which has a CBM10 only capable of binding CGM but not insoluble cellulose or INM [19]. The monomodular group and the group of GH5_8 with CBMs not binding to insoluble polysaccharides might act together with other enzymes capable of degrading the insoluble mannans.

## 4. Materials and Methods

### 4.1. Carbohydrates

CGM-lv, INM, KGM, and linear β-1,4-mannooligosaccharides were from Megazyme (Wicklow, Ireland); CGM-hv (locust bean gum), GG, xanthan from *Xanthomonas campestris*, Avicel (microcrystalline cellulose), and unmodified wheat starch were from Sigma-Aldrich (Darmstadt, Germany).

### 4.2. Bioinformatics Analysis of GH5_8

Protein sequences for all GH5_8 proteins in the CAZy database [1] (778 sequences) were retrieved from NCBI and redundancy was reduced using CD-HIT [32] with a 90% identity cut-off. It was ensured that characterized protein sequences were maintained in the dataset. The resulting dataset contained 298 sequences. A structure-based alignment was generated using PROMALS3D [33] including only the GH5_8 catalytic domain (as predicted by dbCAN2 [34]). Representative structures of the six structures determined GH5_8 enzymes guided the alignment. Phylogenetic analysis was performed using the maximum likelihood method and the bootstrapping procedure with 500 bootstrap trials from the MEGA11 software suite [35]. The tree was displayed with the Interactive Tree Of Life (iTOL) online tool (https://itol.embl.de/ accessed on 8 March 2022) [36].

Models of *Es*GH5_8 and *Xc*GH5_8 were predicted using AlphaFold run in the Colab notebook version [37].

### 4.3. Recombinant Protein Production

The genes encoding *Es*GH5_8 (GenBank accession no.: CBK96294) and *Xc*GH5_8 (GenBank accession no.: AMU98328) without predicted signal peptides (amino acids 1–27 and 1–26, respectively) were purchased (GenScript, Leiden, The Netherlands) sub-cloned into pET28a using the NheI and XhoI restriction sites resulting in a cleavable N-terminal His-tag. The plasmids were transformed into *E. coli* BL21. Starter cultures (10 mL) were made by inoculating LB medium including 50 µg/mL kanamycin with a single colony and incubated at 37 °C overnight. LB medium (750 mL) containing 10 mM glucose and 50 µg/mL kanamycin in shake flask was inoculated with the overnight culture and propagated (37 °C, 160 rpm) to an absorbance at 600 nm of 0.6. The temperature was decreased to 16 °C and expression was induced by a final concentration of 0.1 mM IPTG. Cells were harvested (4000× *g*, 20 min, 4 °C) after approximately 20 h and stored at −20 °C until protein purification.

### 4.4. Protein Purification

The proteins were purified in two steps. Cells were resuspended in HisTrap equilibration buffer (10 mM Hepes, pH 7.4, 10 mM imidazole, 0.5 M NaCl, and 10% glycerol), lysed using a high-pressure homogenizer at 1 bar, added 3 μL of Benzonase Nuclease (Sigma-Aldrich), and centrifuged (40,000× *g*, 4 °C, 30 min). The supernatant was loaded onto a 5 mL HisTrap HP column (Cytiva, Marlborough, MA, USA) pre-equilibrated with HisTrap equilibration buffer and eluted using HisTrap elution buffer (10 mM Hepes, pH 7.4, 320 mM imidazole, 0.5 M NaCl, and 10% glycerol). The eluate was further purified by gel filtration (Superdex 16/60 75). *Es*GH5_8 was purified using 10 mM Hepes pH 7.4, 150 mM NaCl, while *Xc*GH5_8 was purified using 10 mM Hepes at a pH of 7.0, 150 mM NaCl, and 10% glycerol. Fractions containing pure recombinant protein of correct size as based on SDS-PAGE were pooled and concentrated to 2–3 mg/mL (10 kDa Amicon Ultra; Merck-Millipore, Darmstadt, Germany)). *Xc*GH5_8 was stored in 10 mM Hepes pH 7.0, 150 mM NaCl, and 30% glycerol, while *Es*GH5_8 was stored in the gel filtration buffer. The absorbance at 280 nm was measured, and protein concentration was determined using theoretical extinction coefficients (*Es*GH5_8, 78,380 M^−1^ cm^−1^ and molecular weight 38.1 kDa; *Xc*GH5_8, 68,410 M^−1^ cm^−1^ and molecular weight 34.8 kDa).

### 4.5. Determination of Melting Temperature

The melting temperatures (Tm) of *Es*GH5_8 and *Xc*GH5_8 were determined by differential scanning fluorimetry using a Prometheus Panta instrument (NanoTemper Technologies, München, Germany). Fluorescence at 330 nm and 350 nm upon excitation at 280 nm were measured using Prometheus NT.48 High Sensitivity capillaries (NanoTemper Technologies) with a protein concentration of 2 µM. The excitation power was determined from a pre-scan of the samples and set to 61%. The unfolding was followed by ramping the temperature from 20 to 95 °C with a temperature increment of 1 °C/min.

### 4.6. Enzymatic Assays

Enzymatic activity was determined using a standard reducing end 3,5-dinitrosalicylic acid (DNS) assay essentially as previously described [18,19,38]: 360 µL of substrate in assay buffer was preincubated at a given temperature for 5 min before the reaction was initiated by addition of 40 µL of enzyme solution in assay buffer. The reaction was terminated by addition of 600 µL of DNS reagent followed by heat treatment at 95 °C for 15 min. After 15 min in ice water, the samples were centrifuged at 20,000× *g* for 10 min. Finally, the absorbance was measured at 540 nm. Mannose was used as standard. One unit of mannanase activity was defined as the amount of enzyme that liberates reducing sugars equivalent to one µmol mannose per minute.

pH optimum was determined using the standard assay in triplicate using a final concentration of 2.5 mg/mL CGM-lv with enzyme (*Es*GH5_8, 7.4 nM; *Xc*GH5_8, 15.3 nM) in a universal buffer (10 mM Hepes, 10 mM MES, 10 mM sodium acetate, and 75 mM NaCl) [39] in the range from pH 4 to 9.

The temperature optimum was determined using the standard assay with 2.5 mg/mL of CGM-lv at different temperatures (*Xc*GH5_8, 20–45 °C; *Es*GH5_8, 20–65 °C) in 50 mM sodium phosphate-citrate pH 7.0 including 0.005% Triton X-100, as a buffer.

The specific activity was determined using the standard assay with 50 mM sodium phosphate-citrate pH 7.0, 0.005% Triton X-100, as buffer at 37 °C (*Es*GH5_8) and 30 °C (*Xc*GH5_8). *Es*GH5_8 (3.5–28 nM) and *Xc*GH5_8 (5.1–51.1 nM) were incubated with low- and high-viscosity CGMs (2.5 mg/mL, 10 min), KGM (2.5 mg/mL, 10 min), GG (2.5 mg/mL, 2.5 h), xanthan (2.5 mg/mL, 2.5 h), or INM (5 mg/mL, 45 min) in triplicate.

The kinetics on CGM-lv were determined using the same buffer and temperatures as for specific activity determination with a range of substrate concentrations (0.45–9 mg/mL CGM-lv) and enzyme (*Es*GH5_8, 5.0 nM; *Xc*GH5_8, 5.4 nM) in 2000 μL and withdrawing 400 μL aliquots at 3, 6, 9, and 12 min. The kinetic assays were done in triplicate. The Michaelis–Menten model was fitted to the initial velocity data using GraphPad Prism 6 (GraphPad Software Inc., San Diego, CA, USA).

### 4.7. Thin Layer Chromatography (TLC)

The product profile from hydrolysis of mannooligosaccharides was analysed using TLC: 50 µL of the reaction mixture, containing 2 mM of mannooligosaccharide (mannobiose to mannohexaose) or 2.5 mg/mL of CGM-lv and enzyme (*Es*GH5_8, 7 nM; *Xc*GH5_8, 10 nM) in 50 mM sodium phosphate-citrate pH 7.0, 0.005% Triton X-100, was incubated at 25 °C (*Xc*GH5_8) or 37 °C (*Es*GH5_8) for 20 h followed by 10 min at 95 °C to inactivate the enzyme. A sample (2 µL) was applied twice on a silica gel 60 F454 plate (Merck Millipore) and 2 µL of a mixture of 2 mM mannooligosaccharides (mannose to mannohexaose) was applied as standard. The separation was carried out in isopropanol:n-butanol:water (12:4:5) (*v*/*v*) as the mobile phase and run twice. Sugars were visualized with a diphenylamine alanine solution (12 mM diphenylamine, 2% aniline, and 8.5% phosphoric acid in methanol) and heat treatment.

### 4.8. Affinity Gel-Electrophoresis (AGE)

Qualitative screening of binding of *Es*GH5_8 was performed by AGE with 2.5 mg/mL and 5 mg/mL of CGM-lv in 10% acrylamide gels without stacking gels at pH 7.4 (43 mM imidazole and 35 mM Hepes) [30]. Protein (5 μL 0.4 mg/mL) in loading buffer was applied to polysaccharide-containing and control (without polysaccharide) gels, respectively. NativeMark unstained protein standard (Invitrogen) was included on all gels for normalization. The gels were run (4 h at 4 °C and 100 V) and stained using InstantBlue (Expedeon, Cambridge, UK).

### 4.9. Pull-Down Assay

A qualitative screening of the binding of *Es*GH5_8 and *Xc*GH5_8 to insoluble crystalline INM, microcrystalline cellulose (Avicel), and wheat starch, was performed by a pull-down assay, where the samples were analysed by SDS-PAGE: 10 mg insoluble polysaccharide (prewashed with buffer three times) was mixed with 200 μL of 0.5 mg/mL protein in an assay buffer (50 mM sodium phosphate-citrate buffer pH 7.0, 0.005% Triton X-100). Bovine serum albumin (BSA; Sigma-Aldrich) was used as a control. The samples were incubated at 4 °C for 1 h with gentle agitation and then centrifuged (20,000× *g*, 10 min, 4 °C). Supernatants were transferred to fresh tubes and centrifuged again, before 4 μL of a supernatant was heat treated in the presence of SDS-loading buffer and subjected to SDS-PAGE. The pellets from the pull-down assay were washed with 250 μL of assay buffer and pelleted as aforementioned, resuspended in 200 μL of assay buffer, added 50 μL of SDS-loading buffer, boiled for 10 min, and applied (5 μL) on the SDS-PAGE. For each protein, a sample of the 0.5 mg/mL protein stock used for the pull-down assay was treated as the supernatant sample and included on the gel.

## Data Availability

All available data is included in the article.

## Supplementary Materials

The following are available online at https://www.mdpi.com/article/10.3390/molecules27061915/s1, Figure S1: Structure-guided multiple sequence alignment of GH5_8 catalytic domains; Figure S2: Phylogenetic tree with accession and domain family numbers; Figure S3: Differential scanning fluorimetry (DSF) results for *Es*GH5_8 and *Xc*GH5_8. Figure S4: Original AGE gels related to Figure 5.

## Sample Availability

All compounds used in the study is commercially available.
