# Supplementary material for: Impact of Modular Architecture on Activity of Glycoside Hydrolase Family 5 Subfamily 8 Mannanases
Source: Molecules. 2022 Mar 16;27(6):1915. doi: 10.3390/molecules27061915 (PMC8952944; doi:10.3390/molecules27061915)
Supplement: Supplementary file 1 [file molecules-27-01915-s001.zip › Figure_S1.pdf]

Figure S1: Structure-guided multiple sequence alignment of GH5_8 catalytic domains. The alignment was generated using PROMALS3D used for the phylogenetic analysis. The alignment is visualised using ESPript 3.0.

```
             1        10        20        30        40        50        60        70        80        90        100       110       120
ADU21877.1 .DANGNTFVMRGVNIAHAWYSS....YTSTSIKGAADNGANTVRVVV.ADGK.......KWSK...TSKSDLQSIVNECKKNDLVCI.LEVH.DATGSD........STSDLKAAVDYWIE..NKS.VLQGNEKYVILNIANEWYGS..WN
ADU23074.1 .DANNEPFVMRGVNIAHAWYQN....DTNTSVIGAAENGSNTVRVVV.ADGQ.......KWSK...TSKSELENIINICKENDVICI.LEVH.DATGSN........NTYDLDNAVNYWIE..NKS.VLQGNEEYVILNIANEWYGE..WN
CCO04360.1 .DASGNKFVMRGVNIAHAWYTE....KTETSIKGAANNGANTVRVVL.ANGS.......KYTK...TSAQEVKNIISWCKSNKLICI.LEVH.DATGSD........STYDLNKCVDYWKE..MKS.VLSGTEKYVIVNIANEWYGT..WN
BAA25878.1 .DATGQPFVMRGVNHAHTWYKD....QLSTAIPAIAKTGANTIRIVL.ANGH.......KWTL...DDVNTVNNILTLCEQNKLIAV.LEVH.DATGSD........SLSDLDNAVNYWIG..IKS.ALIGKEDRVIINIANEWYGT..WD
AIQ62043.1 ..SNGSPFVMRGVNHAHNWYKD....QLSTALSAIAKTGANTVRIVL.SDGK.......KWSL...DDVNTVKNIISLCEQNKLIAI.LEVH.DATGSD........SYSDLDDAVNYWIS..IKS.ALIGKEDRVIINIANEWYGT..WD
QUL52210.1 .DATGTPFVMRGVNHAHTWYKD....QLTTAIPAIAKTGANTVRIVL.SDGH.......KWTL...DDVSAVQNILSLCEQNKLIAV.LEVH.DATGSD........SLTDLDNAVNYWIA..IKS.ALIGKEDRVIINIANEWYGT..WD
QYK67373.1 .DANGNPFVMRGINHAYTWYKG....QELAAIPAIAKTGANTIRIVL.SDGQ.......QWTK...DNLSELQNLITLSEQNKLVVV.LEVH.DGTGSD........SATVLNNIANYWIE..MKG.ALIGKESTVILNIANEWYGT..WD
ADO56715.1 .DAKGNPFVMRGINHAYTWYKG....QESVAIPAIAKTGANTIRIVL.SDGQ.......QWTK...DDLSAIQNLITLSEQNKLVVV.LEVH.DGTGSD........SATVLDNITNYWIE..MKS.ALIGKESTVILNIANEWYGT..WD
AIQ31305.1 .ESNGTPFVMRGINHAYTWYKG....QDAVAIPAIARTGANTVRIVL.SDGQ.......QWSK...DNLAAIQSLITLCEQNKLVAV.LEVH.DGTGSD........SETVLNNIANYWIE..MKS.ALIGKEKTVILNIANEWYGT..WD
AUJ88464.1 .DGNGNPFVMRGVNHAYTWYKG....QESVAIPAIAKTGANTIRIVL.SDGQ.......QWTK...DDLSALQNLITLSEQNKLVVI.LEVH.DGTGND........NAAVLNKIADYWIE..MKS.ALIGKENTVILNIANEWFGT..WD
QAA30979.1 .DGNNKPFVMRGINHAYTWYKG....QESVAIPAIAKTGANTIRIVL.SDGD.......QWTK...DDLASIKNLISLCEKNKLVAI.LEVH.DGTGSD........DINRLNNAANFWIE..MKS.ALIGKEHTVILNIANEWYGT..WS
QKS47268.1 .DANGNPFVMRGINHAYTWYKG....QESVAIPAIAKTGANTVRIVL.ADGQ.......QWTK...DSLSAIQNLITLCEQNKLIAI.VEVH.DGTGSD........SQTVLDNIANYWIE..MKS.ALIGKEKTVILNIANEWYGT..WN
AAS48170.1 .DANGNPFVMRGINHGHAWYKD....QATTAIEGIANTDANTIRIVL.SDGG.......QWTK...DDIQTVRNLISLAEENNLVAV.LEVH.DATGYD........SIASLNRAVDYWIE..MRS.ALIGKEDTVIINIANEWFGS..WE
BAD99527.1 .DANGNPFVMRGINHGHAWYKD....QATTAIEGIANTGANTVRIVL.SDGG.......QWTK...DDIQTVRNLISLAEDNNLVAV.LEVH.DATGYD........SIASLNRAVDYWIE..MRS.ALIGKEDTVIINIANEWFGS..WD
AAQ31841.1 .DANGKPFVMRGINHGHSWYKP....HIETAMEAIADTGANSIRVVL.SDGQ.......QWTK...DDVDEVAKIISLAEKHSLVAA.LEVH.DALGTD........DIEPLLKTVDYWIE..IKD.ALIGKEDKVIINISNEWFGS..WS
AAT06599.1 .DANGQPFVMKGINHGHAWYKD....TASTAIPAIAEQGANTIRIVL.SDGG.......QWEK...DDIDTVREVIELAEQNKMVAV.VEVH.DATGRD........SRSDLDRAVDYWIE..MKD.ALIGKEDTVIINIANEWYGS..WD
AAN27517.1 .DANGQPFVMRGINHGHAWYKD....TASTAIPAIAEQGANTIRIVL.SDGG.......QWEK...DDIDTIREVIELAEQNKMVAV.VEVH.DATGRD........SRSDLNRAVDYWIE..MKD.ALIGKEDTVIINIANEWYGS..WD
AFC30528.1 .DANGKPFVMRGINHPHAWYKN....DLATAIPAIAATGANSVRIVL.SNGS.......QWSK...DSLAFIQNIIALCEQYRMIAI.LEVH.DATGSD........SYTALDNAVNYWIE..MKS.ALIGKERTVIINIANEWYGT..WD
AGU71466.3 .DATGKPFTMRGVNHAHSWFKE....DSAAAIPAIAATGANTVRIVL.SDGG.......QYTK...DDINTVKSLLSLAEKINLHSG.VMTH....RKD........DVESLNRAVDYWIS..LKD.TLIGKEDKVIINIANEWYGT..WD
AZK47289.1 .DSTGNPFKIRGINHAHSWFKN....DSATAMEAIAATGANTVRIVL.SNGQ.......QYAK...DDANTVSNLLSLANQHKLIAI.LEVH.DATGSD........SVSALDHAVDYWIE..MKN.VLVGKEDRVLINIANEWYGT..WD
BAM46957.1 .DANGNQFVMRGVNHAHSWYKD....TYTEAIPAIAATGANTIRIVL.SDGG.......QYQK...DDINIVRNLIETAEANNLVAV.LEVH.DATGSD........SLSDLNRAVDYWIE..IKD.ALIGKEDTVIINIVNEWYGT..WD
CBK96294.1 .DANGKEFIMRGINHAHTWYLD....EDTTAIKAIAETGSNVVRVVC.SDGE.......QWTK...DTEDMLETVIDLCIDNEMIAV.VEVH.DATGKD........DKTALDKATDYWIE..MKN.ALIGKEQYVILNIANEWTGG..WN
CBL18180.1 .DANQNAFEMRGTNYAYTWYKWE..GNEEATLKEIAAYGANTVRIVL.SDGQ.......QYTK...NTAGEVANLISLCEKYKLIAV.LEVH.DVTGKD........DANLLLNAAKYFSE..IKS.ALIGHEDTVIINIANEWQGN..TN
QGG58625.1 .DANGKPFVMRGVNYAHTWFKN....DLNTAIPAIAATGANTVRVVL.SNGS.......QYTK...DTAQNVSAILSLLKQHKMVAV.LEVH.DATGSD........SAAELDKAVNYWIE..IKN.ALIGQEKYVILNIANEWYGT..WN
APO45595.1 .DSTGKAFVMRGVNHGHSWFKN....DLNTAIPAIAKTGANTVRIVL.SNGS.......LYTK...DDLNAVKNIINVVNQNKMIAV.LEVH.DATGKD........DYNSLDAAVNYWIS..IKE.ALIGKEDRVIVNIANEWYGT..WN
AAX87003.1 .DSTGKPFYMRGINHGHSWFKN....DLNTAIPAIAKTGANTVRIVL.SNGT.......QYTK...DDLNSVKNIINVVNANKMIAV.LEVH.DATGKD........DFNSLDAAVNYWIS..IKE.ALIGKEDRVIVNIANEWYGT..WN
QBF51372.1 .DSTGKPFYIRGINHGHSWFKN....DTATAIPAIAKTGANTVRIVL.SNGT.......QYTK...DDLNSVKNIINLAEENKIIAV.LEVH.DATGKD........DFNSLDAAVNYWIS..IKE.ALIGKEDRVIVNIANEWYGT..WN
AEX60762.1 .DSTGKPFVMRGVNHSHTWFKN....DLNAAIPAIAKTGANTVRIVL.SNGV.......QYTR...DDVNSVKNIISLVNQNKMIAV.LEVH.DATGKD........DYASLDAAVNYWIS..IKD.ALIGKEDRVIVNIANEWYGT..WN
ANF96694.1 .DSTGKPFAMRGINHAHTWYKN....DLSTAIPAIARTGANTVRIVL.SNGM.......QYTK...DDVNSVKNIISLVNQNKMIAV.LEVH.DATGKD........DYNSLDAAVNYWIS..IKD.ALIGKEDRVIVNIANEWYGT..WN
AAX87002.1 .DSTGKAFVMRGVNHPHTWYKN....DLNAAIPAIAQTGANTVRVVL.SNGS.......QWTK...DDLNSVNSIISLVSQHQMIAV.LEVH.DATGKD.E.......YASLEAAVDYWIS..IKG.ALIGKEDRVIVNIANEWYGN..WN
ADM68451.1 .DSTGKPFVMRGVNHAHTWYKN....DLYTAIPAIAQTGANTVRIVL.SNGN.......QYTK...DDINSVKNIISLVSNYKMIAV.LEVH.DATGKD........DYASLDAAVNYWIS..IKD.ALIGKEDRVIVNIANEWYGS..WN
ANY76761.1 .DSTGKAFVMRGMNHAHTWYKN....DLHAAIPAIANTGANSVRIVL.SNGT.......QWTR...DDVDAVKSIISLVNDNNMIAV.LEVH.DATGKD........DHASLDAAVNYWID..IKE.AIIGKEDRVIVNIANEWHGS..WS
CQR54354.1 .DATGSPFVMRGVNHAHTWYKN....DLAAAIPAIAATGANTVRIVL.SNGG.......QWSL...DSLSDVQNILALCDQYKLTAM.LEVH.DATGSD........NASTLNAAVNYWIS..IKD.ALIGKEDRVIVNIANEWFGS..WS
AIQ19732.1 .DATNSPFVMRGINHAHTWYKN....DLETAIPAIAATGSNVVRIVL.SNGN.......KWSL...DSVADVQHILALCDEYKLTAM.LEVH.DATGSD........NISDLNAAVNYWIS..IKD.ALIGKEDRVIVNIANEWFGS..WS
AIQ75360.1 .DATGSPFVMRGVNHAHTWYKN....DLATAIPAIAATGSNTVRIVL.SNGS.......KWSL...DTLADVQNILKLCDQYKMIAM.LEVH.DATGSD........NTSDLNAAVNYWIS..IKD.ALIGKEDRVIVNIANEWYGS..WN
AWV34670.1 .DATGSPFVMRGVNHAHTWYKN....DLATAIPAIAATGSNTVRIVL.SNGS.......KWSL...DTLADVQNILKLCDQYKMIAM.LEVH.DATGSD........NTSDLNAAVNYWIS..IKD.ALIGKEDRVIVNIANEWYGS..WN
QMV43160.1 .EANGTPFVMRGVNHAHTWYKN....DLAAAIPAIKATGANTVRIVL.SNGG.......QWTR...DDIASVQNILALCNQYKLIAM.LEVH.DATGSD........STSTLQAAVDYWIS..IKD.ALIGKEDRVIINIANEWYGT..WD
ACU52526.1 .DANGQEFMIRGVSHAHTWYKD....DINGAITSIAAAGANTVRIVL.SNGG.......QWTK...DNLDSVQNILSLCESHKLIAM.LEVH.DATGND........SQETLENAVNYWKE..LRD.LLIGKEDRVIINIANEWFGT..WD
AAT39478.1 .DKNGDPYVMRGVNHGHSWFKQ....DLEEAIPAIAETGANTVRMVL.SNGQ.......QWEK...DDASELARVLAATETYGLTTV.LEVH.DATGSD........DPADLEKAVDYWIE..MAD.VLKGTEDRVIINVANEWYGS..WR
AST98531.1 .DKNGDPYVMRGVNHGHSWFKQ....DLEEAIPAIAETGANTVRIVL.SNGQ.......QWEK...DDASELARVLAATETYGLTTV.LEVH.DATGSD........NPDDLDKAVDYWIE..MAD.VLKGTEDRVIINIANEWYGA..WR
AAQ31839.1 .DANGNPFVMRGINHGHAWFKQ....ELETSMRGISQTGANTIRVVL.SNGQ.......RWQK...DDRNMVASVISLAEQHQMIAV.LEVH.DATGSN........NFSDLQAAVDYWIE..MKD.VLQGKEDIVIINIANEWYGA..WD
CDM68969.1 .DANGKEFVMRGINHAHAWYKG....NEETAISAIAKTGANTVRIAV.GDGE.......QWGY...DDINTLKNLISLCEKNKLVAV.VEVH.DATGSD........DISKLNNAVSYWVK..MKD.ALIGKEDTVILNIANEWFGT..WD
QUI25581.1 .DANGQAFVMRGINHAHTWWKG....NEETAIKAIADLGANTVRIVL.SNGD.......RWSY...DDIDTVKNLLSICEKNKLIAV.LEVH.DATGSD........DYTKLANAVDYFIA..MKD.ALVGKEDRVIINIANEWFGT..WD
ALP89769.1 .DANGKEFVMRGINHAHAWYKG....QESTAIPAIAKTGANTVRVAL.GDGD.......QWGY...DDINTVKNIISLCEKNNLVAV.LEVH.DATGSD........DYSKLDNAVNYWIS..MKD.ALKGKESTVILNIVNEWYGT..WD
BBG30007.1 .DANGQSFLIRGINHAHTWYKA....DIEAALKSIAATGANLVRIVL.SNGT.......QWEK...DDVESVRNILSLCERHQLIAM.LEVH.DATGSD........NLQALQHAISYWIE..LSA.VLIGKEDRVIINIANEWFGS..WS
QGW99981.1 .DAKGNPFIMRGINYPYAWYKG....QEDTAIPAIARTGANCIRIVM.GDGQ.......QYSK...TSLAEIQKLLQLCKDNHLIAI.VEAH.DATGSD........NIQDLENAANYWME..MKD.ALIGNEDHVILNIANEWGGA..WD
AQP41170.1 .DAKGNPFVMRGINHAYSWYQG....YEDTAIPAIAKTGANCVRLVI.TDGQ.......QYSK...TSLSELQKLIQLCKDNKLIAI.VEVH.DATGSD........NIQDLEKAADYWIE..MKS.ALIGNEANVILNIANEWGGA..WD
QGX44134.1 .DAKGNPFIMRGINHAYSWYPG....YEDTAIPAIARTGANCVRLVI.TDGQ.......QYNK...TSLSEIQKLIQLCKDNKLIAI.VEVH.DATGSD........NIQDLQKAADFWIE..MKS.ALIGNEANVILNIANEWGGA..WD
ATC23771.1 .EASGRPFVMRGVNVHHAWAPE....RTPASLAAIAATRANVVRIVL.SDGSRVLGDGARWRR...NTPREVEGILDQAERLKLVVI.LEVH.DATGFGDPSPDARGATHLGGVIPYWLS..LKP.VLEGREDRVIINIANEPFSVR.DG
AYV49609.1 VDACGEAFVARGVNLPHAWKPR....ATGKAIRQSAALGANAVRIVL.SDGT.......RWRR...TSPQAVRKILVQARRARQVVI.LEVH.DTTGYGLEPE....AAHIGSALAYWAK..LEP.VLRGQEDLVLVNIGNEPTAGA.TT
BCJ57634.1 .DANGNEFIMRGVNHAHTWYPQ.....QTSSFANVKALGANTVRVVL.SSGD.......RWTR...NSAADVTNVIQLCKTNRLICV.LEVH.DTTGYG.EQS...GAITLDRAVDYWLS..IAS.ALNGQEAYVIVNIGNEPYGN..QN
QGN49881.1 .DANGNEFIMRGVNHAHTWYTH.....QTRSFADIKALGANTVRVVL.SSGD.......RWTR...NSATDVANVISLCRTNRLICV.LEVH.DTTGYG.EQS...GAITLDRAVDYWLS..IAD.VLRGQEAYVIVNIGNEPYGN..QN
SCG72161.1 .DANGTEFVMRGVNHAHTWYPQ.....QTSSFANIKALGANTVRVVL.ASGD.......RWTK...NSAADVGNVISLCKANRLICV.LEVH.DTTGYG.EQS...GAITLDRAVDYWLS..IAS.ALQGQEKYVIVNIGNEPYGN..QG
ADJ49127.1 .EANGSPFVMRGVNHAYVWYPG.....QNRAFADIKSFGANTVRVVL.GSGQ.......RWGP...TSAAEVTSVIGQCKQNRLICV.LEVH.DTTGYG.EQS...GAATLDQAAGYWIS..VAD.ALKGQENYVVINLGNEPFGNDQQV
ANN17163.1 .EANGAAFVMRGVNHAHVWYPN.....QTGSFANMKSFGANTVRVVL.GSGQ.......RWGP...SSSANVRNVISLCKQSRQICV.LEVH.DTTGYG.DQS...GAATLDQAASYWLS..VAD.ELKGQENHVVINLGNEPFGNDQQV
ASW54425.1 .DANGNAFVMRGVNHAHTWYPQ.....QTGSFADIASLGANTVRVVL.SSGD.......RWTQ...NSPSDVANVISLCKANRLICV.LEVH.DTTGYG.EEG...AAITLDRAVDYWLT..LKS.VLTGQEAYTIINIGNEPYGN..QG
ATO14192.1 .DANGTEFVMRGVNHAHTWYPQ.....QTSSFANIKALGANTVRVVL.GSGD.......RWTK...NSAADVANVISLCKTNRLICV.LEVH.DTTGYG.EDG...AATTLARATDYWLS..IAD.VLKGQEKYVIVNIGNEPFGN..QG
AXO36964.1 .DANGSEFVMRGVNHAHTWYPQ.....QTSSFANIKALGANTVRVVL.ASGD.......RWTK...NSAADVANVISLCKTNRLICV.LEVH.DTTGYG.EEG...AAATLAKATDYWLS..IAD.VLKGQEKYVIVNIGNEPFGN..QG
QDY07284.1 .DANGNEFIMRGVNHAHTWYPQ.....QTTSFANIKALGANTVRVVL.ASGD.......RWTK...NTAADVSNVISLCKANKLICV.LEVH.DTTGYG.EQA...GAITLDRAVDYWLS..IAS.ALQGQERYVIVNIGNEPYGN..QG
QLD24471.1 .DANGNEFVMRGVNHAHTWYPQ.....QTSSFANIKALGANTVRVVL.ASGD.......RWAK...NDTADVANVISLCKANKLICV.LEVH.DTTGYG.EQS...GAITLDRAVDYWLS..ISS.ALAGQEKYVIVNIGNEPYGN..QG
SBT37134.1 .DANGTEFVMRGVNHAHTWYPQ.....QTSSFANIKALGANTVRVVL.ATGD.......RWAK...NDTADVANVISLCKANKLVCV.LEAH.DTTGYG.EDG...AATTLDRAASYWLS..VAS.ALQGQEKYVIINIGNEPFGN..QG
SBT38821.1 .DANGTEFIMRGVNHAHTWYPQ.....QTSSFANIKALGANSVRVVL.ASGD.......RWAK...NDTADVANVISLCKANRLICV.LEVH.DTTGYG.EQS...GAITLDRAVTYWLS..IAS.ALQGQEKYVIVNIGNEPYGN..QG
SCE69983.1 .DANGAEFVMRGVNHAHTWYTH.....QTSSFANIKALGANTVRVVL.SSGQ.......RWTK...NETADVANVISLCKQNRLICV.LEVH.DTTGYG.EQA...GAATLAQAVDYWIS..VQS.ALTGQEKYVIVNIGNEPYGN..QN
SCE72877.1 .DANGTEFIMRGVNHAHTWYPQ.....QTSSFADVKALGANTVRVVL.SSGD.......RWTR...NTNADVANVISLCKANRLICV.LEVH.DTTGYG.EQS...GAITLARAVDYWLS..LAD.VLAGQEKYVIVNIGNEPYGN..QN
SCF27982.1 .DANGTEFVMRGVNHAHTWYPQ.....QTSSFADVKTLGANTVRVVL.SSGD.......RWTR...NTNADVANVISLCKANRLICV.LEVH.DTTGYG.EQD...GAITLARAVDYWLS..LAD.VLVGQERYVIVNIGNEPYGN..YN
SCF36577.1 .DAGGTEFVMRGVNHAHTWYPQ.....QTSSFANVKALGANSVRVVL.ASGD.......RWAK...NSAADVSNVISLCKANRLICV.LEVH.DTTGYG.EQS...GAISLDRAVDYWLS..IAD.ALNGQERYVIVNIGNEPYGN..QG
SCG17558.1 .DANGSEFVMRGVNHAHTWYQQ.....QTSSFANIKALGANTVRVVL.ASGQ.......RWAK...NDTADVANVISLCKANRLICV.LEVH.DTTGYG.EQS...GAATLDQAADYWLS..VSS.ALAGQEKYVIVNIGNEPYGN..QG
SCG50027.1 .DANGNEFVMRGVNHAHTWYPQ.....YTNSFANIKALGANTVRVVL.ATGD.......RWAK...NDTADVANVISLCKQNKLICV.LEAH.DTTGYG.EDG...AATTLDRAANYWLS..IAS.ALQGQEKYVIVNIGNEPFGN..QN
SCG65882.1 .DANGAEFVMRGVNHAHTWYPQ.....QTSSFADVKALGANSVRVVL.ASGQ.......RWTR...NTNADVANVISLCKANRLICV.LEVH.DTTGYG.EQS...GAASLDQAVDYWIS..LAD.VLTGEEEYLIVNIGNEPYGN..QG
AEB43751.1 .DANGNEFIMRGVNHAHTWYTH.....QTSSFANIKALGANTVRVVL.SSGD.......RWTR...NSADDVRNVIELCKANRLICV.LEVH.DTTGYG.EQS...GAVSLDRAVDYWLS..IAS.ALNGQERFVIVNIGNEPYGN..QN
AUS77477.1 .DANGNEFIMRGASHPHAWFTH.....ETGAFADIKALGANTVRVVL.SSGH.......RWER...SDVDDVANVVSLCKANRLICV.LEVH.DTTGYG.EQD...GAASLDQAVDYWLD..VRE.AVEGEEAYVIVNIGNEPHGN..TG
QKW13015.1 .DANGNEFVMRGVNHAHTWYAN.....QTSSFANIKALGANTVRVVL.SSGD.......RWTR...NSADDVSNVIQLCKTNRLICV.LEVH.DTTGYG.EQS...GAATLDQAVDYWLS..IAG.ALNGQERYVIVNIGNEPYGN..QN
AOS63253.1 .ESNGSEFVMRGVNHPHSWFTN....ETPQSLADIKSLGANTVRVVL.SSGD.......QWTE...NDAADVAEVIGQCKANRLICV.LEVH.DTTGYG.EQP...TAVSLDRAVDYWLR..IQS.ALVGQEDYVLVNIGNEPFGN..NN
APU14662.1 .DANGQEFVIRGVSHPHTWFTS....QTTQSLADIKSLGANTVRIVL.SSGD.......QWTE...NSAADVANVVDQCKANRLICM.LEVH.DTTGYG.EQA...AAVSLDRAVDYWLR..IQD.AVIGEEDYVLVNIGNEPYGN..TD
ANZ40233.1 .DATGAEFVMRGVNHAHTWYTS....RTTQALTDLKALRSNTVRVVL.SSGD.......RWTR...NDAADVANVISQCKRNRLICV.LEVH.DTTGYG.EQS...GAVSLARAVDYWIS..VQS.AMTGQEAYVILNIGNEPWGN..TG
ATE53439.1 .DANGNDFVLRGVNHAHTWYPT....RTRQALADVKALGANSVRVVL.ASGD.......RWTR...NDAADVADVIAQCKANRLICV.LEVH.DTTGYG.EQS...GAIPLSKAVDYWVG..VKS.ALVGQEKYAILNIGNEPHGN..TG
AXB42212.1 .ESNGAEFVMRGVNHAHTWYRG....ETS.SFANIKALGANTVRVVL.SSGH.......QWTK...NDAADVSTVVSLCKQNRLICV.LEVH.DTTGYG.EQA...AAVSLDTAANYWLE..VKN.SIIGQEQYVIVNIGNEPWGN..TG
AXG81675.1 .EANGNEFVMRGVNHAHTWYTG....RTTAALSDIKALGANTVRVVL.STGD.......QWTR...NDTADVANIVAQCKQNKLICV.LEAH.DTTGYG.EQS...AAVTLSRAADYWIS..VQS.ALTGQESYVIVNIGNEPYGN..TN
QLH19869.1 .EGNGSTFVMRGVNHAHTWYAT....RTTQTLSDIKALGANTVRVVL.STGD.......RWTK...NDTADVANVVAQCKRNRLICV.LEAH.DTTGYG.EQG...GAVTLSRATDYWIS..VRT.ALAGQESYVVVNLGNEPYGN..TN
```

Continued from previous page

```
BAM62868.1 .EASGNDFVMRGVNHAHTWYAD.....RLDALDHIKAKGANTVRVVL.SSGD.......RWTR...NDTADVANVVAQCKRNRLICV.LEVH.DTTGYG.EQS...GAVTLSRAADYWIE..VRD.ALAGQEDYVIVNIGNEPHGN..TD
AXI85415.1 .EASGNDFVMRGVNHAHTWYAD.....RLDSLDHIKAKGANTVRVVL.SSGD.......RWTR...NNTADVANVVTQCKRNRLICV.LEVH.DTTGYG.EQS...GAVTLSRAADYWIE..VRD.ALVGQEDHVVVNIGNEPHGN..TG
QYX82013.1 .ERSGNDFVMRGVNHAHTWYPN.....QIGALAHIKAKGANTVRVVL.SSGD.......RWTR...NDTADVANVVAQCKRNRLICV.LEVH.DTTGYG.EQS...GAVTLARAADYWIG..VRS.ALTGQEDHVIVNLGNEPYGN..ND
AYL34687.1 .EASGNDFVMRGVNHAHTWYAD.....RISSLAHIKAKGANTVRVVL.SSGD.......RWTR...NDTADVANVVAQCKQNRLICV.LEVH.DTTGYG.EQS...GAVTLSRAADYWIG..VQS.ALAGQEDHVIVNIGNEPHGN..TG
AJP00836.1 .EASGNDFVMRGVNHAHTWYPD.....RISSLAHIKAKGANTVRVVL.SSGD.......RWTR...NDTADVANVVGQCKQNRLICV.LEVH.DTTGYG.EQS...GAVTLSRAADYWIS..VQS.ALTGQEDYVIVNIGNEPYGN..TN
AKH86353.1 .EGNGNDFVLRGVNHPHAWYTG.....ELDSLREIKQLGANSVRVVL.ATGD.......RWPR...SDTADVDNVVSQCKANRLICV.LEVH.DTTGYG.EED...AAVSLDRAADYWIS..VRD.ALVGEEDYVIVNLGNEPHGN..QG
AMW08869.1 .EAGGNDFVMRGVNHAHTWFPD.....RISSLAHIKAKGANTVRVVL.SNGD.......RWTR...NDAADVTNVISQCKQNRLICV.LEVH.DTTGYG.EQS...GATTLSRAADYWIS..VQS.ALAGQEDYVIVNIGNEPYGN..TN
AXI79531.1 .EGNGNDYIMRGVSHAHTWYPD.....KLSSLADIKKLGANTVRVVL.ATGD.......LWTK...NSATDVANVVAQCKANRLICV.LEAH.DTTGYG.EQS...GAITLSKAADYWIS..IQS.ALTGQENYIIINIGNEPYGN..NN
BCJ73223.1 .EANGTALKLRGINHPHAWYAT.....QTSSFANIKAAGANSVRVVL.SGG........RWTT...NTAADVANVISLCKTNKLICV.LENH.DTTGFG.EQS...GAVSLDAAVNYWIS..IQS.ALTGQENYVILNIGNEPIGNN.AV
CBG75158.1 .ERSGNDFVMRGVNHAHTWYAD.....QIGSLAHIKAKGANTVRVVL.SSGD.......RWTR...NDTADVANVVAQCKRNRLICV.LEVH.DTTGYG.EQS...GAVTLSRAADYWIG..VQS.ALTGQEDYVVVNIGNEPYGN..NN
QDI72496.1 .EASGNDFVMRGVNHAHTWYAD.....RIDSLAHIKAKGANTVRVVL.SNGD.......RWTR...NDASDVANVVAQCKQNRLICV.LEVH.DTTGYG.EQS...GATTLSRAADYWIS..VRS.ALAGQEDYVIVNIGNEPYGN..TN
QEU84399.1 .EASGNDFVMRGVNHAHTWYPD.....RIDALAHIKAKGANTVRVVL.SSGD.......RWTR...NDASDVANVVARCKQNRLICV.LEVH.DTTGYG.EQS...GAVTLSRAADYWIS..VQS.ALTGQENHVIVNIGNEPHGN..SG
QEV05093.1 .EASGNDFVMRGVNHAHTWYAD.....RIGSLAHIKAKGANTVRVVL.SSGD.......RWTA...NDAADVANVVAQCKQNRLICV.LEVH.DTTGYG.EQS...GAVSLSRAADYWIG..VRS.ALTGQEDHVIVNIGNEPHGN..TG
QEV23521.1 .EASGNDFVMRGVNHAHTWFPD.....RISSLSHIKAKGANTVRVVL.SSGD.......RWTR...NDTADVANVVAQCKQNRLICV.LEVH.DTTGYG.EQS...GAVTLSRAADYWIS..VQS.ALTGQEDYVIVNIGNEPHGN..TG
QEV72209.1 .ESNGNDFVMRGVNHAHTWYPD.....RASSLAHIKAKGANTVRVVL.SSGD.......RWTR...NDAADVSNVVAQCKQNRLICV.LEVH.DTTGYG.EQS...GAVTLSRAADYWIG..VQS.ALTGQEDYVIVNIGNEPYGN..TN
QOV33815.1 .ESNGNDFVMRGVNHAHTWFPD.....RVSSLAHIKAKGANTVRVVL.SSGD.......RWTR...NDTADVANVVAQCKQNRLICV.LEVH.DTTGYG.EQS...GAATLSRAADYWIS..VQS.ALAGQEDYVLVNIGNEPYGN..TG
AGZ42057.1 .DANGNNFVMRGVNHAHTWYAD.....KLGSLADIKALGANTVRVVL.SSGD.......RWTR...NDAANVTTVINTCKANKLICV.LEVH.DTTGYG.EDS...AATTLAKAVDYWIS..IKS.ALAGQERYVILNIGNEPYGN..TN
AGL18898.1 VDANGNPFVMRGVSHAHVWYQS....RTQQALADIKALGANSVRIVL.GSGQ.......RWGP...TPAAEVTTVINQCKANRLICV.LEAH.DTTGYG.EQA...GAATLDQAVDYWIS..VKS.ALAGQERYVILNIGNEPYGN..TN
BAL89262.1 VDANGKPFVMRGVNHAHTWYPS....QLNSSLSGIKALGANTVRVVL.ASGQ.......RWTK...NEASDVTNVISQCKANRLICV.LEVH.DTTGYG.EQS...GAATLAQAVDYWIS..VKS.ALEGQESYVILNIGNEPYGNG.SA
BBH69472.1 .DANGNTFVMRGVNHAHTWYAS....QLGSSLAGAKALGSNTVRVVL.ASGQ.......RWTK...NEASDVANVISQCKANRLICV.LEVH.DTTGYG.EQA...GAATLAQAVDYWIS..VKS.ALDGQESYVILNIGNEPYGNG.SA
BCB91458.1 .EANGTNVVMRGVSHPHVWYTG.....QTSSFANIKALGANTVRVVL.GSGQ.......RWG.....PSNDLSTVVNLCRTNRLLCV.LEVH.DTTGYG.EEG...AAATLDQAASYWIG..QRS.VLAGTENFIVINIGNEPFGNNASV
SDT52805.1 .EGNGAPFVMRGVNHAHTWYAS.....QLSSLANSKALGANTVRVVL.SSGN.......RWTR...NDAADVSNVISQCKANRLICV.LEVH.DTTGYG.EEA...AAATLAQAVDYWIS..IKS.ALDGQENYVILNIGNEPYGN..TN
AXE83855.1 .EANGNDFVMRGVNHAHTWYPS.....RISSIAHIKAKRANTVRVVL.ANGD.......RWAR...NDTADVANVVSQCKRNRLICV.LEVH.DTTGYG.DQS...GATTLSRAADYWIS..VKS.ALTGQESYVIVNIGNEPIGN..NN
QNP74875.1 .EASGNDFVMRGVNHAHTWYRD.....RTSSLAHIKATGANTVRVVL.ASGD.......RWTR...NDTTDVANVVSQCKRNRLICV.LEVH.DTTGYG.DQS...GATTLSRAADYWIS..VKS.ALAGQESYVIVNIGNEPYGN..SN
QZZ25189.1 .EANGNDFVMRGVNHAHTWYPG.....QISSIAAIKAKRANTVRVVL.ANGD.......RWAR...NSAADVANVVTQCKRSRLICV.LEVH.DTTGYG.DQS...GATTLARAADYWVS..LKS.TLAGQESYVVINIGNEPIGN..LN
ACZ83938.1 .EAGGATFVMRGTSHAHTWYPT.....QTASFAGIKSLGANTVRVVL.SGG........RWTA...NGAADVANVVSLCKQNKLICV.LENH.DTTGYG.EQS...GAYTLDQAVDYWIS..VKS.ALAGQEDYVVLNIGNEPFGNN.AT
ADG87649.1 .EANGAEFIMRGTSHPHAWYPN.....QTGSFADIKSLGANTVRVVL.SGG........RWTP...NSPSDVANVIRLCKENRLICV.LENH.DTTGYG.EQS...GAYPLDQAVDYWIS..LKS.VLTGEENYIVINIGNEPYGNN.NP
ANZ41596.1 .EGNGTPFVMRGINHAHVWYQN.....QTRAFADMKSFGTNTVRVVLGSGQ.......RWGP...TPASEVSSVIALCKQNRMICV.LEVH.DTTGYG.EQS...GAASLDQAATYWIG..VAD.ALKGQEDYVVINLGNEPFGNNAEV
AQZ68439.1 .EANGNAFVMRGVSHAHTWYTS.....QTSSFANIKALRANTVRVVL.SGG........RWTP...NNATDVANVVSLCRQNRLICV.LENH.DTTGYG.EQS...GAYTLDQAVDYWSS..VRS.ALTGTEDFIIVNIGNEPYGNN.NV
AWS40398.1 .ESSGSAFVMRGTSHAHTWFPT.....QTGSFAAIKALGANTVRVVL.SGG........RWAA...NGVADVTNVVSLCKQNKLICV.LENH.DTTGYG.EQS...GAYTLDQAVDYWIG..VKS.ALVGQEDYVVLNVGNEPFGNN.AT
QFY13598.1 .EADGQEFVMRGTSHAHTWYAT.....QTKAFADIKSLKANTVRVVL.SGG........RWTA...NSVSDVANVVSLCKQNKLICV.LENH.DTTGYG.EQS...GAYTLDQAADYWIS..VKS.ALVGQEDYVIVNIGNEPVGNN.AV
QYC45281.1 .EANGNAFVMRGVSHAHTWYTS.....QTSAFADIKALKANTVRVVL.SGG........RWTP...NSASDVANVVSLCRQNRLICV.LENH.DTTGYG.EQS...GAYTLDQAVTYWNS..VKS.ALTGTEDFIIINIGNEPYGNN.NV
BCJ67540.1 .ESNGNEFIMRGVSHPHAWYTH.....QTSSFANIKSLGANTVRVVL.SGG........RWAA...NGPADVANVVALCKQNKLICV.LENH.DTTGYG.EQA...GAVTLDRAVDYWIS..LRD.VLAGQEKYIIVNIGNEPIGNN.PV
QFZ18119.1 .DANGNDFVMRGVNHAHTWYAD....RTTQALKDIKATGANTVRVVL.SSGD.......RWTR...NDAADVANVISQCKANRLICV.LEVH.DTTGYQ.EQS...GAVSLDRAVDYWVA..VKG.AMAGQEKYVILNIGNEPWGNT.GY
UAB98931.1 .DANGNEFVMRGVSHAHTWYPS.....QTGAFANIKALGANTVRVVL.ATGD.......QWAK...NDAADVANVVSLCKANRLICV.LEAH.DTTGYG.EAG...AAVSLDKAVDYWLS..IKS.ALVGQEKYVIVNIGNEPYGNNAAT
AVT37994.1 .DARGNEFIMRGVSHAHTWYAS.....QTGSFRDIKSLGANTVRVVL.SSGA.......RWTQ...NSASDVANVISLCKANRLICV.LEVH.DTTGYG.EEG...AAITLDQAVSYWIS..IQS.ALAGQENYVILNIGNEPFGNNEAT
QQQ79201.1 .DANGSEFVMRGVNHAHAWYPD....RTAQALKDVKALGANSVRVVL.STGA.......RWTW...TSTSAVSSIVSQCKADKLVCV.LEAH.DTTGYG.EQG...GAVTLNQAVDYWLG..VKS.ALVGQEKYVIINVGNEPYGN..NN
CCA60180.1 VDATGKDFVLRGVNHAHTWYTD....RTGQSLADIKALGANSVRVVL.STGT.......RWTR...NDASDVAAVVAQCKANRLVCV.LEAH.DTTGYG.EQS...DAASLSEAVDYWLE..VAS.ALKGQEKYVIVNLGNEPHGN..AG
QFZ19812.1 .DANGNEFVMRGVNHAHAWYPD....RTAQALPDIKALGANTVRVVL.ATGA.......RWTR...TSPSAVSTIVSQCRANRLICV.LEAH.DTTGYG.EQA...GAATLGQAVDYWLG..VKS.ALVGQERYVIINIGNEPYGN..GN
QNP65869.1 .EANGNEFVMRGVNHAHTWYPD.....RTGSIADIAAKGANTVRVVL.SSGD.......RWTR...TSASEVSSLIAQCKASKVICV.LEVH.DTTGYG.EDG...AAASLDRAADYWIG..VKS.ALQGQESYVVVNIGNEPFGN..TG
ADK91085.1 .EANGNEFVMRGVNHAHAWYPN.....RTGSIAHIKAKGANTVRVVL.ANGD.......RWTR...TSASEVSSIIGQCKQNRLICV.LEVH.DTTGYG.EDG...AATSLSRAADYWIG..VKS.ALEGQENYVVINIGNEPFGN..NG
AKZ57916.1 .EADGSDFVMRGVNHAHAWYPD.....RTGSLADIAAEGANTVRVVL.SSGD.......RWTR...TSAAEVSSVIGRCKAAKVICV.LEVH.DTTGYG.EDG...AATSLDRAANYWIG..VRS.ALEGQEDYVVVNIGNEPFGN..TG
BBB03672.1 .EANGNQFVMRGVNHAYAWYPG.....QTTAIADIASRGANAVRVVL.SNGD.......RWTR...TTTAQVSALIAQCKAAKVICV.LEVH.DTTGYG.EDA...AATTLDRAAQFWVD..NKS.ALDGQESHVVVNIANEPWGN..SG
CCA60191.1 .EANGSEFVMRGVNHAHTWYPS.....ETGAIADIAATGANTVRVVL.SSGD.......RWTE...TTSAQVSSIISDCKANKVICV.LEVH.DTTGYG.EDG...AAATLDQAADYWVG..VKS.ALQGQEDYVVVNIGNEPYGN..SG
QEU70798.1 .EGNGNDFVMRGVNHAHTWYPE.....RTGALADIKEKGANTVRVVL.SSGD.......RWTR...TSASEVSGIVAECRKNRLICV.LEVH.DTTGYG.EDG...AAATLDEAVDYWTG..IRS.VLEGQENHVVINLGNEPYGN..TG
QGZ52610.1 .ESNGNDFVMRGVNHPHAWYTD.....RTGSLAHIKAKDANTVRVVL.SSGQ.......QWPM...TSLAEVSKIVGLCKQNRLICV.LEVH.DTTGYG.EVS...GAATLSEAADYWIK..VKG.ALQGQEKYVVINIGNEPYGN..KD
QKW20412.1 .ESNGNRFVVRGVNHAYAWYPS.....QATAIADIAAKGANTVRVVL.GDGQ.......RWTR...TSTAQISALIAQCKAAKVICV.LEVH.DATGYG.EDA...AAGTLDGAAQFWVD..NKS.ALIGQENHVVVNIANEPWGN..TG
QLJ02980.1 .EANGNEFVMRGVNHAYDWYPS.....RTSAIADIAAKGANTVRLVL.GSGH.......RYTR...TSGSEVASLIAQCKQNKVICV.LEVH.DTTGYG.EDS...AAASLDEAADYWVG..VKD.ALAGQENYVVVDIGNEPYGN..TG
QCB25360.1 .EGNGNAFVMRGVNHAYTWYPE.....QTSSIADIAAKGANTVRVVL.GSGG.......RWTK...TNASQVSTLIEQCKANKVICV.LEVH.DTTGYG.EDG...AATSLDQAADYWIS..VKS.ALEGQEDYVVVNIGNEPFGN..TN
AAA26710.2 .EGNGSAFVMRGVNHAYTWYPD.....RTGSIADIAAKGANTVRVVL.SSGG.......RWTK...TSASEVSALIGQCKANKVICV.LEVH.DTTGYG.KDG....ATSLDQAGDYWVG..VKSAAWRAQEDYVVVNIGNEPFGN..TN
AIJ12409.1 .EGNGSAFVMRGVNHAYTWYPD.....RTGSIADIAAKGANTVRVVL.SSGG.......RWTK...TSASEVSALIGQCKANKVICV.LEVH.DTTGYG.EDGA...ATSLDQAADYWVG..VKS.ALEGQEDYVVVNIGNEPFGN..TN
ABY90130.1 .EANGNEFVMRGANHAYAWYPQ.....QTTSIADIAAKGANTVRVVL.GSGD.......RWTE...TSASQVSTIIGQCKANKVICV.LEVH.DTTGYG.EDG...AAATLDQAADYWIG..VKS.ALEGQEDYVVVNIGNEPFGN..SN
APY84783.1 .EGNGNPFVMRGVNHAYTWYPE.....RTRSIADIAAKGANTVRVVL.SSGG.......RWTK...TSASQVSALIDQCKKNKVICV.LEVH.DTTGYG.EDG...AATSLDQAADYWVG..VKS.ALEGQEDYVIVNIGNEPFGN..TN
QNQ32617.1 .EANGNKFVMRGINHAYTWYPE.....QTTAIADIAAKGANTVRVVL.GSGD.......RWTK...TSASQVSGLIGQCKANKVICV.LEVH.DTTGYG.EDG...AATTLDKAADYWIS..VKS.ALEGQEDYVVVNIGNEPYGN..TN
QIS73744.1 .DANGEDFVLRGVNHAHAWYPT....RTARALKDIKALGANSARVVL.ATGD.......RWTK...NDTADVAAVVAQCKQNRLVCV.LEAH.DTTGYG.EQA...GATTLARAVDYWIG..VKN.AVQGQEKYVILNIGNEPYGN..SG
ADK91084.1 .DANGEDFVLRGVNHAHAWYPA....RTPQALKDIKALGANSVRVVL.ATGD.......RWTK...TGTADVASIVAQCRQNRLVCV.LEAH.DTTGYG.EQA...GAVPLSRAVDYWVS..VAD.ALRGQEKYVVVNLGNEPHGN..SG
AKN71965.1 VDAGGTDFVLRGVNHAHAWYPT....RTPQALKDIKALGANSVRVVL.ATGD.......RWAR...NDTADVAAVVSQCKQNRLVCV.LEAH.DTTGYG.EQD...GAVPLSRAVDYWIS..VED.AVKGQEKYVILNIGNEPYGNGSSG
AYN37844.1 .DATGKDFVLRGVNHAHAWYPT....RTAQALKDVKALGANSVRVVL.ATGD.......RWTK...NDTADVASVVAQCKENRLVCV.LEAH.DTTGYG.EQD...GAVSLSRAVAYWTS..VKA.AVQGQEKYVVLNIGNEPYGN..TG
CQR60862.1 VDASGKDVVLRGVNHAHAWYPT....RTAQALKDIKALGANSVRVVL.ATGD.......RWAR...NDTADVAAVVAQCKQNRLVCV.LEAH.DTTGYG.EQD...GAVPLSRAVDYWVS..VKD.AVKGQEKYVILNIGNEPYGNGDSG
QIJ65213.1 .DATGRDFVLRGVNHAHTWYPA....RTPQALKDIKALGANSVRVVL.ATGD.......RWAK...NDTADVAGVVAQCKQNRLVCV.LEAH.DTTGYG.EQA...GAVSLSRAVDYWIS..VKD.AVRGQEKYVILNIGNEPHGNGTSG
ALC18858.1 .DAGGNDFVLRGVNHAHTWYPT....RTTQALKDIKALGANSVRVVL.STGD.......QWTR...NDAADVADIVAQCKANRLVCV.LEAH.DTTGYG.EAG...AAVPLSRAVDYWID..VQA.ALKGQEKYVILNIGNEPYGN..SG
QNE73622.1 .DANGNDFVLRGVNHAHAWYPG....RTERALADVKALGANSVRVVL.STGD.......RWTK...NGAADVKAVVAQCRANRLICV.LEAH.DTTGYG.EQD...GAVSLDRAVDYWVD..VQE.AVKGQEKYVILNLGNEPHGN..SG
QQQ78619.1 .DANGNDFVMRGVNHAHTWYAD....RTTQALKDIKATGANTVRVVL.SSGD.......RWTL...NTASDVAGVISQCKANKLICV.LEVH.DTTGYQ.EQS...GAVTLARAVEYWKS..IQS.ALAGQEKYVILNIGNEPWGN..TG
AIS01618.1 .DAGGKDFVMRGVNHAHTWYPA....RTDQALKDIKAKGANTVRVVL.STGD.......QWTR...NGAADVAHVVAECKENRLICV.LEAH.DTTGYG.EAG...AAVPLGKAVDYWLG..VKD.ALVGQEKYVIVNIGNEPYGN..TN
AYG78365.1 .DATGQDFVMRGVNHAHAWYPD....RTDQALKDIKAKGANSVRVVL.ATGD.......RWAK...NGTADVTHVVEQCKQNRLVCV.LEAH.DTTGYG.EAG...AAVPLSKAVDYWLS..VKD.ALVGQEKYVLINIGNEPYGN..TN
AKG46315.1 .EGNGNDFIMRGVNHAHTWYPG.....ETQSLADVKALGSNTVRVVL.SNGY.......RWSE...NSAADVASVVAQCKANRLICV.LEVH.DTTGYG.EEA...AADTLDGAADYWIG..LQS.VLTGEEDYVIINIGNEPWGN..TD
QKV71638.1 .EGNGNDFIMRGVNHAHTWYPN.....ETQSLADVKALGSNTVRVVL.SNGH.......RWSE...NSPSDVASVIEDCKANRLICV.LEVH.DTTGYG.EEP...EAGTLDGAADYWIG..LKD.VLDGEEDYIIINIGNEPWGN..TN
AQA16021.1 .EGNGNDFIMRGVNHAHTWYPG.....ETRSLADIKALGANTVRVVL.SNGH.......RWSK...NSPEDVAAIIAQCKANRLICV.LEVH.DTTGYG.EDA...AAGSLDEAADYWIG..LKS.VLTGEENYVLINIGNEPWGN..TD
ADI12268.1 .EGNGNDFIMRGVNHAHTWYPG.....ETQSLADIKALGANTVRVVL.SNGH.......RWTK...NSPEDVASIVAQCKANRLICV.LEVH.DTTGYG.EES...AAASLDQAADYWIG..LKS.VLTGEENYIVINIGNEPWGN..TN
AGP60361.1 .EGNGNDFVMRGVNHAHTWYPG.....ETRSLADIKALGANTVRVVL.SNGH.......RWTR...NSPEDVAAIIAQCKANRLICV.LEVH.DTTGYG.EDA...AAATLDQAADYWIG..LKS.VLTGQEDYVIINIGNEPWGN..TD
AQW47637.1 .EGNGNDFVMRGVNHAHTWYPG.....ETRSLADIKALGANTVRVVL.SNGH.......RWAK...NSPEDVAAIVAQCKANRLICV.LEVH.DTTGYG.EDA...AAASLDQAADYWIG..LKS.VLTGQENYVVINIGNEPWGN..TD
AYC43001.1 .EGNGNDFVMRGVNHAHTWYPD.....KLGSLADIKAQGANTVRVVL.ADGH.......RWTA...NSAADVADVIAQCKANRLICV.LEVH.DTTGYG.EES...AAGTLDQAADYWIG..LKG.VLAGQENYIVINIGNEPWGN..TG
AEN10237.1 .EGNGNDFVMRGINHAHTWYPG.....ETQSLADIKATGANTVRVVL.SDGY.......RWSE...NSPEDVASIIARCKAERLICV.LEVH.DTTGYG.EDA...AAGTLDHAADYWIG..LKD.VLDGEEDYVVINIGNEPWGN..AD
AGJ55179.1 .EGNGNDFVMRGVNHAHTWYPG.....ETQSLADIKSFGANTVRVVL.SDGH.......RWTR...NGPAEVAGIIADCKANRLICV.LEVH.DTTGYG.EDA...AAGTLDHAADYWIS..LKD.VLVGQEDYVIVNIGNEPWGN..TD
AWL38642.1 .EGNGNDFVMRGINHAHTWYPG.....ETQSLADIKATGANTVRVVL.SDGY.......RWSR...NSPADVASVISQCKANRLICV.LEVH.DTTGYG.EDS...AAGTLDHAADYWIS..LKD.VLDGEEDYVVINIGNEPWGN..SN
QEU74297.1 .EGNGNDFVMRGVNHAHTWYPG.....ETQSLADIKATGANTVRVVL.SDGH.......RWSE...NSPADVASVVAECKANRLICV.LEVH.DTTGYG.EDA...AAGTLDHAADYWIG..LKD.VLDGEENYVIINIGNEPWGN..TD
AQS65900.1 .EGNGNDFVMRGVNHAHTWYPG.....ETQSLADVKALGANTVRVVL.SDGH.......RWSE...NSPEDVAAVVGQCKANRLICV.LEVH.DTTGYG.EEA...AAGTLDHAADYWIG..LKE.VLAGEEDYVIVNIGNEPWGN..TD
AIJ17911.1 .EGNGNDFVMRGVNHAHTWYPG.....ETQSLADVKALGANSVRVVL.SDGH.......RWSE...NGPADVAAVIEQCKANRLICV.LEVH.DTTGYA.EDA...AAGTLDHAADYWIG..LKD.VLAGQEDYVIVNIGNEPWGN..TD
AIV32327.1 .EGNGNDFVMRGVNHAHTWYPG.....ETQSLADVKALGANSVRVVL.SDGH.......RWSE...NGPEDVAAVIEQCKANRLICV.LEVH.DTTGYG.EDA...AAGTLDHAADYWIG..LKD.VLAGQEDYVVVNIGNEPWGN..TD
```

Continued from previous page

```
AIS01044.1  .EGNGNDFVMRGVNHAHTWYPG.....ETRSLADIKALGANTVRVVL.SDGH.......RWTR...NGPEDVAAVVGDCKANRLICV.LEVH.DTTGYG.EEA...AAGTLDHAADYWLS..LED.VLAGEEDYVIVNIGNEPWGN..TN
AKZ60014.1  .EGNGNDFVMRGVNHAHTWYPG.....ETQSLADIKAKGANTVRVVL.SDGH.......RWSE...NSPADVAAVVADCKANRLICV.LEVH.DTTGYG.EDA...AAGTLDHAADYWIG..LKD.VLAGEEDYVIVNIGNEPWGN..TD
ALC19130.1  .EGNGNDFVMRGVNHAHTWYPG.....ETQSLADIKALGANTVRVVL.SSGH.......RWTE...NSPADVAAVVADCKANRLICV.LEVH.DTTGYG.EEA...AAGTLDQAADYWIG..LRD.VLTGEEDYVVINIGNEPWGN..TD
ARQ67834.1  .EANGNDFVMRGVNHAHTWFRN.....ETDALGHIKALGANTVRVVL.SSGH.......RWER...NDEADVAAVIDNCKRNRLICV.LEVH.DTTGYG.EQS...GAASLDQAADYWID..VQG.ALAGQEQYVILNIGNEPYGN..SN
AWT46021.1  .EGNGNDFVMRGVNHAHTWYPG.....QTRSLADIKALGANTVRVVL.SDGY.......RWTR...NTPEDVAAVIADCKANRLICV.LEVH.DTTGYG.EDS...AAGTLDHAADYWIS..LKD.VLAGQESYVIVDIGNEPWGN..TN
BAK26781.1  .EGNGNDFVMRGVNHAHTWYPG.....QTRSLADIKALGANTVRVVL.SDGH.......RWTR...NGPADVAAVIDRCKANRLICV.LEVH.DTTGYG.EEP...AAGTLDHAADYWIS..LMD.VLAGQEDYVIVNIGNEPWGN..TD
QCB26303.1  .EANGNDFVMRGVNHAHTWYPG.....ETQSLADIKALGANSVRVVL.SDGH.......RWSE...NSPADVANVVAQCKANRLICV.LEVH.DTTGYG.EDA...AAGTLDHAADYWIS..LKD.VLAGEEDYVIVNIGNEPWGN..TD
QEU63996.1  .EGNGNDFVMRGVNHAHTWYPG.....ETQSLADVKALGANTVRVVL.SDGH.......RWTK...NSPEDVAAVIGQCKANRLICV.LEVH.DTTGYG.EEA...AAGTLDHAADYWIG..LKD.VLAGQEDYVIVNIGNEPWGN..TA
QFQ95700.1  .EANGNDFVMRGVNHAHTWYPG.....QTRSLSDIKALGANTVRVVL.SDGH.......RWTR...NSPADVASVVAQCKANRLICV.LEVH.DTTGYG.EEA...AAGTLDHAADYWIS..LKD.VLAGEENYVIVNIGNEPWGN..TD
QFX79702.1  .EGNGNDFVMRGVNHAHTWYPA.....ETQSLADVKALGANSVRVVL.SDGY.......RWSE...NGPEDVAAVIEQCKANRLICV.LEVH.DTTGYG.EDT...AAGTLDHAADYWIG..LKD.VLAGQEDYVVVNIGNEPWGN..TD
QIS75621.1  .EGNGNDFVMRGVNHAHTWYPG.....ETQSLADVKALGANTVRVVL.SDGY.......RWSR...NSPQDVAAVIQNCKANRLICV.LEVH.DTTGYG.EDS...AAGTLDHAADYWIS..LKD.VLAGQEDYVVINIGNEPWGN..TN
QNP67578.1  .EANGNDFVMRGVNHAHTWYPG.....ETQSLADIKAKGANSVRVVL.SDGH.......RWSE...NSPADVAAVVADCKENRLICV.LEVH.DTTGYG.EDA...AAGTLDHAADYWIG..LKD.VLDGEEDYVIVNIGNEPWGN..TN
QQM46492.1  .EGNGNDFIMRGVNHAHTWYPG.....ETRSLADVKALGANTVRVVL.SNGH.......RWTR...NSAADVAAVVAQCKANRLICV.LEVH.DTTGYG.EDA...AAGTLDQAADYWIG..LKD.VLVGEENYVIVNIGNEPWGN..TN
QWB27515.1  .EASGNDFVMRGVNHAHTWYPG.....ETQSLADVKSLGSNTVRVVL.SDGH.......RWTK...NSAQDVASVIARCKANRLICV.LEVH.DTTGYG.EES...AAGTLDHAADYWIS..LKE.VLAGQEDYVIINIGNEPWGN..TN
QJT06651.1  .ESNGNDFVMRGVNHAHTWYPG....RTQQSLADIKAMGANAVRVVL.ADGH.......RWSA...NSASDVATVVAQCKANRLICV.LEVH.DTTGYG.EDT...AAGTLDQAADYWIG..LKD.VLAGQENYIIVNIGNEPWGN..TD
AZS88981.1  .EGNGNDFIMRGVNHAHTWYPG....RTQQSLADIKALGANAVRVVL.ADGH.......RWSA...NSASDVAGVISQCKANRLICV.LEVH.DTTGYG.EDS...AAGTLDQAADYWIG..LKS.VLAGQEDYTIINIGNEPWGN..TD
CCK29425.1  .ESNGNDFVMRGVNHAHTWYPG.....EPQSLADIKALGANSVRVVL.SNGH.......RWTE...STPEDVAAVIANCKANRLICV.LEVH.DTTGYG.EEA...AAATLDSAADYWIG..LKD.VLAGQENYVIVNIGNEPWGN..TD
QIJ66767.1  .EGNGNDFVMRGVNHAHTWYPG.....ETQSLADIKKLGANTVRVVL.SDGH.......RWTR...NSPADVAAVVDDCKANRLICV.LEVH.DTTGYG.EEA...AAGTLDHAADYWIS..LKD.VLAGEEDYVIVNIGNEPWGN..TN
QZZ26035.1  .ESNGNDFVMRGVNHAHTWYPT....RLDQSLADIKAFGANTVRVVL.ADGH.......RWTA...NTASDVGDVIAQCKANRVICV.LEVH.DTTGYG.EES...AAGTLDQAADYWIG..LKS.VLAGQEDYIAINIGNEPWGN..TN
SHI71357.1  .EGNGNDFVMRGVNHAHTWYQG....KTQQSLADIKAMGANTVRVVL.ADGH.......RWTA...NSAADVAAVVAQCKANRLICV.LEVH.DTTGYG.EDG...AAGTLDQAADYWIG..LKD.VLAGQENYIIVNIGNEPWGN..SS
ANS63452.1  .EANGNDFVMRGVNHAHTWYPN.....ETQSLADVKALGANTVRVVL.ADGH.......RWTA...NSAADVANVITQCKANRLICV.LEVH.DTTGYG.EEA...AAGTLDHAADYWIG..LKN.VLAGQENHIIINIGNEPWGN..TN
ALV37404.1  .EGNGNDFVMRGVNAAHTWYPG....KTKQSLADIKALGANSVRVVL.ADGH.......RWAA...NSASDVAAVVAQCKANRLICV.LEVH.DTTGYG.EDS...AAGTLDQAADYWIG..LKD.VLAGQENYIVIDIGNEPWGN..TD
AVV46344.1  .EGNGNDFVMRGVNHAHTWYPG....RTQQSLADIKAMGANAVRVVL.ADGH.......RWSA...NSPADVANVIAQCKANRLICV.LEVH.DTTGYG.EET...AAGTLDHAADYWIG..LKS.VLAGQEDYVIVNIGNEPWGN..TD
AZP15527.1  .EGNGNDFVMRGVNHAHTWYPN.....ETQSLSDIKALGANAVRVVL.ADGH.......RWTA...NSAADVGNVVSQCKANRLICV.LEVH.DTTGYG.EEA...AAGTLDQAADYWIG..LKD.VLAGQENYIIINIGNEPWGN..TN
AZQ39351.1  .EGNGNDFVMRGVNHAHTWYPN.....QTRSLADIKALGANTVRVVL.ADGH.......RWTA...NSASDVANVISQCKANRLICV.LEVH.DTTGYG.EEA...AAGTLDQAADYWIG..LKS.VLEGQENYVVINIGNEPWGN..TN
CBG74852.1  .EGNGNDFVMRGVNHAHTWYPG.....ETQSLADIKALGANTVRVVL.SDGH.......RWTK...NSAADVANVVAQCKANRLICV.LEVH.DTTGYG.EEA...AAGTLDHAADYWIS..LKD.VLAGEEDYVVVNIGNEPWGN..TD
QUC60361.1  .EGNGNDFVMRGVNHAHTWYPS....RLGSSLADIKAQGANTVRVVL.SDGH.......RWTA...NGASDVADVIAQCKADKLICV.LEVH.DTTGYG.EDS...AAGTLDQAADYWIG..LKN.VLAGQENYIIINIGNEPWGN..TN
QKW47771.1  .EANGKDFVMRGVNHAHTWYPG.....EKQSLADIKAFGANTVRVVL.SDGH.......RWTR...NGPDEVAGIVDDCKNNRLICV.LEVH.DTTGYG.EDA...AAGSLDHAADYWIG..LKD.VLAGEEDYVIINIGNEPWGN..TD
QXQ95506.1  .EANGSDFVMRGVNHAHTWYPG.....ETQSLADVKALGANTVRVVL.SDGH.......RWTR...NGPDEVAGIVDDCKTNRLICV.LEVH.DTTGYG.EDT...AAGTLDHAADYWIS..LKD.VLAGEEDYVIVNIGNEPWGN..TD
AYY15149.1  .DANGNDFVMRGVSHPHAWFGQ.....ELGSLEDISSLGANAVRVVL.GSGQ.......RWGP...NDAADVAGVVDECQQHELVCM.LEVH.DTTGYG.EES...EAATLDEAVDYWID..IQS.AVEGQEDYVLINIGNEPYGNDEET
QUX25221.1  VDAAGNDFVMRGVNHAHTWYPQ.....ETGSLADIKELGANTVRVVL.SSGD.......RWAE...NGVADVAAVVDDCKANRLICV.LEVH.DTTGYG.EQS...GAATLDQAVDYWLR..VQD.ALHGEEDHVLVNIGNEPYGNDQAT
APC36031.1  VDAAGNDFVIRGVSHAHTWYAQ.....ETQSFADIKSLGANAVRVVL.SSGQ.......RWTE...NTAADVANVVDECKTNRLICV.LEVH.DTTGYG.DQA...GAATLDQAVDYWLR..IQE.AVEGEEDHILVNIGNEPYGNDAAI
QBI51874.1  VDANGNPFVMRGVNHPHSWFTG.....ETESFGEIASLGANAVRVVL.SNGV.......RWDR...NDAADVANVISECKENQLVCV.LEVH.DTTGYG.EQS...GASTLDQAVDYWID..IQS.ALEGEEAYTILNIGNEPYGN..EG
QKW32020.1  VDAAGNDFLIRGVNHAHTWYLQ.....ETGSFADIKELGANTVRVVL.SSGQ.......RWTE...NGPADVANVVSLCKANRLICM.LEVH.DTTGYG.EQS...GAATLDQAVDYWLR..VQD.VLEGEEDHVLVNIGNEPYGNNDAV
QVJ00172.1  VDAAGNDFVIRGVSHPHTWFTH.....ETGALADIGSLGANTVRVVL.SSGD.......RWQE...NDVADVAAVVADCKENRLVCM.LEVH.DTTGYG.EDP...EAATLDQAVDYWLR..IQD.AVAGEEDHVLVNIANEPFGNDEAT
AAZ54938.1  .EANGQEFIIRGVSHPHNWYPQ.....HTQAFADIKSHGANTVRVVL.SNGV.......RWSK...NGPSDVANVISLCKQNRLICM.LEVH.DTTGYG.EQS...GASTLDQAVDYWIE..LKS.VLQGEEDYVLINIGNEPYGNDSAT
AHB89702.1  .EANGQEFIIRGVSHPHNWYPQ.....HTQAFADIKSHGANTVRVVL.SNGV.......RWSK...NGPSDVANVISLCKQNRLICM.LEVH.DTTGYG.EQS...GASTLDQAVDYWIE..LKS.VLQGEEDYVLINIGNEPYGNDSAT
AID15578.1  .EANGQEFIIRGVSHPHNWYPQ.....HTQAFADIKSHGANTVRVVL.SNGV.......RWSK...NGPSDVANVISLCKQNRLICM.LEVH.DTTGYG.EQS...GASTLDQAVDYWIE..LKS.VLQGEEDYVLINIGNEPYGNDSAT
AHB89703.1  .EANGQEFVMRGVSHAHTWYPQ.....HTRAFADIKSHGANTVRVVL.SNGV.......RWSK...NGPSDVADIISLCKQNRLICM.LEVH.DTTGYG.EQS...GAATLDQAVDYWIE..LKS.VLQGQEDYVLINIGNEPYGNNAAT
AHB89704.1  .EAGGQEFVIRGVSHPHAWYAQ.....ETDSFAGIKSHGANTVRVVL.SNGV.......RWTK...NDAADVANVISLCKQNRLICM.LEVH.DTTGYG.EQS...GASTLDQAVDYWIE..IQS.ALEGQEDYVLINIGNEPYGNDSAT
BBA57841.1  .EANGQEFIIRGVSHPHNWFPQ.....HTGAFADIKSHGANTVRVVL.SNGV.......RWAK...NGPSDVANVISLCKQNHLICM.LEVH.DTTGYG.EEG...AASTLDQAVDYWIE..LKS.VLQGEEDYVLINIGKEPYGNNAAT
PDB         YEANGQEFIIRGVSHPHNWYPQ.....HTQAFADIKSHGANTVRVVL.SNGV.......RWSK...NGPSDVANVISLCKQNRLICM.LEVH.DTTGYG.EQS...GASTLDQAVDYWIE..LKS.VLQGEEDYVLINIGNEPYGNDSAT
ATB37764.1  .DANGNDFIIRGISHPHAWFQQ.....RTSAFADIKVVGANSVRVVL.SGG........RWSV...NTASDVANVISLCKQNRLICV.LENH.DTTGYG.EQG...GAFSLDQAVDYWLS..IRS.ALVGQEAYVIINIGNEPYGN..TL
QRK07611.1  .DANGNDFIIRGISHPHAWYPQ.....RTSSFADIKVVGANSVRVVL.SGG........RWSV...NTASDVANVISLCKQNRLICV.LENH.DTTGYG.EAS...DAYSLSQAVDYWIS..IRS.ALVGQEAYAIINIGNEPYGN..TN
UAC00364.1  .EGHGEPFVMRGISHPYAWYRD.....RTAAFADIKRLGANTVRVVL.............TSD...VTAGEVAGIVERCRNDRLVCV.LENH.DTTGYG.ERP...GAITLDRAAAFWTA..VAP.SLHGTEAFVVVNLGNEPYGN..GD
BAL87525.1  .EANGQKFVMRGVNHMHTWYAN.....QTNSFADIKAAGANTVRVVL.SGG........RWTA...NSAADVQNVINLCKANRLICV.LENH.DTTGYG.EDA...AAYTLDQAVDYWIG..LKS.VLTGQEDYVVINIGNEPIGN..TN
BBH67620.1  .EANGQKFVMRGVNHAHTWYAG.....QTGSFADIKAAGANTVRVVL.SGG........RWTA...NSASDVQNVIDLCKANKMICV.LENH.DTTGYG.EDA...AAYTLDQAVDYWIG..LKS.VLVGQEDYVVINIGNEPIGN..TN
SDT42643.1  .EANGSTFVMRGINHMHTWYAG.....QTQSFADMKAAGANTVRVVL.SGG........RWTA...NTAADVTNVISLCKANRLICV.LENH.DTTGYG.EDS...AAYTLDQAVNYWIG..LKS.VLVGQENYVVINIGNEPIGN..TN
BCJ48281.1  .EANGNKFVMRGVNHAHTWYTS.....QTSSFAAIKAAGANTVRVVL.SGG........RWTA...NTAGDVANVISLCKANKLICV.LEDH.DTTGYG.EDS...AAYTLDQAVNYWIS..LKS.VLAGQEDYISINIGNEPIGN..TN
BCY10256.1  .EANGAKFVMRGVNHAHTWYTS.....QTSSFANIKAAGANTVRVVL.SGG........RWTA...NTASDVANVISLCKANKLICI.LEDH.DTTGYG.EDS...AAYTLDQAANYWIS..LKS.VLQGQEDYVAINIGNEPIGN..TN
BCJ76695.1  .EANGQKFIMRGVNHPHVWYTG.....QTSSFANIKAAGANTVRVVL.GSGK.......RWG....PSNDVANVITLCKTNKLICV.LEVH.DTTGYG.EEG...AAASLDEAVNYWIS..QKS.ALVGQENYVVINIGNEPIGN..VN
UAB94120.1  .EANGQQFIMRGVNHPHVWYTG.....NTQAFADVKALGANTVRVVL.GSGK.......RWG....PSNDVANVISLCKQNKLICV.LEVH.DTTGYG.EDG...AAATLDEAVNYWIS..QKS.VLVGQESYISINIGNEPIGN..NN
AVT28826.1  .EANGQTFVMRGVNHPHVWFTG.....QTRSFADIKALGANTVRVVL.GSGK.......RWG....PSTDVAEVISLCKQNKLICV.LEVH.DTTGYG.EEA...AAASLDEAVDYWIS..QKS.ALVGQENYVVINIGNEPIGN..TN
ACU38007.1  .EANGQPFVMRGVNHPHVWYTG.....RTSSFADVKALGSNTVRVVL.GSGK.......RWG....PSSDTAAVIALCKQNKLICV.LEVH.DTTGYG.EEA...AAASLDEAVDYWIS..QKS.ALVGQEDYVVINIGNEPIGN..TN
BBB03671.1  .EANGREFIMRGVNHAHTWYTS.....QTGSFANIKALGANTVRVVL.GSGK.......RWG....PSTDVANVISLCKQNKLICV.LEVH.DTTGYG.EEG...AAASLDEAADYWIS..QKS.ALVGQENYVVINIGNEPIGN..TN
BCB90671.1  .EANGQRFVMRGVNHPHVWFTG.....QTSSFANIKALGANTVRVVL.GSGK.......RWG....PSTDVADVVNLCKQNRMICV.LEVH.DTTGYG.EEG...AAASLDEAVNYWIS..QKS.ALVGQENYVVINIGNEPIGN..TN
BCJ68966.1  .EANGQPFVMRGVNHPHVWFTG.....QTDSFADVKALGANTVRVVL.GTGK.......RWG....PSNDVADVIALCKQNRLICV.LEVH.DTTGYG.EEG...AAASLDEAVDYWIS..QKS.ALVGQENHVVINIGNEPIGN..VN
BCL17300.1  .EANGQPFVMRGVNHPHVWFTG.....RTDSFADVKALGANTVRVVL.GTGK.......RWG....PSHDVAQVISLCKENRLICV.LEVH.DTTGYG.EEG...AAASLDEAVDYWIS..QKS.ALVGEEDYVVVNLGNEPIGN..VD
QFY13395.1  .EANGNEFIMRGVNHPHVWFTG.....QTGSFADIKAAGANTVRVVL.GSGQ.......RWG....PSNDVANVISLCKQNRLICV.LEVH.DTTGYG.EEG...AAASLDEAADYWIS..QKS.VLAGQENHVVINIGNEPIGN..TN
QFZ19526.1  .EANGQPFVMRGVNHPHVWFTG.....RTSSFADVKALGANTVRVVL.GSGK.......RWG....PSTDTAAVIDLCKRNRLICV.LEVH.DTTGYG.EEG...AAASLDEAVDYWIS..QKS.ALVGQENHVVINIGNEPIGN..VN
QQQ79259.1  .EANGQPFVMRGVNHPHVWYTG.....RTNSFADVKALGANTVRVVL.GTGK.......RWG....PSTDTAAVVDLCKRNRMICV.LEVH.DTTGYG.EEA...AAASLDEAADYWIS..QKS.VLVGQEDYVVVNIGNEPIGN..TD
SBT42754.1  .EANGQTFIMRGVNHPHVWYTG.....QTSSFANIKALGANTVRVVL.GSGK.......RWG....PSTDVANVISLCKQNKLICV.LEVH.DTTGYG.EES...AAASLDEAANYWIS..QKS.ALVGQESYVVINIGNEPIGN..VN
SCF29942.1  .EANGQPFVMRGVNHPHVWFTG.....QTDSFADVKALGANTVRVVL.GTGK.......RWG....PSNDVAEVVSLCKQNKLICV.LEVH.DTTGYG.EEG...AAASLDEAVDYWIS..QKS.ALAGEEDYVVINIGNEPIGN..VN
QIL90192.1  .DGNGHVFVMRGVNHAHTWYPG....RTPQALADIAAAGANAVRVVL.ATGG.......RWNR...NSGANVAAVIDQCKANSLICV.LEVH.DATGWG.EDA...AAIHISEAAEYWVSPDIVQ.ALSGQEDFVLINIANEPFGNS.VS
AMX03424.1  .DANGEPFVMRGVNHAHTWYRD....KTPQALADIAATGANAVRVVL.ATGG.......RWSR...NDGADVADVIARCKSLSLICV.LEVH.DATGWG.EDG...AAIHISEAAQYWISPDIRA.AIEGQEDYILINIANEPFGNT.VS
QEY11805.1  .DPNGNSFVFRGVNHAHTWYTD....RLNKSLSDIAATGANSVRVVL.SNGT.......QWTR...NNGADVTNVINQCKVNKLVCV.LEVH.DSTGFG.ESA...AATHISRATEYWLSADIKA.AITGQEDYVVINIANEPFGNS.TS
QIL90886.1  .DGNGNVFVMRGINHAYTWYTG.....QNGAFADIAAAGANSVRVVL.SNGT.......QWTR...NNGDEVQNIIELCKANKLICV.LEVH.DSTGYP.EQG...SATHISNATNYWLSSDILP.VIQGQEDFVIINIANEPFGNS.VA
QZD54573.1  .DANGNPFVMRGSTHPDVWYQG.....EFESFGELSDLGANTVRVVL.GSGH......RDWGV...SSPARVQQIVDECKAQRLICV.LEVH.DTTGYG.EQA...GAATLDQAVTFWEG..LYS.VLEGEEAYVLINIGNEPIGNQ.DP
ARU50407.1  .EADGSDLVLRGINHAHTWYTH.....EFQSFEDIKGTGANAVRVVL.SSGD.......QWTR...NDPQDVARVVRECDVNRLVCA.LEVH.DTTGYGDEYA..PAAVSLDRAVDYWED..LYP.TLAGTEDRILVNIGNEPIGNG.PI
ANC31527.1  .EADGTDLVLRGVNHAHTWYTH.....TTDSFADIKEAGANSVRVVL.SSGD.......QWTR...NSPEDVANVVDLCEENRLVCV.LEVH.DTTGYGDQYA..PDAATLDQAVDYWED..LYP.TLAGKEDFVMVNIGNEPMGNDDAT
ARU52036.1  .EADGTDLVLRGVNHAHTWYTH.....TTGSFADIKATGANTVRVVL.SSGD.......QWTR...NSPEDVAHVVDLCEENRLVCV.LEVH.DTTGYGDQYA..PDAGTLDQAVDYWED..LYP.TLAGTEDFVLVNIGNEPFGNDATV
AAA71887.1  .........LIGTNHAHCWYRD....RLDTALRGIRSWGMNSVRVVL.SNGY.......RWTK...IPASEVANIISLSRSLGFKAI.ILEVHDTTGYG.EDG...AACSLAQAVEYWKE..IKS.VLDGNEDFVIINIGNEPYGN..NN
ACM60947.1  .........LIGTNHAHCWYRD....KLETALRGIRSWGMNSVRVVL.SNGY.......RWTK...IPASEVANIISLSRSLGFRAI.VLEVHDTTGYG.EDG...AACSLAQAVEYWKE..IKS.VLEGNEDFVIINIGNEPYGN..NN
ACM60953.1  .........LIGTNHAHCWYRD....KLETALRGIRSWGMNSVRVVL.SNGY.......RWTK...IPASEVANIISLSRSLGFRAI.VLEVHDTTGYG.EDG...AACSLAQAVEYWKE..IKS.VLEGNEDFVIINIGNEPYGN..NN
ACM60954.1  .........LIGTNHAHCWYRD....KLETALRGIRSWGMNSVRVVL.SNGY.......RWTK...IPASEVANIISLSRSLGFRAI.VLEVHDTTGYG.EDG...AACSLAQAVEYWKE..IKS.VLEGNEDFVIINIGNEPYGN..NN
ADL42950.1  .........LIGTNHAHCWYRD....KLETALRGIRSWGMNSVRVVL.SNGY.......RWTK...IPASEVANIISLSRSLGFRAI.VLEVHDTTGYG.EDG...AACSLAQAVEYWKE..IKS.VLEGNEDFVIINIGNEPYGN..NN
ADQ45728.1  .........LIGTNHAHCWYRD....KLETALRGIRSWGMNSVRVVL.SNGY.......RWTK...IPASEVANIISLSRSLGFRAI.VLEVHDTTGYG.EDG...AACSLAQAVEYWKE..IKS.VLEGNEDFVIINIGNEPYGN..NN
ADQ45730.1  .........LIGTNHAHCWYRD....KLETALRGIRSWGMNSVRVVL.SNGY.......RWTK...IPASEVANIISLSRSLGFRAI.VLEVHDTTGYG.EDG...AACSLAQAVEYWKE..IKS.VLEGNEDFVIINIGNEPYGN..NN
AZT90847.1  .........LIGTNHAHCWYRD....KLETALQGIRSWGMNSVRVVL.SNGY.......RWTK...IPASEVANIISLSRSLGFRAI.VLEVHDTTGYG.EDG...AACSLAQAVEYWKE..IKS.VLDGNEDFVIINIGNEPYGN..NN
```

Continued from previous page

AEE96309.1	.........LKGVNHAHTWYPD....RLVQALQGIQSWGANSVRVVL.SNGC.......KWAK...NSASDVANVISKAQQLGFTAVILEVH.DTTGYG.EDS...SACSLNDAVAYWKE..IQS.VLIGKEDFVIINIGNEPYGN..NN
AAF22274.1	.........LVGINHPHAWYRD....RLSSSLQGIRSWGANAVRIVL.SNGC.......RWTK...IPASEVADIISQARTLGYRAVVLEVH.DTTGYG.EDA...AACSMTTAVNYWIE..LKN.VLAGQENFVIVNIGNEPYGN..NN
ADN02189.1	........TLRGINHAHTWYKD....RLESALTGIRSWGANSVRVVL.SNGY.......RWTK...DDAASVATIIETAKGLGFSAIMLEVH.DTTGYG.EDG...AACSLAQAVSYWRE..IQA.VLTGEEDFVLLNIGNEPYGN..NN
QCB92788.1	.EENGTPFVARGVSHAHTWYAS....QTPTAIPAIRAAGANALRVVL.SDGT.......RWTR...NDATDVANVISLCRANQLVCM.LEVH.DTTGYG.EQS...GAATLAQAATYWTG..LRS.VLQGTEDFIQINIGNEPYGNTASV
QGQ18559.1	.EENGTPFVARGVSHAHTWYVS....ETPRAVPAIRAAGANALRVVL.SNGT.......RWTQ...NDAADVAGVISLCRANQLVCM.LEVH.DTTGYG.EQS...GAATLAQAAQYWVG..LRS.TLQGTEDFIQINIGNEPYGNNETV
QVI61610.1	.EQDGTPFVARGVSHAHTWYPH....ETDRAIPAIRAAGANALRVVL.SDGG.......RWTQ...NDAADVADVIAQCRANQLVCM.LEVH.DTTGYG.EQS...GAVTLDAAADYWVS..LKP.VLQGTEDFVQVNIGNEPYGNDDAV
QWC15390.1	.EQDGTPFVARGVSHAHTWYTS....QTPTAVPAIRAAGANALRVVL.SGGD.......RWTE...NDAADVAAVIALCKANQLVCM.LENH.DTTGNG.EQS...GAVSLDRAADYFIS..LKS.VLQGTEDFIQINIGNEPTGNNEPQ
ADG75696.1	.ERDGTPFVARGVSHAHTWYTS....RTPTAVPAIRAAGANALRVVL.SGG........RWTA...NSPADVAGVIELCRANELVCM.LENH.DTTGYG.EQS...GAVSLDAAADYFVS..LRS.VLAGTEDFVQINIGNEPTGNNEPQ
ASR55418.1	.EKDGTPFVARGVSHAHTWYTS....QTPTAIPAIRAAGANALRVVL.SGGD.......RWTK...NDAADVANVISLCKANKLICM.LENH.DTTGYG.EQS...GAVTLDTAASYWVG..IRS.ALIGQEDYVQINIGNEPYGNNATA
AEI13136.1	.EKDGTPFVARGVSHAHTWYTS....QTATAIPAIRAAGANALRVVL.SGGD.......RWTK...NDAADVTSIINQCKANQLICM.LENH.DTTGYG.EQS...GAVTLDKAADYWVS..IAS.ALKGQEDYVQINIGNEPYGNNATT
QHT56776.1	.EKDGTPFVARGVSHAHTWYTS....QTATAIPAIRAAGANALRVVL.SGGD.......RWTK...NDAADVANIISLCKANKLVCM.LENH.DTTGYG.EQS...GAVTLDTAANYWVG..IKS.ALIGQEDYVQINIGNEPYGNNATA
QIK83955.1	.DARGVEFVPQGINHAHAWYPS....QTG.SFADIRAAGANTVRVVL.SGG........RYTP...STVSDVADVVARCRANQLVCI.LENH.DTTGYG.EDG...SARSLAQAAQFWTQ..VRS.ALVGQEAYVMINIGNEPFGN..SG
QVI65258.1	.EKDGTPFVARGVSHAHTWYTS....QTATAIPAIRAAGANALRVVL.SGGD.......RWTK...NDAADVASVIALCKANKLICM.LENH.DTTGYG.EQS...GAVTLDTAANYWVS..IAS.VLKGQEDYVQINIGNEPFGNNATT
ACZ29543.1	.EADGTELVLRGISHAHTWYAS.....QTRAFADISDAGANAVRVVL.SDGT.......RWTR...NGADDVAAVIDRCWDEQLVCV.LEDH.DTTGYG.EEG...AASTLATAADYWVS..LLP.VLAGTEDHVIINLGNEPYGNDAAT
AEE43708.1	.DANGNRFVPVGVNHAHAWYPS.....QTQSFADIRAAGANTVRVVL.S.GG.......RYGT...SSAADVSAVVERCKQNQLVCI.LENH.DTTGYG.EDG...SARSLASAAQYWTS..IAS.VLRGQERYVMINIGNEPFGN..SG
AQQ16388.1	.DANGNQFVPVGVNHAHAWYPS.....QTQSFADIRAAGANTVRVVL.S.GG.......RYGT...SSASDVSAVIERCKQNQLVCI.LENH.DTTGYG.EDG...SARSLASAAQYWTS..IAS.VLRGQERYVMINIGNEPFGN..SG
AAO31760.1	.DGNGNPFIMRGVNHPHAWYTS....RTQQALADIASVKANTVRVVL.ADGQ.......QWTR...TTATEVANIINWAKQNKLIVV.LEVH.DVTGSG.EQA...SAGTLYNATTYWLD..MAA.TLQGQEDYVLINIANEPYGNG.VP
ACE84941.1	.DGNGNPFIMRGVNHPHAWYTS....RTQQALADIASVKANTVRVVL.ADGQ.......QWTR...TTATEVANIINWAKQNKLIVV.LEVH.DVTGSG.EQA...SAGTLYNATTYWLD..MAA.TLQGQEDYVLINIANEPYGNG.VP
ARU29988.1	.DGNGNPFIMRGVNHAHAWYTN....RTQQALADIASVEANSVRVVL.SDGQ.......QWTR...TSATEVANIIQWAKNNNLIAI.LEVH.DVTGSG.EQS...SAGTLYNATSYWID..IAS.TLQGQEDYVIINIANEPFGNG.VP
AIF91534.1	.DANGNNFVMRGVNHAHAWYTG.....ETGSIADIANAGANTVRVVL.SNGD.......QWTR...NSQSDVANIVSLCKQNQLICV.LEVH.DATGGG.EES...GVGTIASAAQYWVD..IAG.ALTGEEDYVIINIANEPVGNN.QA
ABD79328.1	.DDNGQAFIMRGVNHPHAWYAN.....QTSSFADIASVGANTVRVVL.SDGQ.......QWTR...NSASDVANVISLCKANKLVCV.LEVH.DVTGSG.EAS...AAGTLANAAQYWVD..IAN.VLKGQEDYVIINIANEPFGNN.VP
ACR11348.1	.DGNGNNFIMRGANHAHTWFTS.....ETSSIANIANLGANTIRVVL.SDGY.......RWNR...NSAADVANIISLCKANQVICV.LEVH.DATGGG.EES...AAGTIAHAADYWVD..IAS.ALIGQEDYVIINIANEPIGNG.QT
AIF91546.1	.DANGNNFIMRGVNHAHAWYSG.....ETGSIADIANTGANTIRVVL.SNGD.......QWTR...NSESDVANIISLCKQNQVICV.LEVH.DATGGG.EES...AAGTIASAAQYWVA..IAG.ALQGQEDYIIINIANEPVGNN.QA
AIF91556.1	.DGNGNAFVMRGINHAHAWYTG.....ETSSFANIAATGANTVRVVL.SNGD.......QWTR...NSESDVANVVSLCKQNQLICV.LEVH.DATGGG.EES...AAGTIASAANYWVA..IAG.ALRGQEDYVIINIANEPVGNN.QP
VAX65844.1	.DANGQSFVMRGVNHPHAWYTG.....ETGSFADIAATGANTVRVVL.SNGH.......QWTR...NSASDVANVISLCKQNQLICV.LEVH.DATGGG.EEG...AAGTIAGAAQYWVD..IAS.ALQGEEDYVIINIANEPVGNN.QP
AJQ96022.1	.DGNDKPFVMRGINYPHSWYST....NTTQAIKDMASVSANTVRVVL.SSGY.......RWNR...SSAEEVTSIIEQLKAQKMIAV.LEVH.DTTGYG.EDS...AASSLSSATDYWLD..IAA.ALKGQEDYVIINIGNEPIGNN.VD
ABC27008.1	....NKPFVMRGINHPHVWYTY....RTEQALNDISAVGANTVRIVL.GSGH.......QWPS...TPAEEVARIIAQCKAKKMIAV.LEVH.DVTGYG.EKP...EAAPLSAAVDYWLS..IQD.TLMGQEDYVIINIGNEPLGNN.VP
AJQ97011.1	.DGNGNPFVMRGINHSHTWFTS....RTTQAIEDIASVKANTVRVVL.SNGY.......RWNR...NSASDVADIIQQLKAHKMIAV.LEVH.DTTGYG.EES...AAASLDSAVDYWLD..IAS.VLEGEEDYVIINIGNEPIGNN.VS
ABD79918.1	VDGNGSTVVLRGVNHPHAWYAG....ETAAAIPKIAATGANSVRVVM.AMGT.......KWSR...TSAAEIQTIIDLCKQNNMIAV.LEFH.DGTGWG.EES...GTAHISDIADYWVSSDVMA.VVKGEEDYVIINIANEPFGNG.VS
BAB79290.2	.DGNGNPFVMRGVNVPHAWFGS....QTSQSLADISATGANSVRVVL.SSGS.......RWSR...TSASDVQSIIDTCKANNLIAV.LEVH.DTTGYG.EQA...GAQTLSGAVDYWLD..IAS.VLQGEEDYVIINIGNEPFGNG.AS
ACR13797.1	.DANDKPFVMRGVNHAHTWYEA....RTQQALIDIESVGANAVRIVL.SNGAH...GEGWGR...DSEQAVAGIIAQMKALEMISI.VEVH.DSTGYP.EKA...GAAPMSTAVDYWLD..IKD.ALIGEEDYVIINIANEPFGNT.AS
BAA25188.1	.EANGSAFKIRGINHAHTWYTD....KLSVALSGIAATGANTVRVVL.SNGY.......RWTK...NDVSDVTNIINLAKANNLIAI.LEVH.DTTGYG.EES...SAASLDSAADYWIE..LKN.ELIGQEDYVIINLGNEPFGNN.ND
ADT88758.1	.EYNGTPFKIRGINHAHAWYAD....KLETALQGISLTGANTVRIVL.SNGH.......RWNK...TSAEEVGNIIQLAKSYNLIAV.LEVH.DTTGYG.EES...SAADLNSAADYWIE..IKE.KLIGEENYVIINLGNEPFGNG.II
AWB65998.1	.DGNGNKFVMRGVNHAHAWFTD....KLDTSLSGIAATGANTVRVVL.SNGH.......RWTK...NSASDVANIIQKAKDHNLVAV.LEVH.DTTGYG.EDG...AAATLSSAVDYWIS..IKN.QLVGQEDYVIINIGNEPFGNN.QA
QMV13106.1	.EGNGHPFKIRGINHAHTWYLN....RLNHALDGIAATGANTVRIVL.SNGQ.......RWNK...NGAVDVANVIRQAKARHLITV.LEVH.DTTGFG.EEW...QAASLDSAADYWIE..LKD.QLVGQEDYVIINLGNEPMGNG.VS
QGY75211.1	.EGNGTPLVLRGVNLPHAWYPD....RSLATIDAIAGEGANSVRVVL.SSGA.......RWKR...TPLSQVADIIARCKRAGLIAV.LEVH.DTTGYG.EDG...AAASLNNAADYWVS..IRN.ALIGQEDYVIVNIGNEPFGNR.FT
AAM41068.1	.ESNGNTLILRGINLPHAWFAD....RTDAALAQIAATGANSVRVVL.SSGH.......RWNR...TPEAEVARIIARCKALGLIAV.LEVH.DTTGYG.EDG...AAGSLANAASYWTS..VRT.ALVGQEDYVIINIGNEPFGNQ.LS
ACE84673.1	.EGNGNNFIMRGVNLPHAWFPD....RTNQALADISATGANSVRVVL.SNGR.......LWSR...TPESQVASIISQAKARQLITV.LEVH.DTTGYG.EQT....AATLSEAVDYWIA..IRN.ALIGQEDYVIINIGNEPFGNG.QS
AMU98328.1	.ESNGNALVLRGVNLPHAWYAG....RTDAALAAIAATGANSVRVVL.SSGY.......RWTR...TPEADVARIIARCKSLGLIAV.LEVH.DTTGYG.EDS...AAANLVNATNYWVA..IRK.ALIGNEDHVVINIANEPFGNS.LT
QKD86610.1	.ESNGNALVLRGVNLPHAWYAG....RTDAALAAIAATGANSVRVVL.SSGY.......RWTR...TPEAEVARIIARCKSLGLIAV.LEVH.DTTGYG.DDT...AAANLVNATNYWVG..IRK.ALIGNEDHVIINIANEPFGNS.LT
APP73913.1	.ETNGSTLVLRGINLPHAWFAD....RTDAALAQTAATGANSVRVVL.SSGH.......RWNR...TPEAEVARIIARCKSLGLIAV.LEVH.DTTGYG.EDG...AAGTLAHAASYWTN..VRG.ALVGQEDYVILNIGNEPFGNQ.LS
APP81017.1	.ESNGNALVLRGVNLPHAWYTS....RTDAALVAIAATGANSVRVVL.SSGY.......RWNR...TPEAEVARIIARCKSLGLIAV.LEVH.DTTGYG.EDG...AAAGLAHATAYWTS..IRK.ALIGNEDHVIINIGNEPFGNR.LS
AVQ07386.1	.ESNGNVLVLRGVNLPHAWYPG....RTDAALAAIAATGANSVRVVL.SSGY.......RWTR...TPEADVARIIARCKSLGLIAV.LEVH.DTTGYG.EDT...SAANLLNATNYWVS..IRK.ALVGNEDNVVINIANEPFGNS.LT
QEY11219.1	...NSSPFVMRGVNVAHAWYND....KTANSLSDIAKTGANSVRVVL.ASGK.......LWSR...TPESQVVSIISQAKANKLITV.LEVH.DTTGYG.EQT....AATLNEAVDYWIS..IKN.SLIGQEDYVIINIGNEPFGNG.QS
QEY19054.1	.DPKGNNFIYRGINHAHVWYPD....RTTQALKDIAATGANSVRVVL.ANGT.......QWTR...TSGATVTDIINQCKTNKLVCV.LEVH.DSTGFG.ESA...AATNISNAASYWASADIKA.AITGQEDYVIVNIANEPYGNN.TS
ACV09865.1	.EADGTELILRGVNHAHAWYNN.....ETGAYADIAHAGANSVRTVL.SNGV.......TWSR...TSSADVAQLISLSEDAQLINM.LEVH.DTTGYGDSSLD.SPRSTLAQAADYWIS..LKD.VLVGTEDHVIINLGNEPFGNNDTT
ACV09866.1	.EADGTELIMRGVNHAHTWYTG.....QTQSFADIGSLGANTVRTVL.SNGV.......KWQR...NDAADVANVIDLAKDAKLISM.LEVH.DTTGYGDQYAD.PPRSTLSEAADYWVD..LKD.VLIGQEDYVMINIGNEPYGNDAAT
ANZ43360.1	.DGHGSTFVMRGVNHPHSWFPN....KTSQALKDIKATGANTARIVL.SNGA.......RFNR...NTTADVASVIKRCKANKLICV.LEVH.DTTGYG.EQS...GASSLTAAVNYWIS..IKS.ALAGQQRYVLLNIGNEPRGN..NN
ACS46797.1	...AGKQFVPQGINHAYQWYPE.....HTSSIGDIANTGANTVRVVL.SGG........RYGT...TSASEVSNIVNECKSNNLVCI.LEDH.DTTGYGDTAYA.ADAKSLSDAVQFWKS..VGS.AVKGQEDYVMINLGNEPYGN..NT
AEK30917.1	...AGKQFVPQGINHAYQWYPE.....HTSSIGDIANTGANTVRVVL.SGG........RYGT...TSASEVSNIVNECKSNNLVCI.LEDH.DTTGYGDTAYA.ADAKSLSDAVQFWKS..VGS.AVKGQEDYVMINLGNEPYGN..NT
AGB26659.1	.ESNGTPFVMRGTNHMFTWHPD.....QWATFADVKRLGANSLRVSL.SGG........RWTP...NGVDDVRAVISECKRNRLICV.LEDH.DTSGYL.TTQ...NEWSLDRAADYWIS..IKD.ALIGQENYVLINIGNEPYRT..VD
AGB26661.1	.QADGTPFVMRGTNHMYTWNRQ.....RTDVFAAVKALGANTLRVVL.SGG........RWEV...DGTAEVASVVALCKQNKLVCV.LEDH.DTTEYP.TAQ...TEWTLDRAADYWIS..LKG.ALIGQEDYVLINLGNEPYTD..EG
UAB95210.1	.EANGSDLVLRGVNHDFIWYPD.....KNGSFAAIKATGANAVRVPL.GMGH.......QFRR...TKPEEVHTIVTLCRQERLICI.LDAH.DTTGFG.QDK...KAATIADAVAFWTS..LRD.ELAGHEDHVIINVANEPFGI..GG
SJX74184.1	.DAGGKTFVIKGVNNPHAWFGD....RAYDALDNIADINSNCVRIVW.QTR..............GNVQELERIIKRCIELEMIPM.VELH.DVTGNP.SKE...RLV.DMAEYYARED..VSS.MLKLYEKYLLINIANEWGDHA.VT
ASS64945.1	.DANGSSFVMRGVNNPHIWFDS....QAYAALTDIAAKGANTVRIVW.STS..............GAASRLQQIIDRCKALKMVSV.IELH.DVTGSN.DAA...RLN.DMAKYFARSD..VKA.VLNSNEKYALVNIANEWGGSD.LS
AET57908.1	.DANGNTFVMRGVNNPHIWFDT....QAYQALDSIAATKSNTVRIVW.QTN..............GSAQRLEQIINRAKQLKLISV.VELH.DVTGSN.DAN...RLN.DMARYFADPA..VKK.ILADNQKYVLVNIANEWGDHN.LT
AXA90579.1	.DANGNAFVARGVNNPHAWFDT....QSFNVLGNLAARKTNLIRVVW.TMN..............GSPARLDQILSAIESHKMVSI.VELH.DGTGSN.DAA...RLQ.AMADYWARAD..VVA.VLRKHERSTMINIANEWGDAG.KT
AGX43683.1	....EDSSWIRGANVPAIWYDS....QSYSALDKVASDKFNTVRLVW.DTS..............GSASRLDEFLTKCDNLELKPI.VELH.DATGGT.KTD......TLNTCVNYWVR.SDIL.SVMYNHPKAWLNIANEWGPA...N
ACY02072.1	.DANGMEFIPKGVNNAHFWFDSGNRHYAFDALSPIAAYYSNSVRMVW.QTNY......QSNSWSDIEEATTLKRMFDEALRLGLVPM.IELH.DVTGDE.SKE...NLL.KMAEFYVRPD..ILS.VMKEYESVLLINIANEWSGR....
AZQ63976.1	.DPNGMEFVARGVNNAHFWFDNGGRDLALNALSAINANSSNMVRLVW.QTDY......NASNWENEEETVRLRKMINTCIANGMVPM.IEMH.DATGGE.NKE...QID.AIVAYYTRPD..ILS.VMKEFESILLINIANEWSGK....
ADX05709.1	.DRCDNEFVMRGVNLAYTWYKS....SAYDQLKAIYNNKANAVRIVL.TNGN......AYGSP..ADDAASVKKLIDACKAYGMVAI.LEVH.DVTGSD.KIS......DLEGAAQYFAN..LAS.TLKGTEHYVIINIANEWHNS..SS

Continued from previous page

130 140 150 160 170 180 190 200 210 220 230 240

ADU21877.1	GYSWAEGNKSAVKSIRNAGIDNMIMVDCAGWGQYP.DSIKDYGKSVFNADSQKNTVFSIHMYEYAGGNSSTVKKNIDNAL.GIGVPVVIGEFGGQ..HTNG......DVDEGTIMSYCTQKNVGYLGWSWKGNGSD.
ADU23074.1	SYNWAEGNKSAVKKLRNADIDNMIMVDCAGWGQYP.ACIKEYGRSVYEADVDRNTVFSIHMYEYAGGNASTIKKNIDDAL.SIDVPVVIGEFGGQ..HTGG......DVDEATIMSYCTQKNVGYLGWSWKGNNSD.
CCO04360.1	GSAWADGYKSAIQSVRNAGITNMLMVDCAGWGQYP.DSIKDYGKSVFNADSQKNTVFSIHMYEYAGGNASTVRNNIDNAL.NIGVPVVIGEFGGQ..HTNG......DVDEATIMSYCTSKGVGYLGWSWKGNNSDM
BAA25878.1	GVAWANGYKQAIPKLRNAGLTHTLIVDSAGWGQYP.DSVKNYGTEVLNADPLKNTVFSIHMYEYAGGNASTVKSNIDGVL.NKNLALIIGEFGGQ..HTNG......DVDEATIMSYSQEKGVGWLAWSWKGNSSD.
AIQ62043.1	GSGWANGYKQAIPKLRNAGLNHTLIVDSAGWGQYP.ASIINYGTEVFNADPQKNTMFSIHMYEYAGGNATTVKSNIDGVL.NKNLALIIGEFGGQ..HTNG......DVDEATIMSYSQEKSVGWLAWSWKGNSSD.
QUL52210.1	GPAWAAGYKQAIPKLRNAGLNHTLIVDSAGWGQYP.ASAINYGTEVLNSDSLKNTVFSIHMYEYAGGNAATVKSNIDEVL.NKNLALIIGEFGGQ..HTNG......DVDEATIMSYSQQKKVGWLSWSWKGNSSD.
QYK67373.1	GGGWAQGYKSVIPKLRNAGIKNTIMVDGAGWGQYP.QSIFDYGTEVFNADPLKNTMFSIHMYEYAGGNAATVKSNMDNVL.NKNLALVIGEFGIK..HTSG......DVDEATIMSYAEQKGVGYLAWSWKGNSAG.
ADO56715.1	GGGWAQGYKSVIPKLRNAGIKNTIIVDGAGWGQYP.QSIFDYGKEVFNADPLKNTMFSIHMYEYAGGNAATVKSNMDNVL.NKNLALLIGEFGIK..HTSG......DVDEATIMSYAEQKGVGYLAWSWKGNSP..
AIQ31305.1	GANWAKGYTAVIPKLRNAGIQNTLMVDGAGWGQYP.QSIVDYGTQVFNADPLKNTMFSIHMYEYAGGNAATVKTNIDNVL.NKNLALVIGEFGIR..HTNG......DVDEAAIMSYSRQKGVGYLGWSWKGNGP..
AUJ88464.1	GNGWAQGYKSVIPKLRNAGIKNTIMVDAAGWGQYP.KSIFDYGTQVFDADPLKNTMFSIHMYEYAGGNAETVKSNIDNVL.NKNLALIIGEFGIK..HTNG......DVDEATIMSYAQQKGVGYLGWSWKGNGSG.
QAA30979.1	SSGWANGYKQVIPKLRDAGIKNTLMVDCAGWGQYP.ASISDYGTDVFNSDKLKNTMFSIHMYEYAGGNASNVKNNIDSVL.NKGLALCIGEFGIR..HTNG......DVDEATIMSYCTQKSVGYLAWSWYGNNSD.
QKS47268.1	GANWAAGYKSVIPKLRNAGINNTIMVDAAGWGQYP.QSIADYGTDVFNADPQKNTMFSIHMYEYAGANAATVKSNIDSVL.NKGLAVVIGEFGIK..HTNG......DVDEATIMSYSQEKGVGYLGWSWKGNGSG.
AAS48170.1	GAAWADGYKQAIPRLRNAGLNHTLMVDAAGWGQFP.QSIHDFGREVFNADPQRNTMFSIHMYEYAGGNASQVRTNIDRVL.NQDLALVIGEFGHR..HTNG......DVDEATIMSYSEQRGVGWLAWSWKGNGP..
BAD99527.1	GAAWADGYKQAIPRLRNAGLNNTLMIDAAGWGQFP.QSIHDYGREVFNADPQRNTMFSIHMYEYAGGNASQVRTNIDRVL.NQDLALVIGEFGHR..HTNG......DVDESTIMSYSEQRGVGWLAWSWKGNGP..
AAQ31841.1	SEGWADGYKKAIPLLREAGLKHTLMVDAAGWGQFP.RSIHEKGLEVFNSDPLKNTMFSIHMYEWAAGNPQQVKDNIDGVL.EKNLAVVIGEFGHH..HYGR......DVAVDTILSHSEKYDVGWLAWSWHGNSGGV
AAT06599.1	GAAWADGYIDVIPKLRDAGLTHTLMVDAAGWGQYP.QSIHDYGQDVFNADPLKNTIFSIHMYEYAGGDANTVRSNIDRVI.DQDLALVIGEFGHR..HTDG......DVDEDTILSYSEETGTGWLAWSWKGNSAE.
AAN27517.1	GSAWADGYIDVIPKLRDAGLTHTLMVDAAGWGQYP.QSIHDYGQDVFNADPLKNTMFSIHMYEYAGGDANTVRSNIDRVI.DQDLALVIGEFGHR..HTDG......DVDEDTILSYSEETGTGWLAWSWKGNST..
AFC30528.1	ASGWANGYKQAIPKLRSAGLDHLLMVDAAGWGQYP.ASIHTMGKEVLAADPRKNTMFSIHMYEYAGGTADQVRSNIDGVL.NQGLAVVVGEFGPK..HSNG......EVDEATIMSYSQQKGVGWLVWSWYGNSSD.
AGU71466.3	GAAWAAGYKQAIPKLRNAGLNHTLIIDSAGWGQYP.ASIHNYGKEVFNADPLKNTMFSIHMYEYAGGDAATVKSNIDGVL.NQGLALIIGEFGQK..HTNG......DVDEATIMSYSQQKNIGWLAWSWKGNSTD.
AZK47289.1	SNGWADGYKSAIPKLRNAGINHTLIVDAAGWGQYP.QSIVDKGNEVFNSDPLRNTIFSIHMYEYAGGNADMVRANIDQVL.NKGLAVIIGEFGHY..HTGG......DVDETAIMSYTQQKGVGWLAWSWKGNGA..
BAM46957.1	GRLWADGYKQAIPRLRDAGLTHTLMIDAAGWGQFP.SSIHQYGREVFNADRLGNTMFSIHMYEYAGGDDQMVRDNINGVI.NQDLALVIGEFGHY..HTDG......DVDEDTILSYAEQTGVGWLAWSWKGNGTE.
CBK96294.1	GELWRDGYTESIPKLREAGIKNTILVDAAGWGQYA.KSIGDYGKEVFDSDPDKNTMFAVHMYGTAGKNSSVIEKNLKYAT.DNGLCVIVGEFGYT..HTDG......DVDEAFIMKYCQDNGIGYIGWSWKGNSGGV
CBL18180.1	TSSWESAYIAAVKEIRDAGLTHCIMCDAGGWGQGY.VTVRDGGSAVLASDPEKNVMFSVHMYGTAGGNAATIKSVIDGIM.TRNLCLVIGEFGYK..HSDG......EVDEAYIMEYCKDVQIGWMAWSWYGNGSPV
QGG58625.1	GSGWANGYKAAIPKLRNAGLTHTLMVDSAGWGQYP.DSIRDYGQSVLAADPQRNTMFSIHMYEYAGGNAQTIKTNIDNVL.NKGLCLTIGEFGQY..HTNG......DVDEPTILSYAQQKKVGWTAWSWHGNGSS.
APO45595.1	GSAWADGYKKAIPKLRNAGIKNTLIVDAAGWGQFP.QSIVDYGQSVFAADSQKNTVFSIHMYEYAGKDAATVKANMENVL.NKGLALIIGEFGGY..HTNG......DVDEYAIMRYGQEKGVGWLAWSWYGNSSG.
AAX87003.1	GSAWADGYKKAIPKLRDAGIKNTLIVDAAGWGQYP.QSIVDYGQSVFAADSQKNTAFSIHMYEYAGKDAATVKSNMENVL.NKGLALIIGEFGGY..HTNG......DVDEYAIMKYGLEKGVGWLAWSWYGNSSG.
QBF51372.1	GSAWADGYKKAIPKLRDAGIKNTLIVDAAGWGQYP.QSIVDYGQSVFAADSQKNTAFSIHIYEYAGKDAATVKSNIENVL.NKGLALIIGEFGGY..HTNG......DVDEYAIIKYGLEKGVGWLAWSWYGNG...
AEX60762.1	GSAWADGYKQAIPKLRNAGIKNTLIVDAAGWGQCP.QSIVDYGQSVFAADSLKNTIFSIHMYEYAGGTDAIVKSNMENVL.NKGLPLIIGEFGGQ..HTNG......DVDEHAIMRYGQQKGVGWLAWSWYGNNSE.
ANF96694.1	GSAWADGYKQAIPKLRNAGIKNTLIVDAAGWGQYP.QSIVDYGQSVFAADSQRNTIFSIHMYEYAGKDAATVKANIDGVL.NKGLPVIIGEFGGY..HTNG......DVDEYAIMRYGQEKGIGWLAWSWYGNSSN.
AAX87002.1	SSGWADGYKQAIPKLRNAGIKNTLIVDAAGWGQYP.QSIVDEGAAVFASDQLKNTVFSIHMYEYAGKDAATVKTNMDDVL.NKGLPLIIGEFGGY..HQGA......DVDEIAIMKYGQQKEVGWLAWSWYGNSPE.
ADM68451.1	GSGWADGYKQAIPKLRNAGIKNTLIVDCAGWGQYP.QSINDFGKSVFAADSLKNTVFSIHMYEFAGKDAQTVRTNIDNVL.NQGIPLIIGEFGGY..HQGA......DVDETEIMRYGQSKGVGWLAWSWYGNSSN.
ANY76761.1	GREWADGYKQAIPKLRNAGIKNTLIVDAAGWGQQT.ESIAEYGTAVSEADPQRNIIFSIHMYEYAGKDAATVKANMELVL.SQGLPLIIGEFGGY..HQGA......DVDEMAVMKYGQEKGVGWLAWSWYGNSP..
CQR54354.1	TPAWASGYQSAIPALRAAGIKNTLVVDAAGWGQYP.SSIFTSGNAVFNSDPLANTIFSIHMYEYSGGTAATVKSNIDNAL.AIGVPVIIGEFGWK..HSGG......DVDEAAIMSYSQEKGVGWLAWSWYGNGGGV
AIQ19732.1	TATWASGYQSAIPALRAAGIRNTLVVDSAGWGQYP.GSIFNSGNAVFSSDPLKNTIFSIHMYEYAGGTATTVKSNIDSVL.ALGLPVIIGEFGYK..HTSG......DVDEATIMSYSQQIGVGWLAWSWYGNGGGV
AIQ75360.1	TATWASGYQAAIPALRNAGIKNTLVVDAAGWGQYP.QSIFTSGQAVYNSDTLKNTIFSIHMYEYAGGNAATVKSNIDSAL.AIGAPVIIGEFGDK..HTGG......DVDEATIMSYTKEKKVGWLAWSWYGNGGGV
AWV34670.1	TATWASGYQAAIPALRNAGIKNTLVVDAAGWGQYP.QSIFTSGQAVFNSDTLKNTIFSIHMYEYAGGNAATVKSNIDSAL.AIGAPVIIGEFGDK..HTGG......DVDEATIMSYTKEKKVGWLAWSWYGNGGGV
QMV43160.1	GPAWANAYKTMIPQLRNAGLTHALVVDAAGWGQYP.ASIHNYGQEVLASDTLGDTVFSIHMYEYAGGNAATVKANIDGAL.NKGLAVIVGEFGYQ..HTSG......DVDEATIMSYSQQKGVGWLAWSWYGNGSSV
ACU52526.1	TAGWADGYKVVIPELRNAGLEHLLVVDTAGYGQYP.QAIFEKGKEVFQTDLLARTVFSIHMYEYAATDVTMIKGNIDSAL.NTGIPVIIGEFGDR..KPES.....QHVDIDTIMSYTREKSVGWLAWSWYGNGND.
AAT39478.1	SDVWAEAYAQAIPRLRSAGLSHTLMVDAAGWGQYP.ASIHERGADVFASDPLKNTMFSIHMYEYAGADRATIAYNIDRVL.AENLAVGIGEFGHS..HHDG......DVDEDAILAYTAERQVGWLAWSWYGNSGGV
AST98531.1	SDVWAEAYAQAIPRLRSAGLAHTLIVDAAGWGQYP.ASIHERGADVFASDPLKNTMFSIHMYEYAGADRATVSENIDGVL.AENLAVVIGEFGHR..HHDG......DVDEDAILAYTAERQVGWLAWSWYGNSGGV
AAQ31839.1	GGAWARGYQNAIRQLRNAGLSHTFMVDAAGYGQYP.QSVVDYGQEVLNADPQRNTMFSVHMYEYAGGDANTVRRNIDSIL.SQNLALVIGEFGHW..HYDG......DVDEDTILSYSQQRNVGWLAWSWHGNSEGV
CDM68969.1	GKKWAEGYKQAIPKLRNAGIKNTIMIDCAGWGQYP.KSIHNYGKEVFNADSEKNTMFSIHMYEYAGGDADTIKNNIDGVL.NQGLALSIGEFGIK..HTNG......DVDEKTIMSYCQEKSVGYIGWSWYGNGDE.
QUI25581.1	SQGWANGYKQAIPRLRNAQLKHTLLIDCAGWGQYP.KSVHDLGKEVFNADPEKNTMFSIHMYEYAGGNATDIKHNIDGVL.NQGLAVCIGEFGLR..HTDG......DVDEATIMRYCQEKGVGWIGWSWHGNGD..
ALP89769.1	GQKWAEGYEKAIPKLRAAGIDNTIMVDSAGWGQYP.KSIADYGKAVFNSDPDKNTMFSIHMYEYAGGDANTVKTNIDSVI.NQNLALCIGEFGIK..HTNG......DVDEETIMNYCQEKSVGYIGWSWYGNGTD.
BBG30007.1	SYAWSIGYEFAITALREAGFEHLLVIDGAGYGQYP.QSIIDYGQALLKSDRAGRLAFSLHMYEYAAADTATIKRNIDEIL.ALNLPLIIGEFGDR..VPES.....HPVDIGMLMRYAEETAVGWIAWSWYGNDGS.
QGW99981.1	SSNWAAGYQQVIPKLRNAGIKNTIMVDCAGWGQYP.KSIADAGQSVLQSDSQKNIMFSIHMYEYAG.AYGAVQNNIDSSL.AVGAPLCIGEFGIK..HTNG......PIDYKTIMSYSQAKGVGYLGWSWKGNSSE.
AQP41170.1	SSNWAAGYQQVIPKLRNAGIKNTLMVDCAGWGQYP.QSIAEAGQSVVQADSQHNIMFSIHMYEYAG.AYGAVQSNIDSAL.NVGVPLCIGEFGIR..HTNG......DVDYKTIMSYSQSKGVGYLGWSWKGNGD..
QGX44134.1	SANWAAGYQQVIPKLRNAGIKNTLMVDCAGWGQYP.QSIAEAGQSVVQADSQHNIMFSIHMYEYAG.AYGAVQSNIDSAL.NVGVPLCIGEFGIK..HTNG......PVDYKTIMSYSQSKGVGYLGWSWKGNGD..
ATC23771.1	PSPWFEAHRTAIVALRAGGLKHLLMVDGDDYGQDGRGAMRDRAAELLAADPLRNTVFSVHMYQVYG.RPEAVTAYLDAFQ.AANLPLVIGEFGPD..HMGD......PVAEETILSEAARRRIGWLAWSWSGNSPKT
AYV49609.1	PEQWLEAHRTAIIALRALGLRHTLVVDAHDYGQDRSGTMRDRAAQLFAADPLRNVVFDVHMYEAYG.EAQAVERYLRAFE.TARLPLIVGEFGPD..HRGQ......PVDEEAIFRLAGAMGVGYLGWSWSGNAQEV
BCJ57634.1	YTSWTTDTANAIRRLRTGGIQHTIMVDAPNWGQDWAFTMRDTAPTVFNADPARNTVFSVHMYGVFD.TAAEISDYLGRFR.SRGLPIVVGEFGHN..HSDG......NPDEETILSYTQANGIGWLGWSWSGNSGGV
QGN49881.1	YGTWAGDTSNAITRLRAADLDHTIMVDAPNWGQDWTFTMRDNASTVFAADPDRNTVFSIHMYGVFD.TAAEISDYLGRFR.AARLPIVVGEFGHN..HSDG......NPDEDTILSYSQANGIGWLGWSWSGNGGGV
SCG72161.1	YGTWATDTANAIKRLRANGISHTIMVDAPNWGQDWSFTMRDNAASVFNADPQKNTVFSIHMYGVFD.TAAEVSDYLGRFR.SAGLPIVVGEFGHN..HSDG......NPDEDAILSYSQANGIGYLGWSWSGNGGGV
ADJ49127.1	SATWTSATSNAIKRLRAAGLQHLLMADAPMWGQDWQNIMRDNAGTVFNADPQHNTVFSIHMYGVYD.TAAEINAYFDAFR.TAGLPLVVGEFGSM..HTDG......NPDEDTIMAQAQARGLGYLGWSWSGNSSDV
ANN17163.1	SATWTSATSKAITRLREAGLRHLLMADAPMWGQDWQNIMRDNAAAVLNADPQRNTVFSVHMYGVYD.TAAEITAYFDAFQ.AANLSLVVGEFGHN..HSDG......DPDENTIMAQAQARGLGYLGWSWSGNSSEV
ASW54425.1	YSTWATDTANAVKRLRSNGFNHTIMVDAPNWGQDWTFTMRDNASTVWNADPNKNTLFSIHMYGVFD.TAAEVTDYLGRFR.TAGLPIAVGEFGFD..HSDG......NPDEDTIMSYSQANGIGYLGWSWSGNSGGV
ATO14192.1	YSAWTTDTSNAIKRLRAGGLTHTIMVDGPNWGQDWSFTMRDNAGTVFNADPQRNTVFSIHMYGVFD.TAAEISDYLGRFR.SAGLPIVVGEFGFN..HSDG......NPDEDAIMSYSQANGIGYLGWSWSGNGGGV
AXO36964.1	YSAWTTDTSNAIKRLRAAGLTHTIMVDAPNWGQDWSFTMRDNASTVFAADAQRNTVFSIHMYGVFD.TAAEISDYLGRFR.TAGLPIVVGEFGFN..HSDG......NPDEDAIMAYSQVNGIGYLGWSWSGNGGGV
QDY07284.1	YGTWATDNANAIKRLRAGGITHTIMVDAPNWGQDWSFTMRDNAAGVFAADPQKNTVFSIHMYGVFD.TAAEISDYLGRFR.TAKLPIVVGEFGFD..HSDG......NPDEDTILSYTQANGIGYLGWSWSGNGGGV
QLD24471.1	YGTWATDSANAIKRLRAAGLAHTIMVDAPNWGQDWSFTMRDNAASVFAADPQKNTVFSIHMYGVFD.TAAEISDYLGRFR.TAKLPIVVGEFGFD..HSDG......NPDEDAILAYTQANGIGYLGWSWSGNGGGV
SBT37134.1	YGAWTNDTANAIKKLRAGGLTHTIMVDGPNWGQDWSFTMRDNAGTVFNADPQKNTVFSIHMYGVFD.TAAEITDYLGRFR.SAGLPIVVGEFGFN..HSDG......NPDEDTIMSYSQANGIGYLGWSWSGNGGGV
SBT38821.1	YSTWATDTANAIKRLRAGGLTHTIMVDGPNWGQDWSFTMRDNAATVFNADPSKNTIFSIHMYGVFN.TAAKVSDYLDRFR.TAKLPILVGEFGNL..HSDG......DPDEDAIMAYSQTNGIGYLGWSWSGNGGGV
SCE69983.1	YSGWATDTANAIKRLRTAGFAHTIMVDGPNWGQDWTYTMRDNAASVFAADPAKNTVFSIHMYGVFN.TAAKISDYLGRFR.TAGLPIVVGEFGHN..HSDG......DPDEDAILSYSQANGIGYLGWSWSGNGGGV
SCE72877.1	YASWATDNATAITRLRAGGLTHTIMVDAPNWGQDWSFTMRDNAASVFAADPARNTVFSIHMYGVFD.TAAEISDYLGRFR.AAGLPIVVGEFGFN..HSDG......DPDEDTILAYSQANGIGWLGWSWSGNGGGV
SCF27982.1	YGSWATDSADAIKRLRAAGLTHTIMVDAPNWGQDWGFFMRDNAASVFAADPARNTVFSIHMYGVFD.TAAEISDYLGRFR.SVGLPIVVGEFGFN..HSDG......DPDEDAILAYTQANGIGWLGWSWSGNGGGV
SCF36577.1	YATWATDSANAVKRLRAGGLAHTIMVDAPNWGQDWSFTMRDNAATVFNADPAKNTVFSIHMYGVFD.TAAEITDYLGRFR.SAGLPIVVGEFGFN..HSDG......NPDEDTILSYSQANRIGWLGWSWSGNGGGV
SCG17558.1	YGTWATDTANAIKRLRAGGLTHTIMVDAPNWGQDWSFTMRDNAASVFAADPQKNTVFSIHMYGVFD.TAAEISDYLGRFR.AAKLPIVVGEFGFD..HSDG......NPDEDTILSYSQANGIGYLGWSWSGNGGGV
SCG50027.1	YGAWTNDTANAIKKLRAGGIANTIMVDGPNWGQDWSFTMRNNASTVFNADPQRNTVFSIHMYGVFD.TAAEITDYLGRFR.SAGLPIVVGEFGFN..HSDG......NPDEDTIMSYAQANGIGYLGWSWSGNGGGV
SCG65882.1	YAAWTTDTSNAIKRLRAAGFDHTIMVDAPNWGQDWAFTMRDNATSVFAADPDRNTVFSVHMYGVFD.TAAEISDYLGRFR.SAGLPIVVGEFGHN..HSDG......DPDEESIFSYTQANGIGWLGWSWSGNGGGV
AEB43751.1	YSSWATDTSNAIRRLRNAGLTHTIMVDGPNWGQDWSFTMRDNAPTVFNADPARNIVFSIHMYGVFD.TAAEISDYLGRFR.SRQLPIVVGEFGHY..HSDG......DPDEDAILSYTQANGIGWLGWSWSGNGGGV
AUS77477.1	YEAWTADTIGAIERLRSAGFTHTLLVDAPNWGQDWTFTMRDNAEEVFASDPAGNTVFSIHMYGVYD.TATKVSDYLAAFQ.DANLPLVIGEFGHY..HSDG......DPDEDAIMAGAESRGLGYIGWSWSGNSTEV
QKW13015.1	YSSWATDTSNAIRRLRTAGIAHTIMVDAPNWGQDWSFTMRDNAPTVFNADPARNTVFSVHMYGVFD.TAAEISDYLGRFR.SRGLPIVVGEFGHY..HSDG......DPDEDAILSYTQANGIGWLGWSWSGNSGGV
AOS63253.1	YTGWGADTSAAIGRLRAAGFDHTLVVDAPNWGQDWSGTMRSQAAGVFASDPDANTLFSIHMYGVYD.NATAVSDYLEYFV.DQGLPIMVGEFGHD..HSDG......NPDEDAILATTESLGLGYLGWSWSGNSGGV
APU14662.1	YAGWGADTSAAINRLRDAGFDHTFIVDGPNWGQDWSGTMRSQAPGVFASDPHANTVFSIHMYGVYD.TAAEIQDYLRHFV.DQGLPIMVGEFGHN..HSDG......DPDEDTIMATTEELDLGYIGWSWSGNGSPV
ANZ40233.1	ASAWTADTKSAISRLRAAGFSHTIMVDAPNWGQDWSFTMRDNAASVFAADPQRNTVFSVHMYGVFD.TAAEITDYLGRFV.AAGLPIVVGEFGHN..HSDG......DPDENTIFSYTQAQRLGYLGWSWSGNGGGV
ATE53439.1	YAAWTADTRSALTRLRAEGFTHTIMVDAPNWGQDWAFTMRDNAASVFAADPQRNTIFSIHMYGVFD.TAAEVDDYLGRFV.AAGLPIAVGEFGDM..HSDG......DPDEDAIFAAAQRLRLGYLAWSWSGNGGGV
AXB42212.1	YAGWTDATKSAIQKLRAGGLTHTLMVDAPNWGQDWSFTMRDNAASVFAADSLKNTLFSVHMYGVFD.TAAEITDYLGRFT.AAGLPILVGEFGHN..HSDG......DPDEDTIFATTQAQRLGYLGWSWSGNGGGV
AXG81675.1	YAGWTADTKAAIVKLRNAGFGHTLMVDAPNWGQDWSFTMRDNAASVFAADPDRNTMFSIHMYGVFN.TAAKVSAYLNTFV.NAGLPILVGEFGDM..HSDG......DPDEDAIMATTQSLRLGYLGWSWSGNSGGV
QLH19869.1	ATAWTADTKAAIQKLRTAGIHHALMADAPNWGQDWSFTMRDNAASVLAADPDRNTVFSVHMYGVFD.TATEVKDYLTRFV.NAKLPLVVGEFGHN..HSDG......NPDETAIMANAQTLGVGYLGWSWSGNSGGV

Continued from previous page

```
BAM62868.1 YTRWTADTKAAVQKLRAAGFGHTLMVDAPNWGQDWAFTMRDNAASVFAADPDANTVFSIHMYGVFD.TAAEVSDYLGRFV.AAKLPVVVGEFGHD..HSDG......NPDEDAILSVTRQLGIGYLGWSWSGNGGGV
AXI85415.1 YSRWTDDTKAAIQKLRAAGFDHTLMVDAPNWGQDWAFTMRDNAASVFAADPDANTVFSIHMYGVFD.TAAEVSDYLNRFV.TAKLPIVVGEFGHD..HSDG......NPDEDAVLSVTRQLGIGYLGWSWSGNGGGV
QYX82013.1 YAGWTADTKAAIQRLRTAGFDHTLMVDAPNWGQDWAFTMRDNAASVFAADPDANTVFSIHMYGVFD.TAAEVSDYLNRFV.AAKLPLVVGEFGHD..HSDG......NPDEDAILATAQRLGIGYLGWSWSGNGGGV
AYL34687.1 YTAWTADTKAAIQKLRDAGFDHTLMVDAPNWGQDWAFTMRDNAASVFAADPDANTVFSIHMYGVFD.TAAEISDYLGRFV.SAKLPIVVGEFGHN..HSDG......NPDEDTILAVTRQLGLGYLGWSWSGNGGGV
AJP00836.1 YANWTADTKAAVQKLRNAGFDHTIMVDAPNWGQDWAFTMRDNAASVFAADPDRNTVFSIHMYGVFD.TAAEVGDYLDRFV.AAKLPVVVGEFGHD..HSDG......NPDEDAILSVTQQLGLGYLGWSWSGNGGGV
AKH86353.1 YERWTADTRGAIARLRAAGLRHTLMADAPNWGQDWTNTMRTNARAVADSDPQGDTLFSIHMYGVYD.TAAEIEDYLTSFT.EAGLPIAVGEFGHD..HSDG......NPDEDAIMRLTRELGLGYLGWSYSGNGGGV
AMW08869.1 YTAWTADTKAAIQKLRNAGFDHTIMVDAPNWGQDWAFTMRDNAASVFAADPDANTVFSIHMYGVFD.TAAEITDYLNRFV.TAKLPIVVGEFGHD..HSDG......NPDEDTILAVAQQLRLGYLGWSWSGNGGGV
AXI79531.1 TSGWTADTTAAIKKMRNAGFHHTLMVDAPNWGQDWSGTMRDNAKTVFAADADANTVFSIHMYGVYT.NASLVSNYLNSFV.SAKLPIVVGEFGNM..HSDG......DPDEDSIMSYAQSLKLGYLGWSWSGNGGGV
BCJ73223.1 TPNWTDATKSAIQRLRNAGFQHTIMVDAPNWGQDWSFTMRDNAPSVLAADTTGNTIFSVHMYGVFD.TAAEVTAYVDSFT.SRNLALCVCEFGDN..HSDG......NPDEDAIMAKTQSAGIGNMGWSWSGNGGGV
CBG75158.1 YAGWTADTKAAIQKLRAAGFDHTIMVDAPNWGQDWAFTMRDNAASVFAADPDGNTVFSIHMYGVFD.TAAEVNDYLNRFV.RAKLPIVIGEFGHE..HSDG......NPDEDAILAAAQRLGLGYLGWSWSGNGGGV
QDI72496.1 YTNWTADTKAAIQKLRGAGFDHTLMVDAPNWGQDWAFTMRDNAASVFAADPDANTVFSIHMYGVFD.TAAEISDYLGRFV.AAKLPIVVGEFGHD..HSDG......NPDEDTILAVAQQLRLGYLGWSWSGNGGGV
QEU84399.1 YANWTADTRAAIQKLRAAGFDHTLMVDAPNWGQDWAFTMRDNAATVFAADPDANTVFSIHMYGVFD.TAAEISDYLGRFV.AAKLPIVIGEFGHD..HSDG......NPDEDTILAVAQQLRLGYLGWSWSGNGGGV
QEV05093.1 YANWTADTKAAIQKLRNAGFDHTLMVDAPNWGQDWAFTMRDNAASVFAADPDANTVFSIHMYGVFD.TAAEVSDYLGRFV.SARLPIVVGEFGHD..HSDG......NPDEDAILAVTQQLGLGYLGWSWSGNGGGV
QEV23521.1 YTAWTADTKAAIQKLRNAGFDHTIMVDAPNWGQDWAFTMRDNAASVFASDPDANTIFSIHMYGVFD.TAAEITDYLNRFV.TAKLPIVVGEFGHD..HSDG......NPDEDTILAVAQQLRLGYLGWSWSGNGGGV
QEV72209.1 YSAWTADTKAAVQKLRSAGFRHTIMVDAPNWGQDWAFTMRDNAASVFAADPDANTVFSIHMYGVFD.TAAEISDYLNRFV.TAKLPIVVGEFGHN..HSDG......DPDEDTILAVTRQLGLGYLGWSWSGNGGGV
QOV33815.1 YTAWTADTKAAIQKLRTAGFRHTIMVDAPNWGQDWAFTMRDNAASVFAADPDANTIFSIHMYGVFD.TAAEISDYLNRFV.TAGLPIVVGEFGHN..HSDG......DPDEDTILAVTQQLGLGYLGWSWSGNGGGV
AGZ42057.1 AAAWTADTKSAITRLRAAGFEHSIMVDAPNWGQDWQFVMRDNAASVFAADPQRNTIFSIHMYGVFD.TAAEITDYLGRFR.TAGLPIVVGEFGHN..HSDG......NPDEDTILATAQAQGIGYLGWSWSGNGGGV
AGL18898.1 ATGWTAATKNAIARLRAAGFEHSIMVDAPNWGQDWQFVMRDNAASVLAADPQRNTIFSIHMYGVFD.TAAEITDYLGRFR.SAGLPVVVGEFGFD..HSDG......NPDEDTIMATAQRLGIGYLGWSWSGNGGGV
BAL89262.1 ASKWTADTKSAISRLRAAGFQHQIMVDAPNWGQDWQFIMRDNAASVFAADPNRNTVFSIHMYGVFD.TAAEITDYLGRFQ.TAGLPIVVGEFGFD..HSDG......NPDEDTILATAQARGIGYLGWSWSGNGGGV
BBH69472.1 ATSWTADTKSAITRLRSAGFQHQIMVDAPNWGQDWQFIMRDNAASVFAADPNRNTVFSIHMYGVFD.TAAEINDYVGRFQ.TAGLPLVIGEFGYN..HSDG......DPDEDTIFATAQARGIGYLGWSWSGNGGGV
BCB91458.1 SATWPTATSNAVSRLRGAGFEHLIMVDAPMWGQDWQFIMRDNAQTVFNADPQRNTVFSIHMYGVFD.TAAEIQDYLGRFR.TAGLPIVVGEFGHN..HSDG......NPDEDTIMSYTQTNGIGYIGWSWSGNGGGV
SDT52805.1 AAGWVADTKSAITRLRAAGFAHQIMVDAPNWGQDWSFTMRDNAASVFATDPNRNVVFSVHMYGVFD.TAAEITDYLGRFR.SAGLPLVIGEFGHL..HSDG......DPDEDTILATAQQYGVGYLGWSWSGNGGGV
AXE83855.1 ASRWTADTKAAIQKLRNAGFNHTLMVDAPNWGQDWSFIMRDNAASVFAADRDRNTVFSVHMYGVYN.TAAKVNDYLNRFV.AAKLPIVVGEFGHN..HSDG......NPDEDAILAAAQRLGIGYLGWSWSGNGSDV
QNP74875.1 YTRWTADTKAAIQKLRGAGFNHTLMVDAPNWGQDWAFTMRDNAASVFAADPDRNTVFSVHMYGVFN.TAAKVNDYLNRFV.AAKLPIVVGEFGHN..HSDG......NPDEDAILATTQRLGLGYLGWSWSGNGSDV
QZZ25189.1 ASRWTADTKAAIQKLRAAGLNHTLMVDAPNWGQDWSFTMRDNAASVFAADQDRNTVFSVHMYGVYN.TAAKVNDYLNRFV.AAKLPVVVGEFGDN..HSDG......NPDEDAVMAAAQRLGIGYIGWSWSGNGSDV
ACZ83938.1 TPGWTQATSAAITRLRGAGFQHLIMVDAPNWGQDWGFTMRDNAAAVFAADPQRNTVFSVHMYGVFD.TSAEITDYLQRFQ.TAGLPLVIGEFGFN..HSDG......DPDEDTIMAQAQARGVGYLGWSWSGNGGGV
ADG87649.1 S.AWTAATINAIQRMRDAGFQHLLMVDAPNWGQDWAFVMRDNAATVFDSDPLKNTVFSIHMYGVFD.TANEITSYLQAFQ.SARLPLVIGEFGHL..HSDG......DPDEDTIMAEAEARGIGYIGWSWSGNGGGV
ANZ41596.1 SATWASATSSAISRLRGAGLQHLLMADAPMWGQDWQNIMRNNAATVFNADPLRNTVFSIHMYGVYD.TAAEINAYFDAFQ.TAGLPLVVGEFGHN..HSDG......DPDEDTIMAQAQARGIGYLGWSWSGNSSDV
AQZ68439.1 S.AWTAATSNAITRMRSLGFQHMLMVDAPNWGQDWQFTMRDNAKTVAAADTQKNTVFSIHMYGVFD.TAAEITAYLDAFQ.TAGLPLVIGEFGFN..HSDG......DPDEDTIMAQAVSRGIGYIGWSWSGNGGGV
AWS40398.1 TPGWTQATSAAITRLRSNGFQHLIMADAPNWGQDWGFTMRDNAATVFAADPQRNTVFSVHMYGVYD.TAAEVTDYMQRFQ.TAGLPLVVGEFGFN..HSDG......DPDEDTIMAQAQARGIGYLGWSWSGNGGGV
QFY13598.1 SPGWAAATSAAIRKLRDNGFDHALMVDAPSWGQDWQFTMRDNAQAVFDSDPDKNTIFSIHMYGVFD.TAAEITSYMDAFR.TAGLPLVVGEFGFN..HSDG......NPDEDTIMAQAVSRGIGYLGWSWSGNGGGV
QYC45281.1 S.GWTSATSSAITRMRSLGFQHLLVVDAPNWGQDWQFTMRDNAATVAAADPQHNTVFAIHMYGVFD.TAAEITAYLDAFQ.SAGLPLIIGEFGFN..HSDG......DPDEDTIMAQAQQRALGYIGWSWSGNGGGV
BCJ67540.1 TPGWVQATSSAINRLRDAGFDHQIMVDAPNWGQDWQFTMRDNAAAVFASDPDRNTVFSIHMYGVFD.TAAEVTDYLGRFR.TAGLPIVVGEFGFN..HSDG......NPDEDTIMSYTTTHGIGYIGWSWSGNGGGV
QFZ18119.1 T.GWTQATKDAITRLRANGFEHTIMVDAPTWGQDWAFTMRDNAASVFAADPQRNTVFSIHMYGVFD.TAAEINDYLNRFV.TAKLPIVVGEFGHD..HSDG......NPDEDTIFATTQQLKLGYLAWSWSGNGGGV
UAB98931.1 VANWTTDTANAVKRLRANGFEHTIMVDAPNWGQDWTFTMRDNAATVFAADPQKNTVFSIHMYGVFD.TAAEIDDYLNRFV.AAKLPIVVGEFGFN..HSDG......NPDEDTIMATTTALKIGYLGWSWSGNGGGV
AVT37994.1 SANWPTHTRNAITRLRNAGFDHTIMVDAPMWGQDWRFIMRDNAQSVFNADPDRNTIFSIHMYGVFD.TAAEITDYLGRFR.SANLPIVVGEFGHN..HSDG......NPDEDTIMSYAQQNGIGYIGWSWSGNGGGV
QQQ79201.1 YGNWTNDTRNAISRLRSNGFTHTLMVDAPNWGQDWSNTMRANASAVFNADPQRNTVFSIHMYGVYD.TAAEVTSYLNAFV.NARLPIVIGEFGDN..HSDG......NPDEDTIMATAQSQRLGYLGWSWSGNGGGV
CCA60180.1 YSAWTQDTKDAIGRLRAAGFDHTLMADAPNWGQDWSNTMRENAPAVFAADPDRNTVFSIHMYGVYD.TAAEVEDYLGSFV.DRGLPIVVGEFGYD..HSDG......NPDEDAIMATARRLDLGYMGWSWSGNGGGV
QFZ19812.1 YGGWTNDTRNAISRLRSNGFTHTLVVDAPNWGQDWSNTMRDNASTVFNADPQRNTVFSIHMYGVYD.TASEVTAYLNAFV.GSRLPIVVGEFGDN..HSDG......NPDENAIMATAQSQRIGYLGWSWSGNGGGV
QNP65869.1 YAAWTDATKNAVVKLRNAGLDHALMVDAPNWGQDWSHTMRNNAASVFAADPDRNTVFSIHMYGVYD.TAAEVQAYLGHFV.DAGLPIVVGEFGDN..HSDG......NPDEDAIMATARSLRVGYLGWSWSGNGSGV
ADK91085.1 YDRWTSDTIAAVQKLRNAGFDHALMVDAPNWGQDWSNTMRNNASTVFNSDPDRNTIFSIHMYGVYN.TASEVQSYLNHFV.GNRLPIVVGEFGHN..HGDG......DPDENAIMATAQSLRVGYLGWSWSGNGGGV
AKZ57916.1 YSAWTGATKAAIGKLRGAGIDHALMVDAPNWGQDWSGTMRNNAASVLASDPDRNTIFSIHMYGVYD.TAAEVRDYLHAFT.DNGLPIVVGEFGDR..HSDG......NPDEDAIMATARTLGVGYIGWSWSGNGSGV
BBB03672.1 YTAWTAATKSAVAKLRGAGIHHALMVDGPNWGQDWSNTMRDNAASVLAADPDRNTIFSIHMYGVYN.TAAKVQAYLNAYT.SAQLPIVVGEFGAQ..HSDG......DPDEDAIMATAQTQGIGYLGWSWSGNGGGV
CCA60191.1 YTAWTDATKNAIGKLRNAGIENALMVDAPNWGQDWSNTMRTNAASVFASDPRRNTIFSIHMYGVYD.TATEVQNYLNHFV.TNKLPIVVGEFGDN..HSDG......NPDENAIMATTQSLRVGYLGWSWSGNGSGV
QEU70798.1 HARWTADTSAAVGRMRSAGFGHALMVDAPNWGQDWSGTMRSNAASVFASDPDRNTVFSIHMYGVYD.TAAEVRDYLGAFV.SAGLPIVVGEFGDN..HSDG......NPDEDAIMATAQSLRLGYLGWSWSGNGSGV
QGZ52610.1 YERWASDTSAAIRKLRDAGFRHAIMVDAPNWGQDYSGTMRNDAHTVFEGDPKRNTIFSIHMYGVYE.AADTVRDYLNFFV.NAGLPILIGEFGDW..HTNG......NPNEDAIMATAEELSLGYLGWSWSGNTGG.
QKW20412.1 YTGWTAATKSAIAKVRAAGIHNALMVDGPNWGQDWSGTMRDNAASVFASDADRNTIFSIHMYGVYN.TAAKVQDYLNAYT.SAGLPIVVGEFGAQ..HSDG......DPDEDAIMATAQAQGIGYLGWSWSGNGGGV
QLJ02980.1 YGAWTDATKSAIKKLRGAGIRNALMVDAPNWGQDWSGTMRDNAASVFSSDPDGNTIFSVHMYGVYN.TASKVQDYLNYFA.GHHLPLVIGEFGDQ..HTDG......DPDEDAIMSTAQSLRVGYLGWSWSGNSGGV
QCB25360.1 YTAWTDATKAAITKLRGAGIDHALMVDAPNWGQDWSGTMRSNAASVFASDPDRNTIFSIHMYGVYD.TAAEVQDYLNAFV.GNGLPIVVGEFGDQ..HSDG......NPDEDAIMATAQSLGVGYIGWSWSGNGSGV
AAA26710.2 YAAWTDATKSAIGKLRGAGLGHALMVDAPNWGQDWSGTMRSNAASVFASDPDRNTVFSIHMYGVYD.TAAEVRDYLNAFV.GNGLPIVVGEFGDQ..HSDG......NPDEDAIMATAQSLGVGYLGWSWSGNGGGV
AIJ12409.1 YAAWTDATKSAIGKLRGAGLGHALMVDAPNWGQDWSGTMRSNAASVFASDPDRNTVFSIHMYGVYD.TAAEVRDYLNAFV.GNGLPIVVGEFGDQ..HSDG......NPDEDAIMATAQSLGVGYLGWSWSGNGGGV
ABY90130.1 YAAWTDATKSAIGKLRSAGLRHALMVDAPNWGQDWSHTMRNNAASVFASDPDRNTIFSIHMYGVYD.TAAEVQDYLNHFV.NNGLPLVVGEFGDQ..HSDG......NPDEDAIMATAQSLRIGYLGWSWSGNGSGV
APY84783.1 YTAWTGATKSAIGKLRKAGLDHALMVDAPNWGQDWSGTMRDNAASVFASDPDRNTVFSVHMYGVYD.TATEVKDYLNAFV.GRKLPVVVGEFGDQ..HSDG......NPDENAIMATTQSLGIGYLGWSWSGNGGGV
QNQ32617.1 ASAWTGATKSAISKLRSAGLNHALMVDAPNWGQDWSGTMRANAASVFASDPDRNTIFSIHMYGVYD.TAAEVQDYLNHFV.DNRLPIVVGEFGDA..HSDG......NPDEGAIMATAQSLGVGYIGWSWSGNGSGV
QIS73744.1 YAAWTSDTANALARMRAAGFRHTLMVDAPNWGQDWSFTMRDHAAEVFAADPERNTVFSIHMYGVFN.TADKVRSYLDRFI.SQRLPLVVGEFGSM..HSDG......DPDEDAIMSSARQLGVGYLGWSWSGNGGGV
ADK91084.1 YEAWTADTRSAVARMRASGFGHTLMVDAPNWGQDWSHAMRDNAAQVFAADPDRNTVFSIHMYGVYD.TAAEVNDYLNRFI.SQRLPIVVGEFGDA..HSDG......DPDENAIMAAARQLGVGYLGWSWSGNGGGV
AKN71965.1 ATPWTADTKNAIGRMRAAGFEHTLMVDAPNWGQDWSFTMRDHAAEVFAADPAKNTVFSVHMYGVFN.TAAKVKDYLNRFT.SQRLPIVVGEFGHL..HSDG......DPDEDAVMSAARQLGVGYLGWSWSGNGGGV
AYN37844.1 YAAWTSDTADAVARMRAAGFRHTLMVDAPNWGQDWSFTMRDHAAEVFAADPDRNTVFSIHMYGVFN.TADKVKSYLERFT.SQHLPIVVGEFGNM..HSDG......DPDEDAIMATAQQLGVGYLGWSWSGNGGGV
CQR60862.1 VTPWTAGTKDAIGRMRAAGFEHTLMVDAPNWGQDWSFTMRDHAAEVFAADPAKNTVFSIHMYGVFN.TADKVRDYLNRFT.SQRLPIVVGEFGDM..HSDG......DPDEAAIMSTARQLRVGYLGWSWSGNGSGV
QIJ65213.1 VTPWTADTKNAIGRMRAAGFAHTLMVDAPNWGQDWSFTMRDHAAEVFAADPARNTVFSVHMYGVFN.TAEKVRDYLNRFT.SQRLPLVVGEFGDM..HSDG......DPDEAAIMSTARQLGIGYLGWSWSGNGGGV
ALC18858.1 YSAWTSGTTDAIKRLRAAGFDHTLMVDAPNWGQDWSFTMRDNAAGVFAADPDRNTVFSIHMYGVFD.TATEVRDYLNRFV.TSKLPIVVGEFGDA..HSDG......NPDEDAIMATAEQFDVGYMGWSWSGNGGGV
QNE73622.1 YAAWTADTSRAIGRMRTAGFEHTLMADAPNWGQDWSFTMRDNAPSVFAADPRRNTVFSIHMYGVFD.AAPEVSDYLNRFV.SRGLPIVVGEFGDA..HSDG......NPDEDAILATTEQLGLGYLGWSWSGNGGGV
QQQ78619.1 YTGWTQATKDAITSLRAAGFQHTIMVDAPNWGQDWAFTMRDNAASVFAADPQRNTVFSIHMYGVFD.TAAEINDYLNRFV.TAKLPIVVGEFGND..HSDG......NPDEDTIFATTQSLKLGYLAWSWSGNGGGV
AIS01618.1 HSAWTGDTKTAIARLRAGGLDHTLMVDAPNWGQDWSFTMRDNAAGVFAADPDANTVFDIHMYGVYD.TAAEVRDYLSRFV.NAKLPIVVGEFGDM..HSDG......NPDEDAIMATAQSLGIGYLGWSWSGNGGGV
AYG78365.1 YTAWTAETKTAIGRLRAAGIHHALMADAPNWGQDWSFTMRDNAAQVFAADPDRNTLFDIHMYGVYD.SPAKVTDYLGRFV.AAHLPLTIGEFGNK..HSDG......DPDEDAIMAAAQTLGIGYLGWSWSGNGGGV
AKG46315.1 PEGWTAPTIAAVEKLRNAGFEHTIMVDAPNWGQDWQGVMRATAQSVYEADTTGNLIFSIHMYSVYD.TAQKITDYLGSFV.QAGLPLVIGEFGGP.PDEWG......DPDEDAMLAAAEQYRLGYLAWSWSGNTD..
QKV71638.1 PAGWTAPTIAAIQKLRDAGFAHTIMIDAPNWGQDWEGVMRATAQSVYEADPTGNLIFSIHMYSVYD.TAEKITDYLESFV.RAGLPLVIGEFGGP.PDEWG......DPDEDAMLAAAEQYRLGYLAWSWSGNTD..
AQA16021.1 PAGWTAPTVAAVQKLRNAGFQHTIMVDAPNWGQDWQNVMRTNARTVYDADSTGNLIFSIHMYSVYD.TAQEITDYLNAFV.DAELPILIGEFGGP.PDQYG......DPDENTMMATAQQLKLGYLAWSWSGNTD..
ADI12268.1 PAGWTAPTTAAIQKLRNAGFQHTIMVDAPNWGQDWQNVMRDNAQTVYKADTTGNLIFSIHMYSVYD.TAQEITDYLNAFV.SARLPILIGEFGGP.PDQYG......DPDENTMMAAAQQLKLGYLAWSWSGNTD..
AGP60361.1 PAGWTAPTVAAVQKLRNAGFQHTIMVDAPNWGQDLQNVMRTNARTVYDADSTGNLIFSIHMYSVYD.TAQEITDYLNAFV.SAGLPILIGEFGGP.ADQWG......DPDENTMMATAQRLKLGYLAWSWSGNTD..
AQW47637.1 PTGWTAPTVAAVQKLRNAGFQHTIMVDAPNWGQDLQNVMRTNARTVYDADSTGNLIFSIHMYSVYD.TAQEITDYLDAFV.AARLPILIGEFGGP.ADQWG......DPDENTMMAAAQRLKLGYLAWSWSGNTD..
AYC43001.1 PEGWTAPTIAAIQKLRNAGLQHTIMVDAPNWGQDWQQVMRTNARSVYAADTTGNLIFSIHMYSVYD.TAAEVTDYLNAFG.DAKLPILIGEFGGP.ADQWG......DPDEDTMMATAQQLKLGYLAWSWSGNTD..
AEN10237.1 PAGWTAPTTAAIQKLRAAGFAHTIMVDAPNWGQDWEGVMRADARSVYDADPTGNLIFSIHMYSVYD.TAAKVTDYLNAFV.DAGLPLLIGEFGGP.ADQYG......DPDEDTMMATAEELGLGYLAWSWSGNTD..
AGJ55179.1 PAGWTAPTTAAVKKLRAAGFTHTIMVDAPNWGQDWQGVMRANAQSVYDADTTGNLIFSIHMYSVYN.TAKAVTDYLNAFV.DAGLPLLIGEFGGP.ADQWG......DPDEDTMMATAEQLDLGYLAWSWSGNTD..
AWL38642.1 PSGWTAPTTAAIQKLRSAGFAHTIMVDAPNWGQDWEGVMRANARSVYDADTTGNLIFSIHIYSVYN.TADKVTDYLHAFV.DAGLPLLIGEFGGP.ADQYG......DPDEDTMMATAEELGLGYLAWSWSGNTD..
QEU74297.1 PAGWTAPTTAAIQKLRNAGFEHTIMVDAPNWGQDWQGVMRANAQAVYDADTTGNLIFSIHMYSVYN.TAEKVTDYLNAFV.DAGLPLLIGEFGGP.GDQWG......DPDEDTMMATAEELGLGYLAWSWSGNTD..
AQS65900.1 PAGWTEPTIAAVEKLRAAGFQHTIMVDAPNWGQDWQGVMRANARSVYDADPTGNLIFSIHMYSVYD.TAQEITDYLNAFV.DAGLPLLIGEFGGP.ADQWG......DPDEDTMMATAEQLGLGYLAWSWSGNTD..
AIJ17911.1 PAGWTEPTVAAVKKLRAAGLQHTIMVDAPNWGQDWQGVMRANARSVYDADPTGNLIFSIHMYSVFD.TAQEITDYLNAFV.DAELPILIGEFGGP.ADQYG......DPDEDTMMATAEQLRLGYLAWSWSGNTD..
AIV32327.1 PAGWTEPTTAAVEKLRAAGLRHTIMVDAPNWGQDWQGVMRANARTVYDADPTGNLVFSIHMYSVFD.TAQEITDYLNAFV.DAKLPILIGEFGGP.ADQYG......DPDEDTMMATAEQLGLGYLAWSWSGNTD..
```

Continued from previous page

```
AIS01044.1  PQGWTEPTIAAVKKLRAAGFQHTIMVDAPNWGQDWQGVMRADARSVYAADPTGNLVFSIHMYSVYD.TAQEITDYLNAFV.TAKLPLVIGEFGGP.ADQWG......DPDEDTMMATAERLGLGYLAWSWSGNTD..
AKZ60014.1  PAGWTAPTVAAVEKLRAAGFQHTIMVDAPNWGQDWQGVMKANARTVYDADTTGNLIFSIHMYSVYD.TAQEITDYLNAFV.DAGLPLVIGEFGGP.ADQWG......DPDEDTMMAAAERLDLGYLAWSWSGNTD..
ALC19130.1  PAGWTAPTTAAVKKLRSAGFEHTIMVDAPNWGQDWQGVMRANARSVYDADPTGNLIFSIHMYSVFD.TAQEITDYLNAFV.DAGLPILIGEFGGP.ADQWG......DPDEDTMMAAAEQLRLGYLAWSWSGNTD..
ARQ67834.1  TSGWTADTRAAIQKLRAAGFDHTIMVDAPNWGQDWSGTMRANARSLFDADPDRNVIFSVHMYGVYD.TAAEIRDYLNAFV.SAGLPILVGEFGHN..HSDG......DPDEDTIFATTQQLGLGYLGWSWSGNGGGV
AWT46021.1  PAGWTDPTIAAIKKLRAAGLQHTLMVDAPNWGQDSQGVMRTNARSVYAADPTGNLLFSIHMYSVYN.TAQKITDYLNAFV.TAKLPLVIGEFGGP.ADQWG......DPDEDTMMAAAQQLRLGYLAWSWSGNTD..
BAK26781.1  PAGWTAPTIAAVKKLRAAGLAHTLMIDAPNWGQDWQGVMRADARSVYEADPTGNLLFSIHMYSVFD.TAAEIDDYLEAFV.DAGLPLVIGEFGGP.PDQWG......DPDEDTMLAAAERLRLGYLAWSWSGNTD..
QCB26303.1  PAGWTEPTVAAVKKLRDAGFQHTIMVDAPNWGQDWQGVMRADARTVLDADATGNLIFSIHMYSVYD.TAQEITDYLNAYV.DAGLPILIGEFGGP.ADQWG......DPDEDTMMATAEQLDLGYLAWSWSGNTD..
QEU63996.1  PEGWTDPTVAAVKKLRNAGFQHTIMVDAPNWGQDWQGVMRANARTVYDADPTGNLIFSIHMYSVFD.TAAEITDYLNAFV.SAGLPLLIGEFGGP.ADQWG......DPDEDTMMAAAERLDLGYLAWSWSGNTD..
QFQ95700.1  PEGWTAPTKAAVQKLRAAGFQHTIMVDAPNWGQDWQGVMRANARSVYEADTTGNLIFSIHMYSVFD.TAAEITDYLNAFV.NAGLPLLIGEFGGP.ADQWG......DPDEDTMMATAEQLDLGYLAWSWSGNTD..
QFX79702.1  PAGWTEPTVAAIGKLRAAGLRHTVMVDAPNWGQDWQGVMRANAQSVYDADPTGNLIFSIHMYSVFD.TAQEITDYLNAFV.DARLPLLIGEFGGP.ADQYG......DPDEDTMMATAEQLGLGYLAWSWSGNTD..
QIS75621.1  PAGWTEPTIAAVKKLRAAGFQHTIMVDAPNWGQDWQGVMRANARTVYDADTTGNLLFSIHMYSVFD.TAQEITDYLNAFV.TAKLPIVIGEFGGP.ADQYG......DPDEDTMMATAEQLRLGYLAWSWSGNTD..
QNP67578.1  PAGWTEPTIAAVKKLRTAGFQHTIMVDAPNWGQDWQGVMKANAQAVYDADTTGNLIFSIHMYSVFD.TAQEINDYLDGFV.ASGLPILIGEFGGP.GDQWG......DPDEDTMMAAAERLDLGYLAWSWSGNTD..
QQM46492.1  PAGWTDPTIAAIKKLRNAGFEHTIMVDAPNWGQDWQGVMRANAQSVYDADTTGNLIFSIHMYSVYD.TAQEITDYLNAFV.NAGLPILIGEFGGP.PDQWG......DPDENTMMATAQQLGLGYLAWSWSGNTD..
QWB27515.1  PAGWTNPTIAAVKKLRNAGFAHTIMVDAPNWGQDWQGVMRANAKSVYDADPTGNLIFSIHMYSVYD.TAQEITDYLNAFV.NAKLPILIGEFGGP.ADQYG......DPDEDTMMAVAQQLRLGYLAWSWSGNTD..
QJT06651.1  PAGWTDPTIAAIKKLRNAGFQHTIMVDAPNWGQDWQGVMRANAQSVYAADTTGNLIFSIHMYSVFD.TAAEITDYLNAFV.DARLPILIGEFGGP.ADQWG......DPDEDTMMATAQQLDLGYLAWSWSGNTD..
AZS88981.1  PAGWTAPTIAAVKKLRAAGFAHTIMVDAPNWGQDLQGVMRANAQAVYAADTTGNLIFSIHMYSVYD.TAAEITDYLNAFV.NAKLPILIGEFGGP.ADQWG......DPDEDTMMATAEQLRLGYLAWSWSGNTD..
CCK29425.1  PAGWTAPTIAAVQKLRSAGLEHTIMVDAPNWGQDWQNVMRTNAQSVYAADPTGNLIFSVHMYSVYD.TAAEITDYLNAFV.AARLPILIGEFGGP.ADQWG......DPDEDTMMAAAQNLDLGYLAWSWSGNTD..
QIJ66767.1  PQGWTEPTIAAIKKLRAAGFQHTIMVDAPNWGQDWQGVMRANAQSVYAADPTGNLIFSIHMYSVYD.TAQEITDYLNAFV.NAKLPIVIGEFGGP.PDQWG......DPDEDTMMAAAEQLDLGYLAWSWSGNTD..
QZZ26035.1  PAGWTAPTIAAIQKLRNAGFQHLLMVDAPNWGQDWQGVMRANAQSVYAADTTGNLIFSIHMYSVFD.TAAEITDYLNAFV.TAKLPLLIGEFGGP.ADQYG......DPDEDTMMATAQQLKLGYLAWSWSGNTD..
SHI71357.1  PEGWTAPTIAAIKKLRNAGFDHTIMVDAPNWGQDLQGVMRANAQSVYAADPTGNLIFSIHMYSVYD.TAAEITDYLNAFV.NAKLPILIGEFGGP.ADQWG......DPDEDTMMAAAQQLKLGYLAWSWSGNTD..
ANS63452.1  PDGWTAPTIAAIQKLRTAGFQHTIMVDAPNWGQDWQGVMRANAQSVYDADTTGNLIFSIHMYSVFD.TAAEITDYLNAFV.NAKLPLLIGEFGGP.ADQWG......DPDEDTMMATAEQLDLGYLAWSWSGNTD..
ALV37404.1  PNGWTAPTIAAVKKLRAAGLQHTIMVDAPNWGQDWQGVMRANAKSVKDADSTGNLIFSIHMYSVYD.TAQEITDYLNAYV.NAKLPIVIGEFGGP.ADQYG......DPDEDTMMATAQQLKLGYLAWSWSGNTD..
AVV46344.1  PAGWTAPTIAAVKKLRAAGFQHTIMVDAPNWGQDWQGVMKANARSVYDADTTGNLIFSIHMYSVFD.TAAEVTDYLNTFV.NAKLPLLIGEFGGP.ADQYG......DPDEDTMMAAAQRLELGYLAWSWSGNTD..
AZP15527.1  PEGWTAPTIAAIKKLRTAGFEHTIMVDAPNWGQDWQGVMRANAQSVYNADTTGNLIFSIHMYSVFD.TAAEITDYLNAFV.NAKLPILIGEFGGP.ADQWG......DPDEDTMMATAERLDLGYLAWSWSGNTD..
AZQ39351.1  PVGWTDPTIAAIKKLRSAGFAHTIMVDAPNWGQDWQGVMRDNARSVYAADPTGNLIFSIHMYSVYD.TAQEITGYLNTFV.NAQLPILIGEFGGP.PDQYG......DPDEDTMMAAAQQLRLGYLAWSWSGNTD..
CBG74852.1  PAGWTAPTKAAVRKLRAAGLEHTIMVDAPNWGQDWQGVMRADARSVYEADTTGNLVFSIHMYSVFD.TAAEINDYLNAFV.DARLPLLIGEFGGP.ADQWG......DPDEDTMMAAAERLDLGYLAWSWSGNTD..
QUC60361.1  PDGWTAPTIAAIQKLRGAGFQHTIMVDAPNWGQDWQQVMRTNARSVYAADTTGNLVFSIHMYSVFD.TAAEITDYLNAFV.GAKLPIVIGEFGGP.ADQWG......DPDEDTMMATAEQLDIGYLAWSWSGNTD..
QKW47771.1  PAGWTAPTTAAIQKLRGAGFAHTIMVDAPNWGQDWQGVMRANAQAVYEADMTGNLIFSIHMYSVYS.TPQAITDYLQAFV.NAGLPLLIGEFGGP.ADQWG......DPDEDTMMATAEALDLGYLAWSWSGNTD..
QXQ95506.1  PAGWTAPTTAAVQKLRGAGFAHTIMVDAPNWGQDWQGVMRAGAQAVYDADTTGNLVFSIHMYSVYS.TPQAITDYLQAFV.DAGLPLVIGEFGGP.ADQWG......DPDEDTMMATAEALDLGYLAWSWSGNTD..
AYY15149.1  NANWAADTADAIGRLRDAGFDHTLVVDAPNWGQDWQQIMLDEAADVFAADPHANTVFSIHMYGVYE.TETAVEDYLTTFV.DAGLPLMIGEFGHQ..HSDG......DPAEDAIMAHAEELGLGYVGWSWSGNSDEV
QUX25221.1  VANWASDTSAAIGRLRDAGFHHTLVADAPNWGQDWSHTMRDGAASVFASDPHANTLFSIHMYGVYA.QESTVVSYLEHFT.NAGLPLIVGEFGHD..HSDG......NPDEDAILEHTRRLGLGYLGWSWSGNGGGV
APC36031.1  NQNWAADTSDAVNRLRDAGFDHTLVVDAPNWGQDWAGVMRDNAQSVFDSDPHANTVFSVHMYGVYQ.QESTVVSYLEHFV.DAGLPLIVGEFGHD..HSDG......NPDEDAIMSHAERLGLGYIGWSWSGNGSPV
QBI51874.1  YESWAPDTTDAVNRLRDAGFEHTIMVDAPNWGQDWTNTMRDNAAQVFESDPDRNLLFDIHMYGVYE.QASRIQDYLGYYV.GNGLPIAVGEFGHN..HSDG......DPDEDAILATAERLDIGYMGWSYSGNSGGV
QKW32020.1  NQGWADDTIAAVGRLRDAGFEHTLVVDAPNWGQDWQGIMRRDAPLVAAADD..NTVFSIHMYGVYQ.QESTVVSYLEHFA.NAGLPLIVGEFGHD..HSDG......DPDEDAIMEHTRRLGLGYIGWSWSGNGGGV
QVJ00172.1  NASWASATSEAIQRLRAAGFDHTLVVDAPNWGQDWTHTMRDGAQGVFDADPHANTVFSIHMYGVYE.QESVIVDYLESFT.DRGLPLVVGEFGFD..HSDG......DPDEDAIMRHTERLGLGYVGWSWSGNSGGV
AAZ54938.1  VARWASDTSAAIQRLRAAGFEHTLVVDAPNWGQDWTNTMRNNADQVYASDPTGNTVFSIHMYGVYS.QASTITSYLEHFV.NAGLPLIIGEFGHD..HSDG......NPDEDTIMAEAERLKLGYIGWSWSGNGGGV
AHB89702.1  VARWASDTSAAIQRLRAAGFEHTLVVDAPNWGQDWTNTMRNNADQVYASDPTGNTVFSIHMYGVYS.QASTITSYLEHFV.NAGLPLIIGEFGHD..HSDG......NPDEDTIMAEAERLKLGYIGWSWSGNGGGV
AID15578.1  VARWASDTSAAIQRLRAAGFEHTLVVDAPNWGQDWTNTMRNNADQVYASDPTGNTVFSIHMYGVYS.QASTIASYLEHFV.NAGLPLIIGEFGHD..HSDG......NPDEDTIMAEAERLKLGYIGWSWSGNGGGV
AHB89703.1  VANWASDTSAAIRRLRAAGFEHAIVVDAPNWGQDWSHTMRTNAADVYASDPTGNTIFSIHMYGVYS.QGSTVTSYLEHFV.NAGLPLVIGEFGHD..HSDG......NPDEDTIMAEAERLGLGYIGWSWSGNGGGV
AHB89704.1  VADWASDTSAAIQRMRDAGFDHTLVVDAPNWGQDWSHTMRDNAGSVYTSDPTGNTVFSIHMYGVYE.QGSTVTSYLEHFV.NAGLPIMIGEFGHD..HSDG......NPDEDTIMAEAERLGLGYIGWSWSGNGGGV
BBA57841.1  VADWASDTSAAIQRLRSEGFDHTLVVDAPNWGQDWSHTMRDNAAGVYASDPTGNTVFSIHMYGVYS.QGATVTSYLEHFV.NAGLPIMIGEFGHD..HSDG......NPDEDTIMAEAERLRLGYIGWSWSGNGGGV
PDB         VAAWATDTSAAIQRLRAAGFEHTLVVDAPNWGQDWTNTMRNNADQVYASDPTGNTVFSIHMYGVYS.QASTITSYLEHFV.NAGLPLIIGEFGHD..HSDG......NPDEDTIMAEAERLKLGYIGWSWSGNGGGV
ATB37764.1  YTEWVGATINAIQRLRNAGLTHTLMIDAPNWGQDWSNTMRDNADTIWAADPLRNSIFSVHMYGVYN.TPEKVKAYLDSFT.SRGLPILVGEFGWY..HSDG......DPDEFTLTEYTTLQGLGYMGWSWSGNGGGV
QRK07611.1  PTAWVAAIIDAIQRLRAAGLTHTLMIDGPNWGQDWSNTMRDNAQTIWEADPLRNSIFSIHMYGVYN.TPSKVKAYLDSFT.SRGLPILVGEFGWY..HSDG......DPDEVTITEHTTSLGLGYIGWSWSGNGGGV
UAC00364.1  TSGWARDTSAAIAALRAAGVRHLLMADAPNWGQDQSKTMRQHAAAVLAQDPQHNTVFSVHMYGVYD.TAAKVTDYLDAFR.TAGLPLVVGEFGQR..HTDG......PVAADAIMRETRTRGIGYLGWSWSGNGGGV
BAL87525.1  PGQWTAATVDAVKKMRSNGFQHLLMVDAPNWGQDWQYTMRNNAQTVLDADVQHNLILSIHMYAVFN.TAASIVDYLNTFK.NNGWPLIIGEFGWR..FDSS......QVDHETLLAEAAARDLGYLGWSWAGNTD..
BBH67620.1  PGQWTAATVAAIQKMRTNGFQHLLMVDAPNWGQDWQYTMRDNAQTVLNADTQKNTVLSIHMYAVFN.TAASIVDYLDTFE.NNGWPLVIGEFGWR..FDSS......QVDHETLLAEAVKRDLGYLGWSWAGNTD..
SDT42643.1  PGQWTAATVAAIQKMRTNGFEHLLMIDAPNWGQDWQYTMRDNAQTVLNADTRANTVLSIHMYAVFN.TAASIRNYLDVFQ.NNNWPLVIGEFGWQ..FASG......EVDDTTLMAEAQARGIGYLGWSWAGNTD..
BCJ48281.1  PSQWTAATTAAIKKMRDNGFQHLLMIDAPNWGQDWQQVMLANGQTVLDADSQHNTILSIHMYSVYS.QASTITSYLDTFK.SKGWPLLIGEFGWQ..FASG......EVDDQTVIAEAARRGLGYLGWSWSGNTD..
BCY10256.1  PGQWTAATVAAVKKMRDNGFQHLLVIDAPNWGQDWQYVMRDNGQAVLDADAQHNTVLSIHMYSIFA.QASSITSYLDTFK.SKGWPLIIGEFGWQ..FDSS......QVDDQTAIAEAVKRDIGYIGWSWSGNTD..
BCJ76695.1  ASQWTAATVAAIQKMRTNGFQHLIMVDAPNWGQDWQYTMRDNAQTILNADTQRNTVLSIHMYAVFN.TATAITDYLNRFR.TNGWPLVIGEFGWR..FNSN......EVDHETILAEAQARGLGYLAWSWSGNTD..
UAB94120.1  PSQWTAATVAAIQKMRSNGFQHLLMVDAPNWGQDWQNVMRDNAQQVYDADTLKNTVLSIHMYAVYA.NAQTIIDYLDRFQ.NNNWPLVIGEFGWQ..FQSG......EVDHETLLAQADARGLGYIGWSWSGNTD..
AVT28826.1  AGQWTAATASAIARMRSNGFQHLLMVDGPNWGQDWQYVMRDNAQTILDADTQRNTVLSIHMYAVFN.TAASITDYLNRFQ.ANGWPLVIGEFGWR..FNSG......EVDHETILAEANARGLGYLGWSWSGNTD..
ACU38007.1  AAQWTDATVNAVKKMRTNGFQHLLMVDGPNWGQDWQYVMRDNAQRVLDADTQRNTVLSIHMYAVFS.TPASIIDYLDRFQ.ANGWPLVIGEFGWK..FASG......EVDHETILAQAQARGMGYLGWSWSGNTD..
BBB03671.1  AAQWTAATANAIKKMRSNGFEHLLMVDAPNWGQDWQYVMRDNAQTILNADTQHNTVLSIHMYAVFN.TAAGIVDYLNRFQ.ANGWPLVIGEFGWR..FSSS......EVDHETILAEAQSRGLGYLGWSWSGNTD..
BCB90671.1  AGQWTAATVAAIQKMRTNGFEHVLMVDAPNWGQDWQYTMRDNAQTVLNADSQRNTVLSIHMYAVFN.TAASITDYLNRFQ.ANGWPLVVGEFGWR..FNSG......EVDHDTILAEAQARGIGYLGWSWSGNTD..
BCJ68966.1  AAQWTAATAAAIGRMRDNGFAHLLMVDGPNWGQDWQQVMRNNAQTILDADEQHNTVLSIHMYAVYA.TAASIVDYLDHFE.ANGWPLVIGEFGWR..FNAG......EVDHETLLAEADARDLGYLGWSWSGNTD..
BCL17300.1  AAQWTDATVAAIRKMRANGFAHLLMVDGPNWGQDWQHVMRDNARTVLDADSQHNTVLSIHMYAVFN.TAASIVDYLDRFE.ANGWPLVIGEFGWR..FRDG......EVDHEALLAEADARGLGYLGWSWSGNTD..
QFY13395.1  AAQWIDATANAIRKLRGNGFEHLIMVDAPNWGQDWQYVMRDNAQTILDADTRHNTVLSIHMYSVFN.TASSIVSYLDTFR.TKGWPLVIGEFGWQ..FNSG......EVDHETILSEAQSRGLGWLAWSWSGNSD..
QFZ19526.1  AAQWTDATVNAVKKMRTNGFQHLLMVDAPNWGQDWQYVMRDNAQKVLDADTQRNTVLSIHMYSVFN.TASAITDYLDRFQ.ANGWPLVIGEFGWQ..FAAG......EVDHETILSEAQQRGLGYLAWSWSGNTD..
QQQ79259.1  AAQWTDATAGAIKKLRANGFRHLLMVDAPNWGQDWQHVMRDNAQEVLDADTARNTVLSVHMYSVFN.TASSITDYLDRFQ.ANGWPLVIGEFGWR..FAAG......EVDHETILSESQKRGLGYLAWSWSGNSD..
SBT42754.1  ASQWTSATTAAIKKLRANGFEHLLMVDGPNWGQDWQFVMRDNAQAILDADNQHNTVLSIHMYAVFN.TAAAVTDYLNRFQ.TNGWPLVIGEFGWR..FNAG......EVDHETILAEAQARRLGYLGWSWSGNSD..
SCF29942.1  AAQWTDATVAAIRKMRANGFAHLLMVDGPNWGQDWQNVMRDNAQAVLDADSQRNTVLSIHMYAVYS.TAASIVSYLDRFE.ANGWPLVIGEFGWR..FRDG......EVDHETLLAEADARRLGYLGWSWSGNTD..
QIL90192.1  EINYVEGTTAAIQRIRDAGLVHTIVVDASSWGQDWENYMRANAPQIFAADPLANVLFSVHMYQVYA.DHSTIASYMQDFQ.QMGLPLIVGEFGHE..HQGQ......PVDEDAILAEAEARGIGYLGWSWSGNTE..
AMX03424.1  ESAYVEDTSAAIQTLREAGLTHTLVVDAANWGQDWQGYLRDNAAQIFAADPLANTLFSVHMYQVYP.DRGTVADYMQAFE.DMQLPLMVGEFAYE..HQGQ......PVDEDAILAEAEARGIGYLGWSWSGNTE..
QEY11805.1  AATYTADHITAIRALRAGGLTHMLMVDAANWGQDWQNAMRDNAPTILAADTLQNVVFSVHMYEVYN.NAAIVQSYLNSFQ.TRGLAIVVGEFGAE..HNGS......NVAEEAILQYAQDMGIGYLGWSWSGNGS..
QIL90886.1  ESTYVNDTKGAIQALRNGGITHTIMVDAGNWGQDWQFFMRNNAPDIFSSDPARNVIFDVHMYEVFS.DASKVSSYMQAFA.NHNLPLVVGEFGAT..HGGG......NVDEDSIFSYAQQLGFGYLGWSWSGNGS..
QZD54573.1  Q.QWTQATVNAVDRMRDIGFEHTLVVDAPNWGQDWTNTMRDNAPTVAAADPLGNTLFSVHMYQVYG.NAQTVIDYFDAFE.AMNLPLIVGEYGDV..HQGE......PVAWQTVQSEAQARGIGWIAWSYSGNGGG.
ARU50407.1  ADEWAAQTSAAIQRLRDIGYEHTIVVDAPNWGQDWSRTMYSQAQQVFDADPDANVVFSVHMYEVYG.DGAVVTDYLEHFV.DAGLPLIVGEFGPA..HYGN......DVDEDTILAETRRLGLGWFGWSWSGNSGGV
ANC31527.1  NETWAAETSAAIQRMRSIGYEHALVVDAPNWGQDWKNYMRPAAAGVFAADPDANTVFSIHMYSVYN.QPQTITDYLEFFV.DNGLPIIVGEFGWQ..QTPA......DVDEDVVLSEAERLDLGWLAWSWSGNTD..
ARU52036.1  NQRWAPETSAAIQRLRGIGYEHAIVVDAPNWGQDWTNVMRAEAQGVFDADPDANTIFSIHMYGVYA.QAATVTDYLEDFV.DAGLPIMVGEFGWK..RAEG......DVDADVVIAEANRLDLGWLAWSWSGNTD..
AAA71887.1  YQNWVNDTKNAIKALRDAGFKHTIMVDAPNWGQDWSNTMRDNAQSIMEADPLRNLVFSIHMYGVYN.TASKVEEYIKSFV.DKGLPLVIGEFGHQ..HTDG......DPDEEAIVRYAKQYKIGLFSWSWCGNSSY.
ACM60947.1  YQNWINDTKNAIKALRDAGFKHTIMVDAPNWGQDWSNTMRDNAQSIMEADPLRNLVFSIHMYGVYN.TASKVEEYIKSFV.EKGLPLVIGEFGHQ..HTDG......DPDEEAIVRYAKQYKIGLFSWSWCGNSSY.
ACM60953.1  YQNWINDTKNAIKALRDAGFKHTIMVDAPNWGQDWSNTMRDNAQSIMEADPLRNLVFSIHMYGVYN.TASKVEEYIKSFV.EKGLPLVIGEFGHQ..HTDG......DPDEEAIVRYAKQYKIGLFSWSWCGNSSY.
ACM60954.1  YQNWINDTKNAIKALRDAGFKHTIMVDAPNWGQDWSNTMRDNAQSIMEADPLRNLVFSIHMYGVYN.TASKVEEYIKSFV.EKGLPLVIGEFGHQ..HTDG......DPDEEAIVRYAKQYKIGLFSWSWCGNSSY.
ADL42950.1  YQNWVNDTKNAIKALRDAGFKHTIMVDAPNWGQDWSNTMRDNAQSIMEADPLRNLVFSIHMYGVYN.TASKVEEYIKSFV.EKGLPLVIGEFGHQ..HTDG......DPDEEAIVRYAKQYKIGLFSWSWCGNSSY.
ADQ45728.1  YQNWINDTKNAIKALRDAGFKHTIMVDAPNWGQDWSNTMRDNAQSIMEADPLRNLVFSIHMYGVYN.TASKVEEYIKSFV.EKGLPLVIGEFGHQ..HTDG......DPDEETIVRYAKQYKIGLFSWSWCGNSSY.
ADQ45730.1  YQNWINDTKNAIKALRDAGFKHTIMVDAPNWGQDWSNTMRDNAQSIMEADPLRNLVFSIHMYGVYN.TASKVEEYIKSFV.EKGLPLVIGEFGHQ..HTDG......DPDEEAIVRYAKQYKIGLFSWSWCGNSSY.
AZT90847.1  YQNWVNDTKNAIKALRDAGFKHMIMVDAPNWGQDWSNTMRDNAQSIMEADPLRNLVFSIHMYGVYN.TASKVEEYIKSFV.DKGLPLVIGEFGHQ..HTDG......DPDEEAIVRYAKQYKIGLFSWSWCGNSSY.
```

Continued from previous page

```
AEE96309.1  YQNWTQDTIDAINALREAGFNHTIMVDAPNWGQDWSFTMRDNAPKVYAADPNKNMVFSIHMYGVYN.TAQKVHDYFQAFK.DMNLPLVVGEFGNQ..HSDG......DPDEEAIVSYAKQYGIGYLGWSWCGNSGGV
AAF22274.1  YQNWVTDTRNAVQALRNAGINNTIMVDAPNWGQDWSFTMRDNAPTIFNADPQRNLVFSIHMYGVYD.TAAEVQSYIESFV.NRGLPLVIGEFGHM..HSDG......DPNEQAIVQYAKQYNIGLFGWSWSGNGGGV
ADN02189.1  YQNWVQDTIDAVQALRNAGFQHTIVVDAPNWGQDWSFTMRDNAPQVYAADPQQNLVFSVHMYGVYD.TAQEVQDYFQAFA.DMGLPLIVGEFGYM..HSDG......DPNEEAIVSYARQYGIGYWGWSWCGNGGGV
QCB92788.1  SAGWAADTSAAIRTIRAAGLRHNIVVDAPMWGQDWGGIMRDQAPTVAAADPDGNVLFSVHMYGVYA.QASTITSYLDAFA.AHGLPLVIGEFGFD..HSDG......NPDEDTIMAEAQRRGIGYYGWSWSGNGGGV
QGQ18559.1  SAQWAADTSAAIRTIRAAGLRHNIVVDAPMWGQDWRGIMRDQAPTVAAADPDGNVLFSVHMYGVYA.QASTITAYLDAFA.AHGLPLVIGEFGHD..HSDG......NPDEDTIMAEAQRRGIGYYGWSWSGNGGGV
QVI61610.1  SAGWAADTAAAVKKLRAAGLRHNIVVDAPMWGQDWKGIMRDQAQTVAAADPDGNTLFSVHMYGVYA.QASTITSYLDAFE.AKGLPLVIGEFGYD..HSDG......NPDEATIMREAVERGIGYYGWSWSGNGGGV
QWC15390.1  VTAWTADTAAAVVKVRAAGLRHNIVVDAPNWGQDWKGVMRDTAATVAAADPERNTLFSVHMYGVYA.QASTITAYLDAFA.AQGLPLVIGEFGYD..HSDG......NPDEATIMSEAVRRGIGYYGWSWSGNGGGV
ADG75696.1  VTGWTVDTAAAIRKVRAAGLRHNIVVDAPNWGQDWKGVMRDTAATVAAADPDGNTLFSVHMYGVYS.QASTITSYLDAFE.AKGLPLVIGEFGID..HSDG......DPDEATIMREATERGIGYYGWSWSGNSGGV
ASR55418.1  NATWTADTVAAIKKLRAAGLHHNIVVDAPSWGQDWAGIMRDNAQTVAAGDPDGNVLFSVHMYGVYN.NASTIRTYLDAFK.AKGLPLVIGEFGDM..HSDG......DPDEDTIMSEAAARGIGYYGWSWSGNGGGV
AEI13136.1  NAGWAADSVAAIAKLRAAGLHHNIVIDAPSWGQDWAGIMRDQAQTVAAGDPDGNVLFSVHMYGVYN.NATTIRSYLDAFK.AKGLPLVIGEFGFD..HSDG......NPDEDTIMAEAVARGLGYYGWSWSGNGGGV
QHT56776.1  NAGWAADTSAAIKKLRAAGLHHNIVVDAPSWGQDWAGIMRDQAATVAAADPDANTLFSVHMYGVYN.NASTIKTYLDAFK.AKNLPLVIGEFGFD..HSDG......NPDEDTIMSEAVARGIGYYGWSWSGNGGGV
QIK83955.1  YQSWASDTISAISTLRHAGLRHTIVVDAPNWGQDWSGTMKADAPRVATA..DGNTVFSIHMYGVYD.TAAEVTSYIDSFR.SRGLALMVGEFGDA..HSDG......NPDEATIMRYTRTTGVGMLGWSWSGNGSGV
QVI65258.1  NATWASASSAAIAKLRAAGLHHNIVIDAPSWGQDWAGIMRDNAATVAASDPDKNILFSVHMYGVYN.NASTINAYLDAFA.SKGLPLVIGEFGNM..HSDG......DPDEDTIMAEAVERGIGYYGWSWSGNGGGV
ACZ29543.1  NERWADDTIAAIQRLRTAGFEHTILVDAPNWGQDWAGIMKRDAAQVAAADPQGDVVFSVHMYGVYD.TAAEVRDYLDAFI.AAGLPLVVGEFGFD..HSDG......NPDEDTIMSYTRAQGIGWFAWSWSGNGGGV
AEE43708.1  FQSWTTDTIAAIRTLRAAGLDHTLVVDAPNWGQDWSFTMRDNAPTVAAAD..GNVVFSVHMYGVFD.TGAEVRAYLDSFT.SRGLPIMVGEFGDN..HSDG......NPDEATIMSYTRSQGIGMLGWSWSGNGGGV
AQQ16388.1  FQSWTTDTIAAIQTLRGAGLAHTLVVDAPNWGQDWSFTMRDNAPTVAAAD..GNVVFSVHMYGVFD.TGAEVRAYLDSFT.SRGLPIMVGEFGDN..HSDG......NPDEATIMSYTRSQGIGMLGWSWSGNGSGV
AAO31760.1  ASTWVDQHKQAIQRIRTAGLTHTLIVDAANWGQDWEEIMLNNASSVAAADTLKNTLFSVHMYQVYQ.SRSTVESYVTRFMQTHNLPLIVGEFGAD..HYGE......FVDADSIMAVAQQYNIGYLGWSWSGNSSNV
ACE84941.1  ASTWVDQHKQAIQRIRTAGLTHTLIVDAANWGQDWEEIMLNNASSVAAADTLKNTLFSVHMYQVYQ.SRSTVESYVTRFMQTHNLPLIVGEFGAD..HYGE......FVDADSIMAVAQQYNIGYLGWSWSGNSSNV
ARU29988.1  ESSWIDGHIQAIQRIRSAGLTHTLLIDAANWGQDWQEVMLNNASTVAAADSQNNTMFSVHMYEVYQ.DRAKIEGYVSNFLSTHNLPLIVGEFGAD..HYGN......NVDEDSILAVAEQYNIGYLGWSWSGNSSEV
AIF91534.1  DSMWINDHRSAIQTLRSAGLTHTLMIDAPNWGQDWDNVMYNNASDVAAADSQNNTMFSVHMYEVYQ.DRSTIENYVSGFLSNHNLPLIVGEFGAD..HQGS......FVDADSIMAVAQEYGIGYLGWSWSGNGD..
ABD79328.1  ASNWINQHKAAIQTLRAAGLTHTLMIDAANWGQDWQQVMLNNASEVAQADSLSNTMFSVHMYQVYN.NLSTVENYVSTFLSSHNLPLIVGEFGAD..HQGE......EVDEDAILSVAEQYGIGYLGWSWSGNGS..
ACR11348.1  AAKWINEHRAAIQTIRAAGLTHTLLVDASNWGQDWEEVMLAHASEVATADSLANTMFSVHMYQVYQ.TRAKIENYVSTFLSDHNLPLIIGEFGAD..HQGE......FVDADSILAVAEQYGIGYLGWSWSGNGS..
AIF91546.1  DSMWIGDHRAAIQTLRNAGLTHTLMVDAPNWGQDWDNVMYNNASEVAAADTLGNTMFSVHMYEVYQ.DRATVENYITGFMSNHNLPIIVGEFGAD..HQGT......FVDADSIMAVSQQNGTGYLGWSWSGNGD..
AIF91556.1  SSMWINDHRSAIQTLRNAGLTHTLMVDAPNWGQDWDSVMLNNASQVASADSANNTMFSVHMYEVYQ.NRSSVENYVSSFLSSHNLPLIVGEFGAD..HQGN......AVDADSIMAVAQQYNIGYIGWSWSGNGD..
VAX65844.1  SSMWINDHISAIQKLRNAGLTHTLMIDAPNWGQDWENVMRNNAPQVAAADSLNNTMFSVHMYQVYQ.DYSTINGYLTGFVSDHNLPIIVGEFGGD..HQGD......EVDEDSILQVAQQNGIGYLGWSWSGNGD..
AJQ96022.1  SSVWIDGHKDAIKRLREAGLTHTLLIDAPAWGQDWQHVMLDNAKTVAAADSLNNTMFSVHMYQVYG.DYNSVYQYVSKFLNNNQLPLIVGEFGAD..HQGE......NVDEDSIMALAEQYGIGYLGWSWSGNSGGT
ABC27008.1  TTAWAQIHKEAIQRLRDAGFTHTLMIDAPTWGQDWRETMLQQAPEVFATDPLQNLVFSVHMYQVYA.ERSKIDNYLSTFVNVHKLPLVVGEFGAD..HQGE......EVDEAAIMELADFYRVGYLGWSWSGNSGGT
AJQ97011.1  SSVWINDHMDAIRRLRDAGLTHTLLVDAPSWGQDWERVMLNNAATVASADVLQNTVFSVHMYQIYP.DHSTVYNYITQFLENNQVPLVIGEFASV..GYQG.....ASVDVDSIMALSEEMGLGYIGWSWSGNSGSA
ABD79918.1  ASTYTNDTIAAIQKLRNAGYTHTLMVDAANWGQDWQNLMRDNAQTIFNGDSLNNTMFSVHMYQVYN.TSAKVQSYMQAFS.DKGLALVVGEFAAD..HFSE......DVAEAAIMQYAEQFGFGYMGWSWTGNSSD.
BAB79290.2  ASEWVNGHANAINRLRSAGLTHTLMVDAPNWGQDWQGLMRANAPAVLSADVDNNVVFSVHMYQVYD.TANKVQSYINGFV.SDGLPLVVGEFAAD..HFAE......DVAEGAILQTAQNAGVGYLGWSWSGNSSD.
ACR13797.1  ADDWIDAHKEAITRLRAAGLTHTLMVDAANWGQDWQYVMRDHAQEIFAHDPLANIVFSIHMYQIFN.NRQAVDSYLKTFVEDYKLPLVVGEFGAD..HGGE......DVDEASILELCELYNLGYLGWSWSGNSGGV
BAA25188.1  AVAWVNDHVSAIQRLRSAGINHTIMVDAPNWGQDWKGFMLNNAQFVFNSDPKLNTIFSVHMYEVYS.SYNSVNDYISSFT.NNGLVLVIGEFAST..HKGA......DVDEGSIMERSETLSLGYIGWSWSGNDTT.
ADT88758.1  SSDWVKAHKDAINRLRSEGMNHVIMVDAPNWGQDWQNIMLDNATSILDSDPLHNIIFSVHMYEVYG.DYSTVNNYISSFV.NKGLSLVVGEFAAT..HKGS......NVDESAIMERTETLSLGYIGWSWSGNDDST
AWB65998.1  ASSWINDHKTAITRLRNNGFTHTLMVDAPNWGQDWQNVMRDNASQVFNHDPDKNIVFSVHMYEVYD.SYNKVNNYMSTFA.NNGLPLVVGEFAAT..HKQF......DVDEASIMERAEYYDMGYLGWSWDGNDA..
QMV13106.1  ASVWINDHIDAIQRLRKAGLTHTLMVDAPNWGQDWQQIMLGHAQSVFDADPLHNTIFSVHMYQVYG.DYPAVNNYISTFL.NQGLALVVGEFGAS..HQGE......DVAEGAIMERAETLNIGYIGWSWSGNNQDV
QGY75211.1  PSEWVNGHAAAIKKMRNAGLANALMVDAPNWGQDWRFYMRDNAAVLLAKDPRKNVMFSVHMYEVFS.TAAKVDAYMRRFR.DQNLTLVVGEFAAD..HKGA......AVDEGAIMARAQAYGIGYMGWSWSGNDAS.
AAM41068.1  ASEWVNGHANAIATLRGAGLTHALMVDAPNWGQDWQFYMRDNAAALLARDSRRNLIFSVHMYEVFG.SDAVVDSYLRTFR.SNNLALVVGEFGAD..HRGA......PVDEAAIMRRAREYGVGYLGWSWSGNDSST
ACE84673.1  ASTWLNLHRDAINRLRNAGFTHTLMVDAANWGQDWENIMRNNASSLFNSDPRRNVIFSVHMYEVYP.NDTAVNNYMSAFN.SMNLPLVVGEFAAN..HFGS......YVDAGSIMARAQQYGFGYLGWSWSGNSSN.
AMU98328.1  ASEWVNGHATAIATLRKNGLTHALMVDAPNWGQDWKFYMRDNAAVLLARDSRRNLMFSVHMYEVFG.SDATINNYLRAFN.DKKLALVIGEFGGD..HRGA......HVDEAAIMRRARDYNVGYMGWSWSGNDSST
QKD86610.1  ASEWVNGHATAIATLRKNGLTHALMVDAPNWGQDWQFYMRDNAAVLLARDSRRNLMFSVHMYEVFA.SDVTINNYLRAFN.DKKLALVIGEFGGD..HRGA......RVDEAAIMRRARDYNVGYMGWSWSGNDSST
APP73913.1  ASEWVTGHARAIATLRNAGLTHALMVDAPNWGQDWQFYMRDNAAALLALDSRRNLVFSVHMYEVFG.NDATVDSYLRALR.SNGLALVVGEFGAD..HRGA......QVDEAAIMRRAREYGVGYMGWSWSGNDSST
APP81017.1  ASEWVNGHATAIAALRKSGLTHALMVDAPNWGQDWQFYMRDNAAALLARDSKRNVIFSVHMYEVFG.SDATVNNYLRAFR.DKKLTLVIGEFGGD..HRGA......NVDEASIMRRAREYSVGYMGWSWSGNDSST
AVQ07386.1  GSEWVNSHANAIATLRKNGLTHALMVDAPNWGQDWQFYMRDNAAVLLARDSRRNLIFSVHMYEVFS.SDTTVNNYLRAFS.DKKLALVIGEFGGD..HRGV......HVDESAIMRRAREYSVGYMGWSWSGNDSGT
QEY11219.1  ASAWTDGHKNAISRLRAAGFKHALMVDAANWGQDWQNIMRDNAASVLASDPQKNVIFSVHMYEVYG.SASSINSYMASFK.NNGLALVVGEFAAD..HFGS......NVDEGAIMSYAKQYNYGYFGWSWSGNGSG.
QEY19054.1  ASTYTNETIAAIKTIRNAGITHQIMVDAPAWGQDWQYVMRDNAASIFAADPLKNLVFSVHMYEVYN.TAARVQEYFSTFR.AKGYPLIVGEFGTE..NNGN......NVDEASIMQYAQDLGIGYMGWSWSGNGS..
ACV09865.1  NARYVNDTTTAITRLRNAGLTHTIVVDAPGWGQDWQHIMRDNAATILNADPLKNVVFSVHMYEVYS.SPQTVKDYINSYR.SRELPLIIGEFAST..HFGK......PVAWETILNESTNKGIGYYGWSWSGNGP..
ACV09866.1  NASYVAETKAAIATMRDAGLEHTIVVDAPSWGQDWQYLMRDNAQEIYDADPTGNTVFSVHMYQVFG.TPASVTNYLEYFL.DRDLPIIVGEFGWE..HQNE......VVAWEAILSESERLRTGYLGWSWSGNTE..
ANZ43360.1  PSAWVGETKSAIARMRKEGLTHTLVADGPNWGQDWSFVMRDNAKQIFNSDVRRNTVFSVHMYEVFG.TADKVNNYLNRFA.AARLPIVVGEFGHW..HGNA......SVDEDAIFAASRRLGTGFLAWSWSGNGGG.
ACS46797.1  ASQWTQGTKDAIRQMRDAGFRHTLVLTAPNWGQDWQGVMKDNAKSVYS..QDSNVLFDVHMYGVYS.TADKVKSYIDSFV.NQGLPLMVGEFGNK..HSDG......IPDAATIMSYTKEKGVGMLAWSWCGNTEGV
AEK30917.1  ASQWTQGTKDAIRQMRDAGFRHTLVLTAPNWGQDWQGVMKDNAKSVYS..QDSNVLFDVHMYGVYS.TADKVKSYIDSFV.NQGLPLMVGEFGNK..HSDG......IPDAATIMSYTKEKGVGMLAWSWCGNTEGV
AGB26659.1  TAEWSSATQSAIHKLRDAGFRHTLIVDGPDAGEDRSFAMRDNAPGILNDDPDHNILFSVHMYGAFN.NPAAVASYLDSFQ.TRGIPLLVGEFGDN..SDDPNQ..DIKSDANSIMAEAVRRGIGYLGWCWSGND...
AGB26661.1  GDKWVADTESAIQKVRGAGLRHTLVIDPPNYGQDTQFIMRDNASQVLASDPEHNVVFSVHMYGAFV.SPDKVISYMDSFQ.TRRLPLVVGEFGYS..ADDVNL..AVRSDADTIMAESVRRGIGYLGWCWSGN....
UAB95210.1  DWPWSQQTAAAVRALRAAGFKHTLMVDAPGWGQDEAFVMRDNAEQIRAADPAGNTVFDIHMYGEFD.NAAKVNSYLGAYT.RRKLPIIIGEFSGE..HEWG......DPDEDAIMAYAEGHSVGYFGWSWSGNDK..
SJX74184.1  TEHWLDSYTEAVKVLRDAGYKTTIVVDSPGWGQNIN.PILEAGKELIENDLEHNILFSVHMYGSWN.NPEKIKTDLNRAK.RLKLPLIVGEFGYN..CRNGDNNLGCKADHTVILSTCTELDYGYMPWSWTGNNAE.
ASS64945.1  DTAWRDAYQTAISTLRSGGINNTIVIDGSGWGQNSS.PIKAYGQTLLNYDPKHNVMFSIHMYGSWN.DSSRIGTELQAIK.NLGLAVTVGEFGYNYNNGSNNL..GCKVNAQEVLNQAQAKGIGYLAWSWTGNDSA.
AET57908.1  DAAWRDAYKTAITTLRNAGLPNTIVIDGSGWGQYAS.PIKAHGVELLQHDPNHNLLFSVHMYANYN.DSSKIGSELQAIK.DKGLAVIVGEFGYNYNNGNNNL..GTTVNPYEVMKQSQAKGIGYLAWSWTGNNSEN
AXA90579.1  PLQWRDDYKRPITTIRNTGLTTTLVIDNPDYGGNPN.GGLWYGQDLLEHDPRHNLLFSVHMYSTFN.NPDDIYNTIVNYR.NGQLPLLIGEFGYNYNNGANNL..NSRVDAARLMEWAQQFGVGTVAWSTAGNDGP.
AGX43683.1  STVWRDGYKNAVSRIRTAGYTGTIVIDAGGWGQDSN.DILKYAKDIYNCNTNKNVIFSIHMYGSWN.NNSSIDSFLRSCK.SKGIPIIVGEFGYNYNNGSNNL..GCKVDVAHLINYCKQNKIGFIAWSWSGNDSDN
ACY02072.1  DTIYRDAYKEAITMMRNAGLKHTIVIDGSGWGQDMT.PIFDYGQEILNHDPQKNILFSVHMYENYR.NESTITTNLEKAV.DMKLPLIVGEFGFQ..HGGTAD.NPIQIPYTHILSECERLGLGWYAWSWKGNSGGV
AZQ63976.1  DAVYRDTYTEAVTNLRNAGIMHTLVIDASGWGQDIQ.HVFDYGQNIIDSDPLKNILFSVHMYQSFR.TEQSITAALEKAV.ELKLPLVVGEFGFK..HGDPA....VDIPFEHILSECERLEIGWLAWSWKGNSGGV
ADX05709.1  AANWKDGYVKAIPIIRKAGLRHCIMVDAGGYGQSAA.TIHTYGKDVLAADPENNVLFSIHMYGTAG.NKNRVKSNIDGVI.NQGLALCIGEFGWY..HSDG......DVDEDQILSYCQEKNVGWLAWSWYGNGSPV
```
