# Supplementary material for: Impact of Modular Architecture on Activity of Glycoside Hydrolase Family 5 Subfamily 8 Mannanases
Source: Molecules. 2022 Mar 16;27(6):1915. doi: 10.3390/molecules27061915 (PMC8952944; doi:10.3390/molecules27061915)

Figure S2. Phylogenetic tree with accession and domain family numbers. The tree is identical with the tree in Figure 3, but here information about NCBI accession numbers and domain family is available.

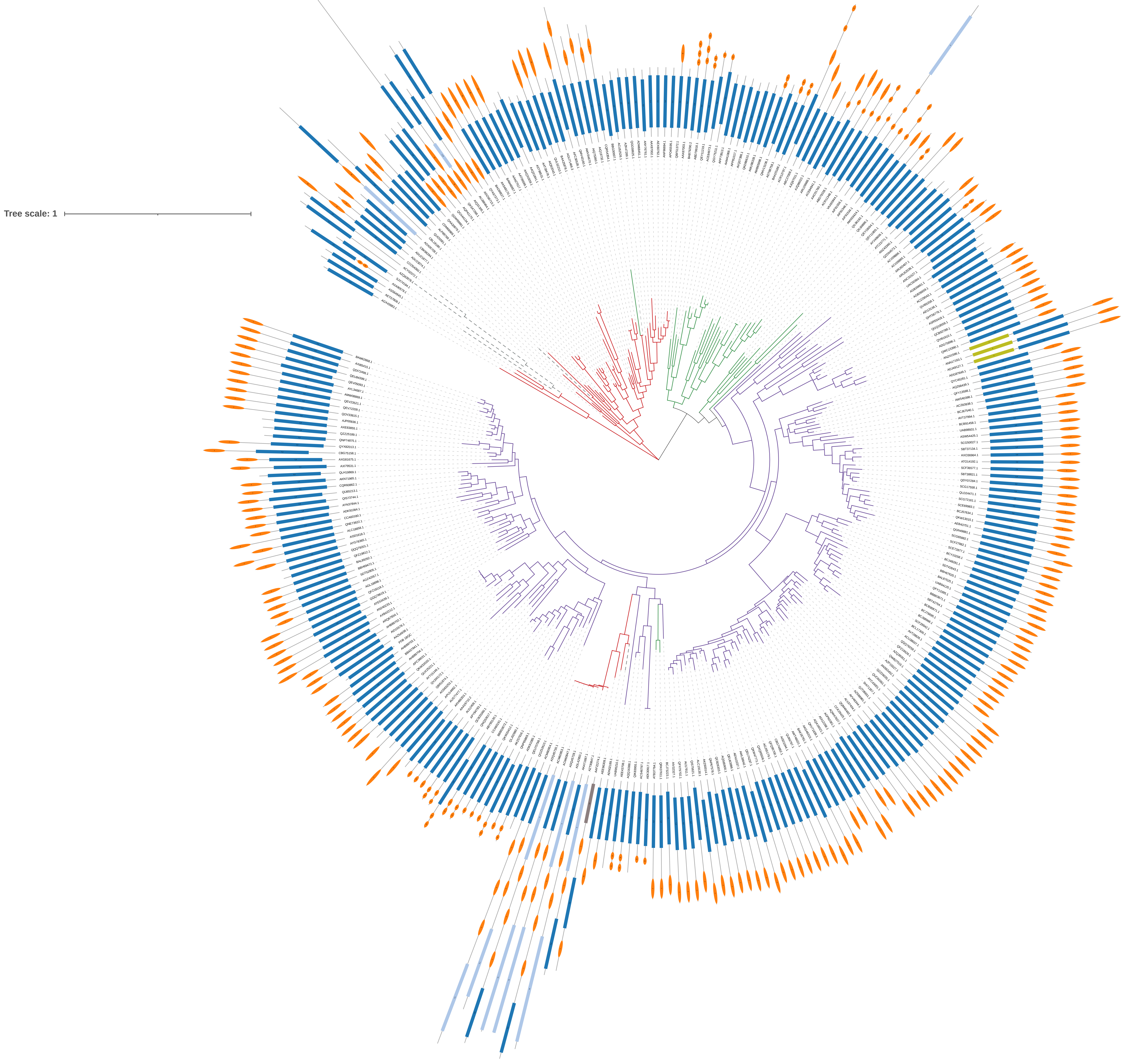

Supplement: Supplementary file 1 [file molecules-27-01915-s001.zip › Figure_S2.pdf]
