# Supplementary figures and images for: Impact of Modular Architecture on Activity of Glycoside Hydrolase Family 5 Subfamily 8 Mannanases
Source: Molecules. 2022 Mar 16;27(6):1915. doi: 10.3390/molecules27061915 (PMC8952944; doi:10.3390/molecules27061915)

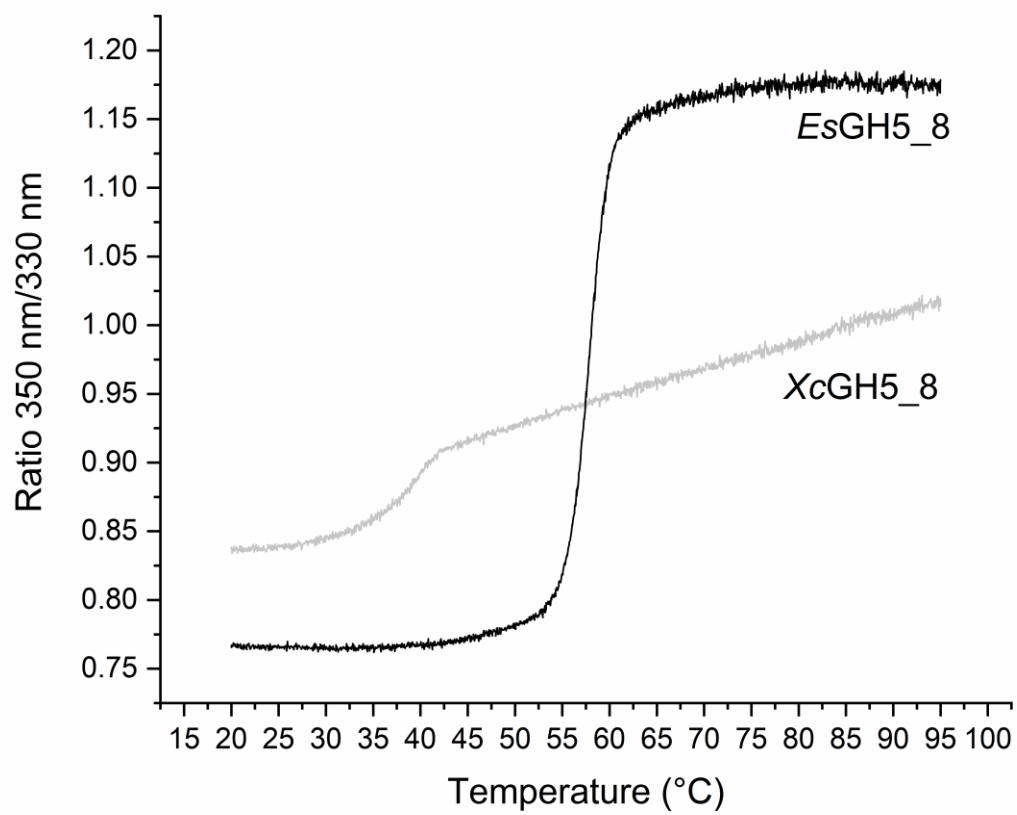

**Figure S3.** Differential scanning fluorimetry (DSF) results for *EsGH5\_8* and *XcGH5\_8*.

Supplement: Supplementary file 1 [file molecules-27-01915-s001.zip › Figure_S3.pdf]

(A)

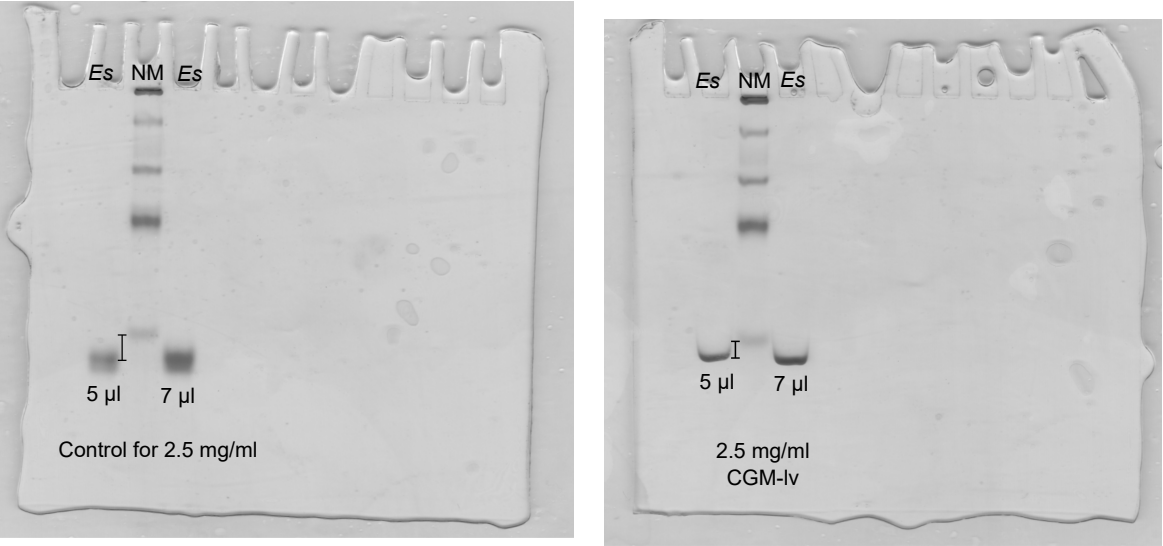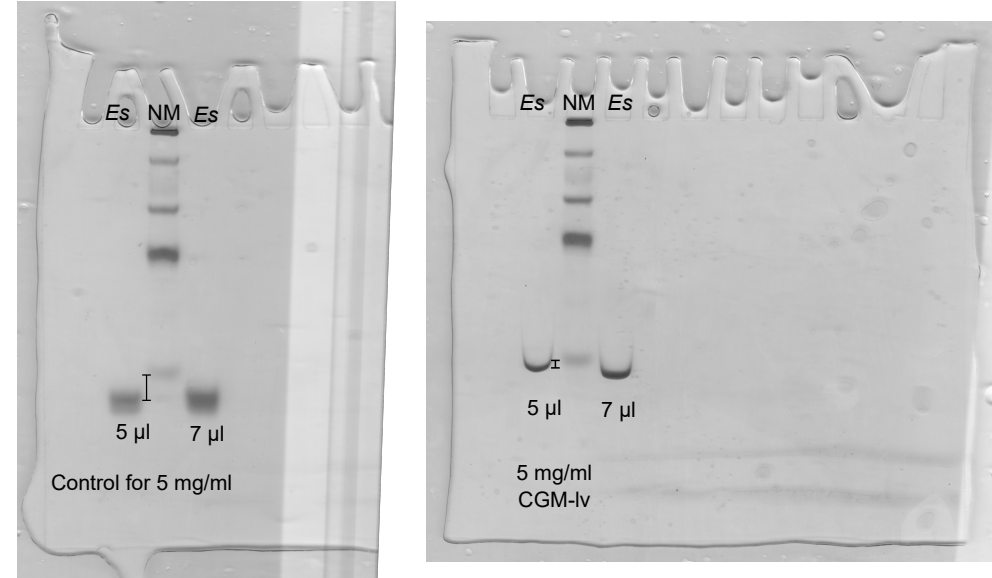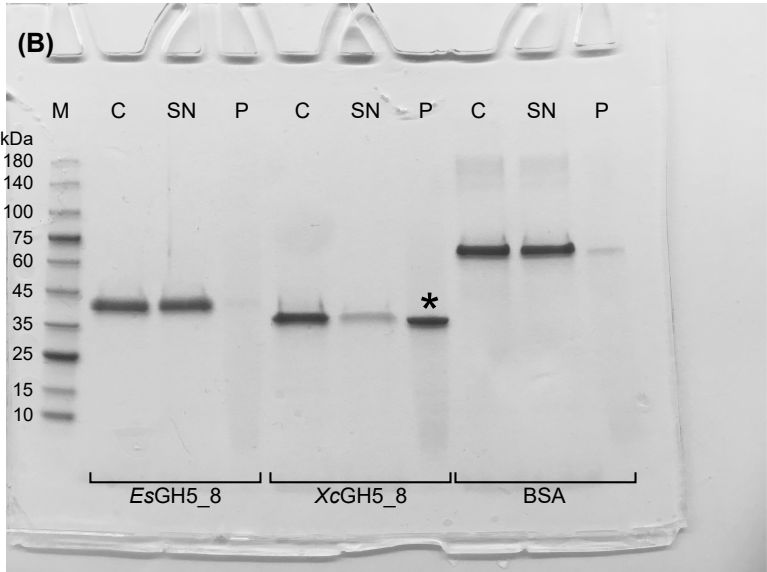

Figure S4. Original AGE gels related to Figure 5.

Supplement: Supplementary file 1 [file molecules-27-01915-s001.zip › Figure_S4_original_AGE_gels.pdf]
